# Post-translational modifications of immune checkpoints: molecular mechanisms, tumor microenvironment remodeling, and therapeutic implications

**DOI:** 10.1186/s12929-025-01202-1

**Published:** 2026-01-04

**Authors:** Hung-Chia Hsieh, Lun-Ling Ling, Yi-Ching Wang

**Affiliations:** 1 Institute of Basic Medical Sciences, College of Medicine, National Cheng Kung University, Tainan, 70101 Taiwan; 2 Department of Pharmacology, College of Medicine, National Cheng Kung University, No.1, University Road, Tainan, 70101 Taiwan, ROC

**Keywords:** Post-translational modifications, Immune checkpoints, Tumor microenvironment, Cancer immunotherapy, Treatment resistance, Targeted therapy, Combination therapy

## Abstract

Immune checkpoints play pivotal roles in regulating immune responses and maintaining tolerance. In cancer, these molecules are hijacked to suppress antitumor immunity, resulting in therapeutic resistance to immune checkpoint blockade (ICB). Recent advances have highlighted the critical role of post-translational modifications (PTMs), including phosphorylation, ubiquitination, glycosylation, palmitoylation, UFMylation, acetylation, SUMOylation, methylation, and ISGylation, in modulating checkpoint stability, trafficking, and function across diverse immune and tumor cell types. These dynamic PTMs reshape the tumor microenvironment (TME) by controlling immune cell function, antigen presentation, and inflammatory signaling. This review comprehensively outlines the mechanistic contributions of PTMs to immune checkpoint regulation, emphasizing how these PTMs orchestrate immune evasion and clinical outcomes. Special focus is given to PTMs of PD-L1, PD-1, TIM-3, TIGIT, CTLA-4, LAG-3, VISTA, BTLA, and SIRPα. We also discuss how targeting PTM-regulating enzymes or specific modification motifs offers a promising therapeutic strategy to overcome ICB resistance. Understanding the PTMs landscape provides critical insight into resistance mechanisms and unveils promising opportunities for rational combination therapies aimed at reprogramming the immunosuppressive TME and enhancing antitumor immunity.

## Background

Immune checkpoint molecules (checkpoints) are pivotal regulators of immune homeostasis, acting to prevent excessive immune activation and maintain peripheral tolerance. However, in the context of cancer, these checkpoints are often occupied by tumors to suppress anti-tumor immune responses and promote immune escape [1]. Within the tumor microenvironment (TME), checkpoints such as programmed death ligand-1 (PD-L1), programmed cell death-1 (PD-1), T cell immunoglobulin and mucin-domain containing-3 (TIM-3), T cell immunoreceptor with Ig and ITIM domains (TIGIT), cytotoxic T-lymphocyte-associated protein 4 (CTLA-4), lymphocyte activation gene-3 (LAG-3), V-domain immunoglobulin suppressor of T cell activation (VISTA), B and T lymphocyte attenuator (BTLA), and signal regulatory protein alpha (SIRPα) are frequently upregulated on T cells and other immune cell subsets, and emerged as key immunosuppressive regulators to dampen anti-tumor immunity [2–8]. As a result, these checkpoints have emerged as central targets in cancer immunotherapy.

To date, numerous immune checkpoint blockade (ICB) therapies have received FDA approval for use in cancer treatment, including two CTLA-4 inhibitors (ipilimumab and tremelimumab), eight PD-1 inhibitors (nivolumab, pembrolizumab, cemiplimab, dostarlimab, retifanlimab, toripalimab, tislelizumab, and penpulimab), four PD-L1 inhibitors (atezolizumab, durvalumab, avelumab, and cosibelimab), and one LAG-3 inhibitor (relatlimab) [9]. Additional checkpoint inhibitors targeting TIGIT, TIM-3, VISTA, and BTLA are currently undergoing clinical trials, suggesting the continued expansion of immunotherapy development [10]. Despite the remarkable and durable responses observed in a subset of patients, the response rate remains low [9]. Many patients exhibit either primary resistance, often associated with poor immune cell infiltration or pre-existing immunosuppressive features, or acquired resistance following an initial response. A key mechanism of adaptive resistance involves the compensatory upregulation of alternative checkpoints [9]. For example, increased TIM-3 expression on T cells and myeloid-derived suppressor cells (MDSCs) has been linked to resistance to anti-PD-1 therapy in both preclinical models and patient samples. Notably, co-blockade of TIM-3 has been shown to restore sensitivity to PD-1 blockade in murine models of lung cancer, highlighting the promise of combinatorial checkpoint inhibition strategies [11, 12]. Similarly, the combination of relatlimab (an anti-LAG-3 agent) with nivolumab (an anti-PD-1 agent) has shown enhanced therapeutic benefits in patients with advanced melanoma. This dual therapy was found to enhance CD8^+^ T cell receptor signaling and shift T cell differentiation toward an effector phenotype [13]. Collectively, these findings emphasize the complex interplay between different immune checkpoint pathways and underscore the need for rationally designed combination therapies.

Increasing evidence highlights the importance of post-translational modifications (PTMs) in modulating checkpoint biology, such as protein function, stability, and intracellular trafficking. PTMs, including phosphorylation, ubiquitination, glycosylation, palmitoylation, UFMylation, acetylation, SUMOylation, and methylation, provide an additional layer of regulatory control, enabling checkpoints to respond rapidly to external cues without altering gene expression [14–16]. These PTMs not only determine checkpoint receptor stability and subcellular localization but also impact receptor-ligand affinity, membrane retention, and downstream inhibitory signaling [14–16]. PTMs serve as dynamic switches that integrate extracellular stimuli and intracellular responses, acting in a context- and cell type-specific manner. Within the TME, checkpoints are expressed not only by T cells but also by macrophages, dendritic cells (DCs), natural killer (NK) cells, B cells, and even tumor cells [2]. The PTMs of checkpoints in these diverse cellular compartments shape immune outcomes by modulating processes such as antigen presentation, cytokine secretion, macrophage polarization, and the activation of innate sensing pathways [17] (Table 1).

**Table 1:** PTMs of immune checkpoints

Protein	PTM	Modifying enzyme	Site	Function	References
PD-L1	Phosphorylation	GSK3β	T180, S184	Promotes β-TrCP interaction with PD-L1	[26]
		GSK3α	S279, S283	Promotes ARIH1 interaction with PD-L1	[27]
		AMPK	S283, S195	Enhances PD-L1 degradation	[28]
		JAK1	Y112	Recruits STT3A and promotes glycosylation	[30]
		CK2	T285, T290	Prevents CUL3-mediated proteasomal degradation	[31]
		LRRK2	T210	Promotes PD-L1 stability	[32]
		DNA-PKcs	T20, T22	Promotes PD-L1 stability	[33]
	Ubiquitination	β-TrCP	–^a^	Enhances PD-L1 proteasomal degradation	[26]
		SPOP	–^a^	Enhances PD-L1 proteasomal degradation during the cell cycle	[34]
		HUWE1	K281	Enhances ER-associated degradation	[38]
		ARIH1	K271, K281	Enhances PD-L1 proteasomal degradation	[27]
		NEDD4	–^a^	Enhances PD-L1 proteasomal degradation	[39]
		KEAP1	–^a^	Enhances PD-L1 proteasomal degradation	[40]
		MIB2	K136	Promotes PD-L1 trafficking to the plasma membrane	[47]
		Skp2	K136, K280	Recruits LKB1 and stabilizes PD-L1	[48]
	Deubiquitination	CSN5	–^a^	Removes K48-linked ubiquitin	[49]
		USP9X	–^a^	Stabilizes PD‐L1	[50]
		USP22	–^a^	Removes K6, K11, K27, K29, K33, and K63-linked ubiquitin	[51]
		USP7	–^a^	Stabilizes PD‐L1	[52]
		USP2	K263, K271, K280, K281	Removes K48-linked ubiquitin	[53]
		OTUB1	–^a^	Protects PD-L1 from the ERAD	[54]
		USP10	–^a^	Stabilizing both cytosolic and nuclear PD-L1	[55]
		USP24	–^a^	Stabilizes PD‐L1	[56]
	Glycosylation	B3GNT3	N192, N200	Promotes interaction with PD-1	[57]
		STT3A	–^a^	Stabilizes PD‐L1	[58]
		GLT1D1	–^a^	Stabilizes PD‐L1	[62]
		B4GALT1	N192, N200	Stabilizes PD‐L1	[63]
		GALNT6	–^a^	Stabilizes PD‐L1	[64]
		MGAT5	N35, N200	Promotes interaction with PD-1	[65]
		ST3GAL4	–^a^	Promotes PD-L1 sialylation and its interaction with CD169	[68, 69]
		CHST8	N35, N192, N200	Suppresses T cell activation	[70]
	Palmitoylation	DHHC3	C272	Blocks mono-ubiquitination and inhibits lysosomal degradation	[71]
		DHHC9	C272	Stabilizes PD‐L1	[72]
	UFMylation	UFL1	K89, K162	Promotes PD‐L1 proteasome-mediated degradation	[74]
	Acetylation	P300	K263	Interrupts PD-L1 nuclear translocation	[76]
	SUMOylation	TRIM28	–^a^	Inhibits ubiquitin-mediated proteasomal degradation	[77]
	Methylation	SETD7	K162	Interrupts interaction with PD-1	[78]
	ISGylation	–^a^	–^a^	Increases K48-linked ubiquitination	[79]
PD-1	Phosphorylation	Fyn, Lck	Y248	Recruits tyrosine phosphatase SHP-2 and T cell	[96]
		CDK1	S261	Promotes nuclear translocation and interaction with FBW7	[99]
		ERK	T234	Promotes binding to USP5	[100]
	Ubiquitination	FBXO38, FBW7	K233	Enhances proteasomal degradation	[101]
		KLHL22	K210, K233	Enhances proteasomal degradation	[99, 102]
		MDM2	K78	Enhances proteasomal degradation	[104]
		c-Cbl	–^a^	Enhances proteasomal degradation	[103]
		TRIM21	K233	Catalyzes the K63-linked ubiquitin to antagonize K48-linked ubiquitination and degradation	[105]
	Deubiquitination	USP5	–^a^	Promotes PD-1 stability	[100]
		USP24	–^a^	Promotes PD-1 stability	[106]
	Glycosylation	B3GNT2	N49, N58, N74, N116	Promotes PD-1 protein stability and ligand PD-L1 binding affinity, and antibody binding efficacy	[108]
		FUT8	N49 and N74	Promotes PD-1 stability	[110]
	Palmitoylation	DHHC9	C192	Facilitates association with RAB11, promotes its localization in recycling endosomes, and protects it from lysosomal degradation	[113]
	UFMylation	UFL1	K210, K233	Antagonizes K48-linked ubiquitination and promotes PD-1 stability	[114]
TIM-3	Phosphorylation	Fyn, Lck, PTPN2	Y256	Ligand-induced phosphorylation releases Bat3 and recruits SH2 kinases; Suppresses TCR signaling; Promotes T-cell exhaustion	[131]
	Ubiquitination	β-TrCP	–^a^	Promotes plasma membrane translocation, PI3K binding, mTOR inhibition; Enhances M2 polarization and TGF-β secretion in macrophages	[137]
	Glycosylation	–^a^	N78, N100	Required for folding, ER export, and Galectin-9/PD-1 lattice formation; Facilitates immunosuppressive interactions in TME	[138]
	DNA Methylation	DNMT1, DNMT3A	HAVCR2 promoter CpG	Hypomethylation increases TIM-3 expression, while methylation silences expression and promotes immune evasion	[140]
	Histone Methylation	SUV39H1, DNMT3A	H3K9 (via H3K9me3)	Represses HAVCR2 transcription; reversible by demethylating agents (e.g., 5-aza-dC)	[141]
	Palmitoylation	DHHC9	C296	Stabilizes membrane localization in exhausted T cells; Inhibition restores cytokine secretion and effector function	[143]
TIGIT	Phosphorylation	Lck, Fyn	Y225, Y231	Phosphorylation recruits SHIP-1, Grb2, β-arrestin-2; Suppresses AKT/MAPK/NF-κB; Modulates CD226 signaling	[157]
	Ubiquitination	STUB1 (CHIP)	K48-linked (exact site unreported)	Drives proteasomal degradation under hypoxia (may depend on ITIM phosphorylation)	[162]
	Glycosylation	OST complex (putative)	N32, N101	Ensures folding and CD155 binding, while loss of this function impairs inhibitory function	[165]
	Methylation	DNMT1, DNMT3A	Promoter region	Hypomethylation enhances TIGIT transcription in TILs; methylation correlates with prognosis and is reversible with DNMT inhibitors	[170]
	SUMOylation	UBC9 (E2); PIAS family (putative E3)	–^a^	Stabilizes CD155 surface expression; Enhances TIGIT binding; Promoting immune suppression	[171, 172]
CTLA-4	Phosphorylation	Fyn, Lyn, Lck	Y201	Prevents clathrin-mediated endocytosis; Recruits SHP2 and induces T cell inactivation	[181, 182]
	Ubiquitination	TRAF6	K203, K213	Enhances lysosomal degradation	[191]
	Glycosylation	–^a^	N110	Promotes binding of humanized antibody mAb146	[193]
LAG-3	Phosphorylation	–^a^	F475, L478	Exerts inhibitory function	[203]
	Ubiquitination	LUBAC	K21, K297, K356, K366, K498	–^a^	[204]
		c-Cbl, Cbl-b	K498	Promotes LAG-3 immunosuppressive function	[205]
VISTA	Phosphorylation	Presumed Src-family kinases (putative)	Predicted motifs: YxxQ, PxxP	May regulate receptor internalization, signal transduction, or ubiquitin priming (phosphodegron recognition)	[228, 229]
	Ubiquitination	TRIM28	Unspecified (K63-linked)	Stabilizes VISTA in microglia; Promotes glycolysis via HK2 and M2-like polarization	[232]
	Glycosylation	ER-resident OSTs (predicted)	N49, N91, N108, N128, N135	Required for folding, surface expression, and ligand binding; Loss reduces membrane stability and activity in RCC	[233]
	Histone Acetylation	H3K27ac	*Vsir* promoter/enhancer regions	LPS reduces H3K27ac, decreasing chromatin accessibility and VISTA transcription in microglia	[235]
BTLA	Phosphorylation	Fyn, Lyn, Lck	Y226, Y243, Y257 and Y282	ITIM/ITSM phosphorylation recruits SHP-1/2 to suppress TCR signaling; Y226/Y243 phosphorylation via Grb-2–PI3K promotes T cell activation	[239, 240]
	Glycosylation	N-glycan branching	N75, N94, and N110	N-glycan branching reduces surface expression via endocytosis, while reduced branching increases retention and inhibitory activity	[241]
SIRPα	Phosphorylation	Fyn, Lck	Y429, Y453, Y470, Y496; possibly Y501	Recruits SHP1/2 upon CD47/integrin binding; Suppresses PI3K-Akt2, MAPK, NF-κB; Inhibits phagocytosis	[258]
	Ubiquitination	TRIM21, Cbl, NEDD4 (putative)	Lysine residues (unspecified)	Ubiquitination not directly shown; CD47 ubiquitination may influence SIRPα turnover or signaling	[261, 262]
	Glycosylation	–^a^	Asn-linked glycans (unspecified)	Promotes cis-dimerization and clustering; Regulates surface organization and inhibitory activity	[265]
	Acetylation	HDAC6	Lysine residues (unspecified)	Deacetylation by HDAC6 enhances lysosomal degradation, promoting macrophage phagocytosis and synergy with anti-CD47	[68]
	Neddylation	NEDD8/CRL pathway (indirect)	–^a^	Inhibition of CRLs promotes SIRPα internalization and degradation; Boosts innate immunity	[270]

–^a^Not reported

This review focuses on emerging insights into the PTMs of checkpoints. We discuss the mechanistic role of each PTM in checkpoint biology and explore how these molecular events contribute to TME remodeling. A deeper understanding of these dynamic regulatory processes could uncover novel therapeutic strategies that target PTM-modifying enzymes or checkpoint-specific PTM motifs to enhance antitumor immunity **(**Table 2**)**.

**Table 2:** Potential drugs targeting PTMs of checkpoints and their clinical status

Protein	Potential drug	Function	Clinical status	Limitations	References
PD-L1	ES-072	Activates GSK3α to recruit ARIH1 to destabilize PD-L1	–^a^	–^a^	[27]
	CX4945	Inactivates CK2 to promote CUL3-mediated PD-L1 degradation	–^a^	–^a^	[31]
	GSK2578215	Inactivates LRRK2 to inhibit PD-L1 degradation	–^a^	–^a^	[32]
	Metformin	Activates AMPK and induces abnormal PD-L1 glycosylation and degradation	Approved	Limited efficacy in cancer treatment	[29]
	A-769662	Activates AMPK to disrupt PD-L recycling back to the plasma membrane	–^a^	–^a^	[28]
	Canagliflozin	Activates AMPK to disrupt PD-L recycling back to the plasma membrane	Phase 1	Patients with colorectal cancer are being recruited	[37]
	Gefitinib	Inhibits EGF-mediated GSK3β/β-TrCP inactivation	Approved	Significant toxicities and the emergence of drug resistance	[26]
	Etoposide	Downregulates STT3A expression	Approved	Dose-limiting side effects such as myelosuppression	[58]
	STM108	Specifically recognizes glycosylated PD-L1 and induces PD-L1 internalization and degradation	–^a^	–^a^	[57]
	ML364	Inhibits USP2 activity and destabilizes PD-L1	–^a^	–^a^	[53]
	Curcumin	Inhibits CNS5 activity and destabilizes PD-L1	Phase 2	Poor bioavailability	[54]
	PTPR	Disrupts the interaction between TMUB1 and PD-L1	–^a^	–^a^	[38]
	NU-7441	Inhibits DNA-PK activity and decreases PD-L1 stability	–^a^	–^a^	[33]
	2-Bromopalmitate	Inhibits the PD-L1 palmitoylation	–^a^	–^a^	[68]
PD-1	Oridonin	Activates FBW7 activity and induces PD-1 degradation	–^a^	–^a^	[100]
	IL-2	Upregulates FBXO38 expression and induces PD-1 degradation	Approved	Short half-life and severe vascular toxicity	[102]
	IFN-α	Upregulates MDM2 expression and induces PD-1 degradation	Approved	Nonspecific stimulation of inflammation pathways and off-target effects on non-neoplastic tissues	[104]
	EOAI3402143	Inhibits USP5 activity and induces PD-1 degradation	–^a^	–^a^	[100]
	Trametinib	Inhibits MEK activity and reduces the interaction of USP5 with PD-1	Approved	Mainly in combinations	[100]
	USP24-i-101	Inhibits USP24 activity and induces PD-1 degradation	–^a^	–^a^	[106]
	2-fluoro-L-fucose	Inhibits the natural GDP-Fuc production and PD-1 fucosylation	–^a^	–^a^	[110]
	PD1-PALM	Disrupts the interaction of DHHC9 with PD-1	–^a^	–^a^	[113]
	MK-8722	Disrupts PD-1 UFMylation and triggers degradation	–^a^	–^a^	[114]
TIM-3	Palmitoylation-blocking peptide	Blocks the TIM-3 extracellular domain; relieves T-cell exhaustion	Pre-clinical	Insufficient data; peptide stability and in vivo delivery challenges	[143]
	BGB-A425	Targets the IgV domain; enhances CD8⁺ T cell response	Phase 1/Phase 2	Limited monotherapy benefits and mainly in combinations	[279]
	INCAGN02390	Antagonistic antibody targeting ligand-binding site; boosts T and NK cell activity	Phase 1	Off-target risks; often combined with other ICBs	[280]
	LY3321367	Blocks TIM-3; reduces immunosuppression and increases IFN-γ production	Phase 1a/1b	Suboptimal efficacy to date	[279]
	TSR-022	Human mAb blocking TIM-3; restores effector T cell function	Phase 3	Limited monotherapy benefits and mainly in combinations	[279]
	Sym023	Bispecific antibody (TIM-3/PD-1); overcomes T cell exhaustion	Phase 1/1b	Off-target risks; combo-dependent	[277]
	RO7121661	Bispecific antibody (TIM-3/PD-1); dual blockade synergizes immunity	Phase 2	Immunogenicity and safety unresolved	[281]
	LY3415244	Soluble decoy blocking TIM-3; restores CD8⁺ T cell cytotoxicity	Phase 1a/1b	Limited benefit with suboptimal efficacy	[281]
	ML-T7	Humanized IgG4 anti-TIM-3 antibody that blocks ligand binding; promotes T and myeloid cell reprogramming	Pre-clinical	Limited evidence; stability issues	[282]
	MBG453 (sabatolimab)	Blocks TIM-3; relieves exhaustion	Phase 3	Heterogeneous activity; safety/PK concerns	[278]
TIGIT	Tiragolumab	FcγR-mediated TAM/DC activation; increases CD8⁺ T cell memory	Phase 3	Limited efficacy in Phase 3	[284]
	Ociperlimab (BGB-A1217)	Fc-competent antibody blocking TIGIT–CD155; engages myeloid cells	Phase 1/2	Heterogeneous activity; safety/PK concerns	[285]
	Liothyronine	Small-molecule disruptor of TIGIT–PVR binding	Pre-clinical	Systemic toxicity risk	[290]
	Hemin	Blocks TIGIT–PVR; restores T cell function	Pre-clinical	Limited evidence; stability issues	[291]
	Azelnidipine	Inhibits TIGIT–PVR; promotes activation	Pre-clinical	Limited evidence; stability challenges	[292]
	9-ING-41 (Elraglusib)	GSK3β inhibitor; indirectly blocks TIGIT; increases cytokine production	Phase 2	Off-target risks; combo-dependent	[293]
	Gln(TrT)	Dual TIGIT/PD-1 inhibitor; restores CD8⁺ T cell function	Pre-clinical	Systemic adverse effect risk	[294, 295]
	D-peptides	Peptide-based TIGIT–PVR disruptors; restore T cell function	Pre-clinical	Limited evidence; stability issues	[297]
	TAK-981	SUMOylation inhibitor; decreases CD155 expression; enhances TIGIT blockade	Phase 1/2	Systemic toxicity	[172]
	Domvanalimab (AB-154)	Blocks TIGIT–PVR; synergizes with PD-1 blockade	Phase 3	Limited monotherapy benefit	[298]
	Vibostolimab (MK-7684)	Blocks TIGIT–PVR; relieves T cell exhaustion	Phase 2/3	Heterogeneous activity; safety/PK concerns	[299]
	EOS-448	Blocks TIGIT–PVR/CD112; promotes T cell and myeloid modulation	Phase 2	Toxicity risks	[300]
	COM-902	TIGIT–PVR inhibitor, designed for reduced toxicity	Phase 1	Safety concerns	[301]
	BMS-986207	Inhibits TIGIT–PVR; modulates immune response	Phase 1/2	Safety concerns	[302]
	ASP-8374	Selective TIGIT–ligand blocker; reduces Treg suppression	Phase 1	Off-target risks	[303]
	Etigilimab (OMP-313M32)	Restores CD8⁺ T cell function; tested in nivolumab combinations	Phase 1/2	Toxicity risks	[305]
	M6223	Blocks ligand binding; depletes TIGIT⁺ Tregs	Phase 1	Immunogenicity and safety unresolved	[306]
CTLA-4	OX86	Induces TRAF6 expression and accelerates CTLA-4 degradation	–^a^	–^a^	[194]
	mAb146	binds to N110 on glycosylated CTLA-4	–^a^	–^a^	[302]
	D11	Inhibits interaction with CD80	–^a^	–^a^	[303]
LAG-3	Eftilagimod alpha	A soluble LAG-3 protein acts as an antigen-presenting cell activator	Phase 1/2	A low proportion of patients had treatment-related adverse events	[309, 310]
VISTA	BMS767	pH-selective antibody targeting C–C’ loop; enhances IFN-γ production and T cell proliferation	Pre-clinical	Off-target risks; often used in combinations	[311]
	SG7	Blocks ligand binding; restores NFAT signaling; reduces suppressive PMN-MDSCs	Pre-clinical	Limited evidence	[310]
	VSTB112	Blocks ligand interaction across the pH range; reverses T cell suppression	Phase 1	Limited monotherapy benefit; mainly in combinations; safety concerns	[312, 313]
	CI-8993	FcγR-mediated depletion of VISTA⁺ myeloid cells	Phase 1	Limited monotherapy benefit; mainly in combinations	[314, 315]
	HMBD-002	Fc-inert IgG4 antibody disrupting VISTA–VSIG3/HVEM binding	Phase 1	Limited evidence	[316]
	KVA12123	Blocks multiple ligands across pH range	Phase 1/2	Limited monotherapy benefit; mainly in combinations	[317]
	JNJ-61610588	Fully human IgG1κ antibody; classical blockade of VISTA	Phase 1	Limited benefit in trials; safety and tolerability issues	[318]
	CA-170	Oral small-molecule dual inhibitor of VISTA/PD-L1; blocks VISTA–VSIG3	Phase 1	Limited evidence	[319]
	Chidamide (HBI-8000)	Oral HDAC inhibitor; blocks VISTA–PSGL-1 at acidic pH; enhances CD8⁺ T cell function	Phase 2b	Systemic toxicity	[222]
	Compound S8	Bifunctional small molecules targeting PD-L1 and VISTA; activates tumor immunity	pre-clinical	Limited evidence; stability and delivery issues	[313]
	SNS-101	pH-selective antibody; inhibits VISTA in acidic TME; improves PD-1 efficacy	Phase 1	Limited evidence	[209]
BTLA	Icatolimab (JS004/TAB004, Tifcemalimab)	Humanized IgG4 antibody blocking BTLA–HVEM interaction; restores T cell activity; synergizes with PD-1 blockade	Phase I/II	Limited clinical data; efficacy varies by tumor type; mainly in combinations	[322]
	Anti-HVEM antibodies	Block HVEM-mediated inhibition of BTLA/CD160; reprogram T cell responses	Pre-clinical	Limited in vivo validation; specificity concerns	[326]
	HVEM-Fc fusion proteins	Soluble HVEM decoys; block BTLA inhibitory engagement; rewire HVEM signaling	Pre-clinical	In vivo delivery and stability challenges	[326]
	22B3, 25F7, 23C8	Structure-guided antibodies defining epitopes; inform design of selective BTLA antagonists	Pre-clinical	Tool/structural antibodies; not yet therapeutic	[327]
SIRPα	ES004-B5, KWAR23	Pan-allelic neutralizing antibodies blocking SIRPα–CD47; reprogram TAMs/DCs to inflammatory state; enhance phagocytosis	Pre-clinical	Limited clinical data; need PD-1/PD-L1 combination	[331]
	BI765063	Fc-modified SIRPα fusion protein; promotes macrophage activation and phagocytosis	Phase 1/1b-2	Limited monotherapy benefit; mainly in combinations	[331]
	ALX148 (Evorpacept)	High-affinity SIRPα-Fc fusion with inactive Fc; blocks CD47 and enhances antitumor immunity	Phase 2/3	Tumor-type dependent activity (hematologic vs solid)	[332]
	TTI-621, TTI-622	Recombinant SIRPα-Fc proteins with IgG1/IgG4 backbones; promote macrophage-mediated phagocytosis	Phase 1/2	Limited monotherapy benefit; mainly in combinations	[333]
	OAd-SIRPα-Fc	Local intratumoral delivery; reprograms TAMs/DCs; boosts CD8⁺ T cells	Pre-clinical	Limited evidence; in vivo delivery challenges	[330]
	NCGC00138783	Small-molecule antagonist from qHTS; binds SIRPα interface and blocks CD47	Pre-clinical	Specificity and in vivo stability challenges	[273]
	NCGC00538430, NCGC00538419	Additional small molecules targeting SIRPα from same screen	Pre-clinical	Limited pharmacokinetic data	[273]
	SMC18	Dual small-molecule inhibitor of SIRPα–CD47 and PD-1–PD-L1; enhances phagocytosis and T cell activity	Pre-clinical	Stability and delivery issues	[334]
	NSC622608	Inhibits NEDD8-activating enzyme; disrupts CRLs; induces apoptosis and DNA damage	Pre-clinical	Tumor-type dependent activity	[335]
	MLN4924 (Pevonedistat)	Inhibits NEDD8-activating enzyme, disrupting CRLs and inducing immune-related apoptosis and DNA damage	Phase 3	Toxicity (hepatic, myelosuppression)	[335]

## TME remodeling and PTMs of checkpoints

### PD-L1

Programmed death ligand-1 (PD-L1), encoded by the *CD274* gene, is a pivotal checkpoint that plays a central role in immune regulation and tumor immune evasion. PD-L1 is predominantly expressed on the surface of tumor cells. It is also widely present on various immune cell subsets, including B cells, NK cells, CD4⁺ T cells, CD8⁺ T cells, Tregs, DCs, and tumor-associated macrophages (TAMs). When expressed on tumor cells or antigen-presenting cells (APCs), PD-L1 engages PD-1 receptors on activated T lymphocytes, delivering inhibitory signals that suppress T cell activation, proliferation, and cytokine production, thereby promoting T cell exhaustion and functional impairment [1]. Therapeutic blockade of the PD-1/PD-L1 axis using monoclonal antibodies has emerged as a powerful strategy for restoring T cell-mediated immune responses in various malignancies. Recent findings have identified tumor-infiltrating DCs and macrophages as critical sources of PD-L1 expression within the TME, underscoring their critical roles in shaping immune suppression and determining the efficacy of ICB [18]. PD-L1⁺ DCs have been shown to limit antigen-specific T cell responses and to evade cytotoxic killing by CD8⁺ T cells through PD-L1-mediated protection [19]. Traditionally, TAM-expressed PD-L1 has been associated with immune suppression, where it contributes to the inhibition of effector T cells and promotes a tolerogenic TME [20]. However, recent research suggests that PD-L1^**+**^ TAMs can also have immunostimulatory effects [21]. Beyond myeloid cells, regulatory B cells that express high levels of PD-L1 inhibit T-cell anti-tumor response via interleukin-10 (IL-10) signal in breast cancer [22]. PD-L1^**+**^ B cells have also been reported to markedly decrease both the proportion and number of follicular helper T cells, thereby suppressing inflammation [23]. Interestingly, recent studies have identified PD-L1⁺ CD8⁺ T cells in the TME of lung cancer, which paradoxically exhibit regulatory functions. These cells inhibit the proliferation and cytotoxic abilities of CD8^+^ T cells and are identified as a negative prognostic marker in patients with lung cancer [24, 25]. Collectively, these findings highlight the complex landscape of PD-L1 expression across both innate and adaptive immune cells within the tumor milieu. A deeper understanding of PD-L1⁺ cell populations will be essential for refining immunotherapeutic strategies and identifying predictive biomarkers for treatment response.

**Fig. 1:**
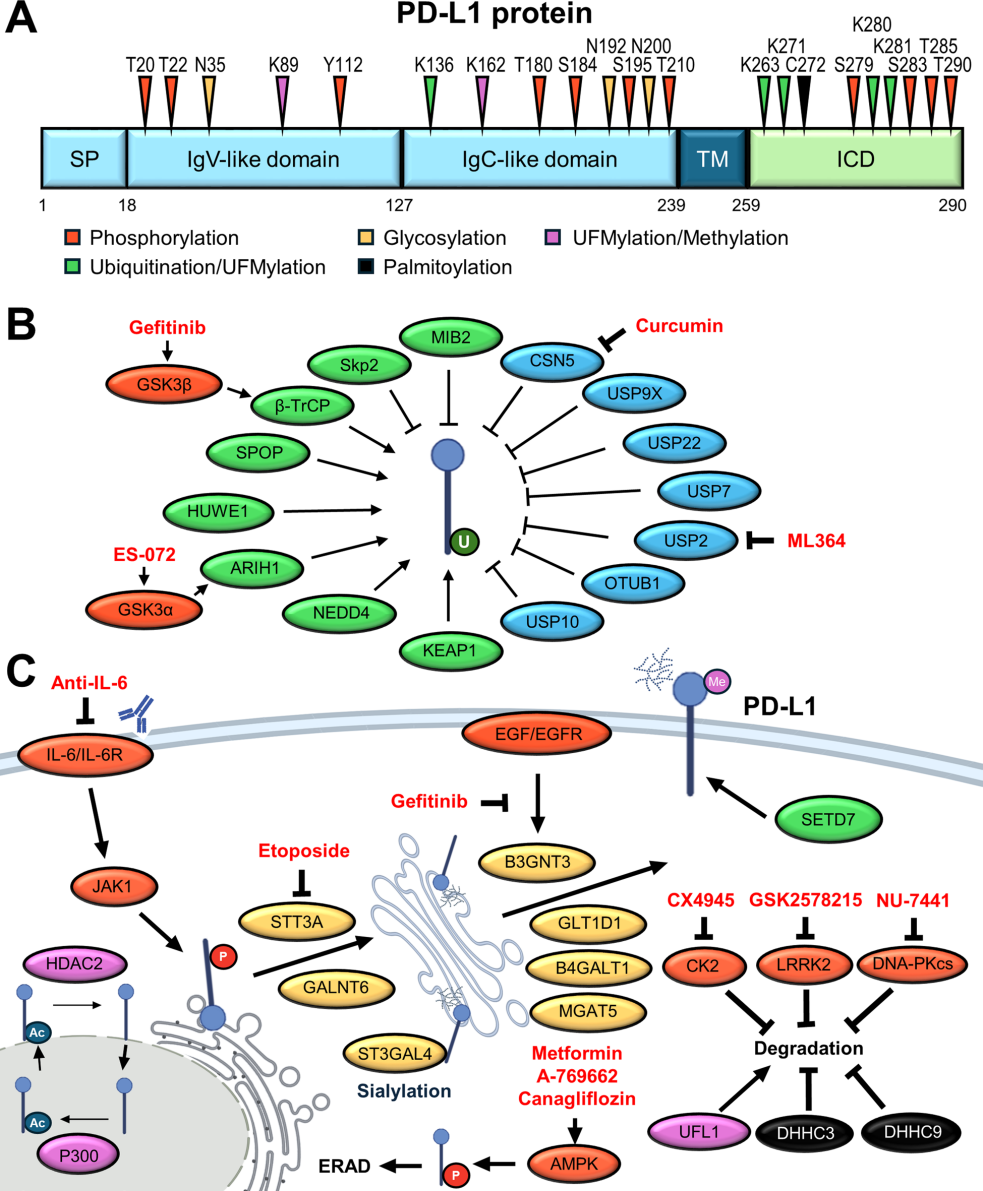
Post-translational modifications (PTMs) of PD-L1 regulate its localization, stability, and immune function. **A** Key serine, threonine, tyrosine, lysine, and cysteine residues of PD-L1 are modified by phosphorylation, ubiquitination, glycosylation, palmitoylation, UFMylation, acetylation, SUMOylation, methylation, and ISGylation. **B** The regulatory balance between ubiquitination, mediated by E3 ubiquitin ligases (green), and deubiquitination, mediated by deubiquitinases (blue), shapes PD-L1 expression and tumor immune evasion. **C** PTMs influence the intracellular trafficking routes, membrane presentation, and stability of PD-L1. Several glycosyltransferases and regulators, including B3GNT3, STT3A, GLT1D1, and B4GALT1, catalyze N-glycosylation, which stabilizes PD-L1. GALNT6 and MGAT5 further modify PD-L1 to prevent degradation. Phosphorylation by GSK3β or AMPK promotes PD-L1 ubiquitination and proteasomal or ER-associated degradation (ERAD), while CK2 and LRRK2 phosphorylation stabilize PD-L1 by disrupting E3 ligase binding. Several small-molecule inhibitors (red) interfere with these PTM regulators, thereby altering PD-L1 stability, and offer promising therapeutic targets for improving immunotherapy efficacy

PTMs, including phosphorylation, ubiquitination, deubiquitination, glycosylation, palmitoylation, UFMylation, acetylation, SUMOylation, methylation, and ISGylation of PD-L1, contribute to microenvironmental remodeling, promoting immunosuppressive niches, facilitating tumor progression, and modulating therapeutic responsiveness to immunotherapies (Fig. 1).

#### Phosphorylation

Phosphorylation regulates the stability, subcellular localization, and function of PD-L1 protein. Glycogen synthase kinase 3β (GSK3β)-mediated phosphorylation at T180 and S184, which promotes β-transducin repeat containing E3 ubiquitin protein ligase (β-TrCP)-mediated ubiquitination and subsequent proteasomal degradation [26]. Additionally, GSK3α phosphorylates the intracellular S279 and S283 residues of PD-L1, leading to Ariadne-1 homolog 1 (ARIH1)-mediated ubiquitination and proteasome-mediated degradation [27]. AMP-activated protein kinase (AMPK) phosphorylates PD-L1 at S283, which disrupts its interaction with CMTM4, a critical factor for recycling PD-L1 back to the plasma membrane [28]. Another report suggests that AMPK phosphorylates PD-L1 at the S195 site, blocking its endoplasmic reticulum (ER)-to-Golgi translocation and leading to the degradation of PD-L1 by the ER-associated degradation (ERAD) system [29]. These findings highlight how metabolic stress signals modulate checkpoint activity. Pro-inflammatory cytokine signaling also contributes to the phosphorylation of PD-L1. IL-6-activated Janus Kinase 1 (JAK1) phosphorylates PD-L1 at Y112, which recruits oligosaccharyltransferase complex catalytic subunit A (STT3A), linking phosphorylation to glycosylation-dependent stabilization [30]. Conversely, other protein kinases protect PD-L1 from degradation. Casein kinase 2 (CK2) phosphorylates PD-L1 at T285 and T290, which disrupts its binding to speckle-type POZ protein (SPOP), a component of the Cullin3 (CUL3)-RING E3 ubiquitin ligase complex, thereby preventing CUL3-mediated proteasomal degradation [31]. Similarly, leucine-rich repeat kinase 2 (LRRK2) phosphorylates PD-L1 at T210 within the ER, inhibiting ubiquitin-mediated degradation and promoting PD-L1 stability [32]. Furthermore, DNA-dependent protein kinase catalytic subunit (DNA-PKcs) phosphorylates PD-L1 at T20 and T22 to enhance PD-L1 stability, although the precise downstream mechanisms remain to be fully elucidated [33].

#### Ubiquitination

PD-L1 protein stability is tightly regulated by ubiquitination under various physiological and pathological conditions. PD-L1 contains five lysine residues (K263, K270, K271, K280, and K281) in the intracellular domain that serve as key ubiquitination sites. β-TrCP promotes the proteasomal degradation of PD-L1 in a GSK3β-mediated phosphorylation-dependent manner. Notably, N192, N200, and N219 residues are critical for GSK3β binding; however, epidermal growth factor (EGF)-induced glycosylation at these sites antagonizes this interaction, thereby stabilizing PD-L1 in breast cancer [26]. Zhang et al. revealed that PD-L1 expression fluctuates during the cell cycle, peaking during the M/early G1 phases and sharply decreasing in late G1/S phases, which is regulated by the E3 ligase CUL3-SPOP complex. Interestingly, SPOP stability is controlled by cyclin D–cyclin-dependent kinase 4 (CDK4)-mediated phosphorylation, which triggers its degradation through the anaphase-promoting complex/Cdc20 homolog 1 pathway [34]. Alcohol-induced aldehyde dehydrogenase 2 (ALDH2) activity stabilizes PD-L1 by physically interacting with the intracellular domain of PD-L1 and inhibiting SPOP-mediated proteasome degradation [35]. In addition, the antiviral sensor retinoic acid-inducible gene I (RIG-I) unexpectedly competes with SPOP for binding to PD-L1, thereby protecting it from ubiquitination and degradation [36]. Likewise, the sodium-glucose cotransporter 2 (SGLT2) has been shown to co-localize with PD-L1 at the plasma membrane and recycling endosomes, preventing SPOP-mediated degradation [37].

In breast cancer, HECT, UBA, and WWE domain protein 1 (HUWE1) ubiquitinates non-glycosylated PD-L1 at K281 and undergoes ERAD [38]. In non-small cell lung cancer (NSCLC), epidermal growth factor receptor (EGFR) inhibition activates GSK3α-mediated phosphorylation of PD-L1, which facilitates ARIH1-mediated ubiquitination at K271 and K281, promoting proteasomal degradation [27]. In bladder cancer, the activation of fibroblast growth factor receptor 3 induces NEDD4-mediated K48-linked polyubiquitination of PD-L1 [39]. Furthermore, kelch-like ECH-associated protein 1 (KEAP1) has recently emerged as a PD-L1 E3 ligase that promotes K48-linked polyubiquitination and degradation of PD-L1 in NSCLC [40]. Beyond the host cell context, the host–microbiota interaction also affects PD-L1 stability: *Fusobacterium nucleatum* suppresses ubiquitination-mediated degradation via IFIT1, resulting in enhanced PD-L1 expression [41]. Additional E3 ligases, including Ring finger protein 125 [42], CUL4-DDB1 ubiquitin E3 ligase complex [43], ubiquitin-editing enzyme A20 [44], STUB1 (also known as CHIP) [45], and membrane-associated RING-CH 8 [46], have also been implicated in PD-L1 ubiquitin-mediated degradation.

In addition to ubiquitination that targets PD-L1 for degradation, non-proteolytic ubiquitination also plays a role in PD-L1 stability. Mind bomb homolog 2 (MIB2) catalyzes K63-linked ubiquitination at K136, located in the extracellular domain, promoting PD-L1 trafficking through Ras-associated binding protein 8-mediated exocytosis from the trans-Golgi network to the plasma membrane [47]. In addition, F-box protein S-phase kinase-associated protein 2 (Skp2) mediates K63-linked polyubiquitination at K136 and K280, and recruits liver kinase B1 (LKB1), further stabilizing PD-L1 at the cell surface [48].

#### Deubiquitination

Counterbalancing the E3 ligase activities, multiple deubiquitinases (DUBs) have been identified that remove polyubiquitin chains, thereby preventing PD-L1 degradation. COP9 signalosome 5 (CSN5) was the first identified DUB for PD-L1. The pro-inflammatory cytokine TNF-α, secreted by macrophages, is upregulated in cancer cells, contributing to inflammation-driven immune evasion. CSN5 removes K48-linked ubiquitin chains to stabilize PD-L1 [49]. Ubiquitin-specific peptidase 9, X-linked (USP9X) deubiquitinates and stabilizes PD‐L1 in oral cancer cells [50]. USP22 interacts with the PD-L1 intracellular domain to remove multiple ubiquitin linkages, including K6, K11, K27, K29, K33, and K63, thereby enhancing PD-L1 stability in NSCLC cells. Notably, USP22 also stabilizes CSN5, thereby coordinately regulating PD-L1 [51]. USP7 directly interacts with PD-L1 to enhance its stability in gastric cancer [52]. USP2 removes K48-linked polyubiquitin chains from K263, K271, K280, and K281 to maintain PD-L1 expression in colorectal cancer. Depletion of USP2 accelerates ERAD-dependent degradation of PD-L1 [53]. The OTU family of cysteine proteases is also involved in regulating the stability of PD-L1. OTUB1 stabilizes PD-L1, especially in the ER, by cleaving K48-linked poly-ubiquitin chains and protects PD-L1 from the ERAD pathway in breast cancer [54]. Most recently, NDR1, an adaptor protein, was shown to facilitate the nuclear translocation of USP10, stabilizing both cytosolic and nuclear pools of PD-L1 in prostate cancer [55]. In addition, USP24 stabilizes PD-L1 in gefitinib-resistant lung cancer cells [56].

#### Glycosylation

Glycosylation is one of the most crucial PTMs regulating PD-L1 protein stability, cell-surface localization, ligand interaction, and ultimately its immunosuppressive function. The extracellular domain of PD-L1 contains four conserved N-linked glycosylation sites (N35, N192, N200, and N219), which are crucial for its structural integrity and function. Several glycosyltransferases and related factors have been identified to modulate the site-specific glycosylation of PD-L1. Beta-1,3-N-acetylglucosaminyltransferase 3 (B3GNT3) catalyzes the addition of poly-N-acetyllactosamine (LacNAc) chains at the N192 and N200 sites, which are required for optimal PD-L1/PD-1 interaction and subsequent immune checkpoint signaling [57]. STT3A, an ER-associated N-glycosyltransferase, is responsible for promoting global N-glycosylation and stabilizing PD-L1 in breast cancer cells [58]. Interestingly, transmembrane and ubiquitin-like domain-containing protein 1 (TMUB1) has been shown to recruit STT3A, enhancing PD-L1 N-glycosylation and stability, thereby highlighting an additional layer of regulation at the ER membrane [38]. Moreover, STT3A expression itself is transcriptionally regulated by the Wnt/β-catenin pathway, the TGF-β1/c-Jun signaling pathway, and the protease-activated receptor 2 signaling pathway [59–61]. Glycosyltransferase 1 domain-containing 1 (GLT1D1) facilitates the transfer of N-linked glycans to PD-L1, promoting tumor immune evasion in B-cell non-Hodgkin’s lymphoma [62]. Similarly, β-1,4-galactosyltransferase 1 (B4GALT1) mediates the biosynthesis of N-glycans at the N192 and N200 sites, which prevents PD-L1 degradation in lung adenocarcinoma [63]. In pancreatic ductal adenocarcinoma (PDAC), GALNT6 was found to glycosylate PD-L1, thereby preventing its proteasomal degradation and enhancing its immune checkpoint activity [64]. Beyond classical glycosyltransferases, mannoside acetyl-glucosaminyltransferase 5 (MGAT5) generates β1,6-branched N-glycans at the N35 and N200 sites, which modulate PD-L1’s binding affinity for PD-1 and interfere with the cytotoxicity of chimeric antigen receptor (CAR) T cells [65]. Additionally, the translocon subunit SEC61G facilitates the co-translational translocation of nascent PD-L1 into the ER lumen, promoting its proper folding and glycosylation [66]. Intriguingly, metabolic factors also contribute to PD-L1 glycosylation. Environmental lactate upregulates PD-L1 N-glycosylation through the monocarboxylate transporter 4 and the WNT pathway [67].

Recently, additional glycan modifications such as sialylation and sulfation have been reported to modulate PD-L1 activity. For instance, ST3 beta-galactoside alpha-2,3-sialyltransferase 4 (ST3GAL4) catalyzes the addition of sialic acid residues to the terminal ends of N-glycan chains, thereby promoting the sialylation of PD-L1 in endothelial cells. This modification facilitates PD-L1 interaction with CD169, which in turn enhances monocyte adhesion to endothelial cells and contributes to the regulation of tumor metastasis [68, 69]. Moreover, carbohydrate sulfotransferase 8 (CHST8) mediates sulfation of N-glycans at N35, N192, and N200 of PD-L1. Elevated CHST8 expression in tumor cells has been associated with suppressed T-cell activation and poor prognosis in patients undergoing pembrolizumab therapy. However, the precise functional consequences of PD-L1 sulfation remain to be elucidated (70).

#### Palmitoylation

Palmitoylation of PD-L1 by zinc finger DHHC-type palmitoyltransferase 3 (DHHC3) at C272 has been shown to block PD-L1 mono-ubiquitination, thereby inhibiting ESCRT-mediated multivesicular body sorting and lysosomal degradation in various cancer types [71]. In the same year, another report revealed that DHHC9-mediated palmitoylation increases PD-L1 protein stability in breast cancer cells [72]. More recently, it was found that palmitoylation of PD-L1 not only affects its turnover but also modulates its affinity for lipid rafts, alters its membrane localization, and impacts its membrane orientation, thereby enhancing its binding capability to PD-1 on T cells [73]. These reports suggest targeting the PD-L1 palmitoylation process or its mediating enzymes may represent a promising strategy for cancer immunotherapy.

#### UFMylation

UFMylation is an emerging and less-characterized PTM involving the covalent attachment of ubiquitin-fold modifier 1 (UFM1) to lysine residues on substrate proteins via an enzymatic cascade comprising E1, E2, and E3 (UFL1) enzymes. UFM1-specific ligase 1 (UFL1), the UFM1 E3 ligase, promotes UFMylation of PD-L1, which synergizes with ubiquitination to promote PD-L1 degradation. Conversely, inhibition of the de-UFMylation enzyme UFSP2 results in a reduction of PD-L1 stability and an improvement in the efficacy of anti-PD-1 immunotherapy in a breast cancer mouse model [74].

#### Acetylation

Acetylation is a PTM in which lysine residues of protein are modified with the acetyl group by acetyltransferases using acetyl-Coenzyme A as the donor molecule [15]. Acetylation of non-histone proteins has emerged as a key regulatory mechanism that can modulate protein stability and subcellular localization [75]. A recent study reveals that nuclear localization of PD-L1 is regulated by acetylation at K263 by the acetyltransferase p300, which interrupts its nuclear translocation. Conversely, deacetylation by histone deacetylase 2 (HDAC2) promotes PD-L1 to undergo clathrin-dependent endocytosis, traffic via the cytoskeleton, and subsequently nuclear import. Once translocated into the nucleus, nuclear PD-L1 can bind directly to DNA and modulate the expression of immune-response-related genes, ultimately shaping the TME [76].

#### SUMOylation

Small ubiquitin-like modifier modification (SUMOylation) is a process in which a member of the SUMO protein family is covalently attached to specific lysine residues on substrate proteins. SUMOylation regulates various cellular processes, including protein stability, subcellular localization, and protein–protein interactions [16]. Huang et al. demonstrate that tripartite motif containing 28 (TRIM28) stabilizes PD-L1 by inhibiting PD-L1 ubiquitin-mediated proteasomal degradation and promoting PD-L1 SUMOylation. Notably, TRIM28 has a dual function that not only targets proteins for SUMOylation but also promotes ubiquitination. TRIM28 also facilitates K63 polyubiquitination of TANK-binding kinase 1 (TBK1), which augments TBK1-mediated downstream pathways such as IRF1 and mTOR, resulting in enhanced PD-L1 transcription [77].

#### Methylation

Methylation of non-histone proteins at lysine or arginine residues is a critical PTM that modulates many cellular processes, including protein–protein and protein-DNA interactions, protein stability, and subcellular localization. PD-L1 protein has been identified to contain six mono-methylation sites (K75, K89, K105, R113, K162, and R212). Among these residues, K162 is critical for regulating PD-L1 methylation and its interaction with the PD-1 receptor but does not affect PD-L1 expression levels, protein stability, or membrane translocation. Mechanistically, PD-L1 is methylated by the histone-lysine N-methyltransferase SET domain containing 7 (SETD7), which interrupts its binding to PD-1 and enhances T cell activity. At the same time, lysine-specific HDAC2 mediates its demethylation. Clinically, PD-L1 hypermethylation in NSCLC patients correlates with resistance to PD-1/PD-L1 blockade. Beyond the current use of PD-L1 IHC scoring in clinical trials (positivity defined as ≥ 50% of tumor cells), objective response rates remain below 50%. This finding provides a more reliable biomarker for predicting sensitivity to anti-PD/PD-L1 therapy [78].

#### ISGylation

ISGylation is a PTM similar to ubiquitination, in which the ubiquitin-like protein interferon-stimulated gene 15 (ISG15) is covalently attached to target proteins on lysine residues. ISG15, a member of the ubiquitin-like (UBL) protein family, consists of two UBL domains and can modify specific proteins through an enzymatic cascade analogous to ubiquitination. This process is catalyzed sequentially by the E1-activating enzyme, the E2-conjugating enzyme, and various E3 ligases, including HECT and RLD domain-containing E3 ubiquitin protein ligase 5, ARIH1, and TRIM25. Functionally, ISGylation regulates protein stability by competing with or promoting protein degradation through the ubiquitin–proteasome system or the lysosomal-associated pathway, thereby serving as an important modulator of cellular homeostasis and immune responses [17]. Qu and associates demonstrated that ISG15 enhanced K48-linked ubiquitination modification of PD-L1, thus increasing the degradation rate of PD-L1 [79].

#### Ectodomain shedding

Ectodomain shedding is a PTM in which membrane-bound proteins undergo proteolytic cleavage, leading to the release of their extracellular domains [80]. For PD-L1, a disintegrin and metalloproteinase 10 (ADAM10) and ADAM17 cleave the PD-L1 ectodomain from tumor cells to generate soluble PD-L1 (sPD-L1) and modulate the TME. Tumor-derived sPD-L1 has been shown to induce apoptosis of activated human CD8⁺ T cells, thereby contributing to immune evasion [81]. However, during chronic inflammation, PD-L1 is cleaved by matrix metalloproteinase 13 (MMP-13), and consequently, it limits PD-L1-mediated immunosuppressive activity in fibroblasts. Disruption of MMP13-dependent cleavage of PD-L1 contributes to the exacerbation of inflammation-associated severe tissue damage [82]. Hira-Miyazawa et al. further confirm the activity of MMP-13 and -7 in PD-L1 ectodomain cleavage in head and neck squamous cell carcinoma (HNSCC) [83]. Clinically, elevated sPD-L1 levels are significantly associated with poor overall survival (OS) and progression-free survival (PFS) in cancer patients, including those with lung, gastric, and colorectal cancer receiving immune checkpoint inhibitors [84–87], suggesting that sPD-L1 could be a useful predictive biomarker for immunotherapy response.

### PD-1

**Fig. 2:**
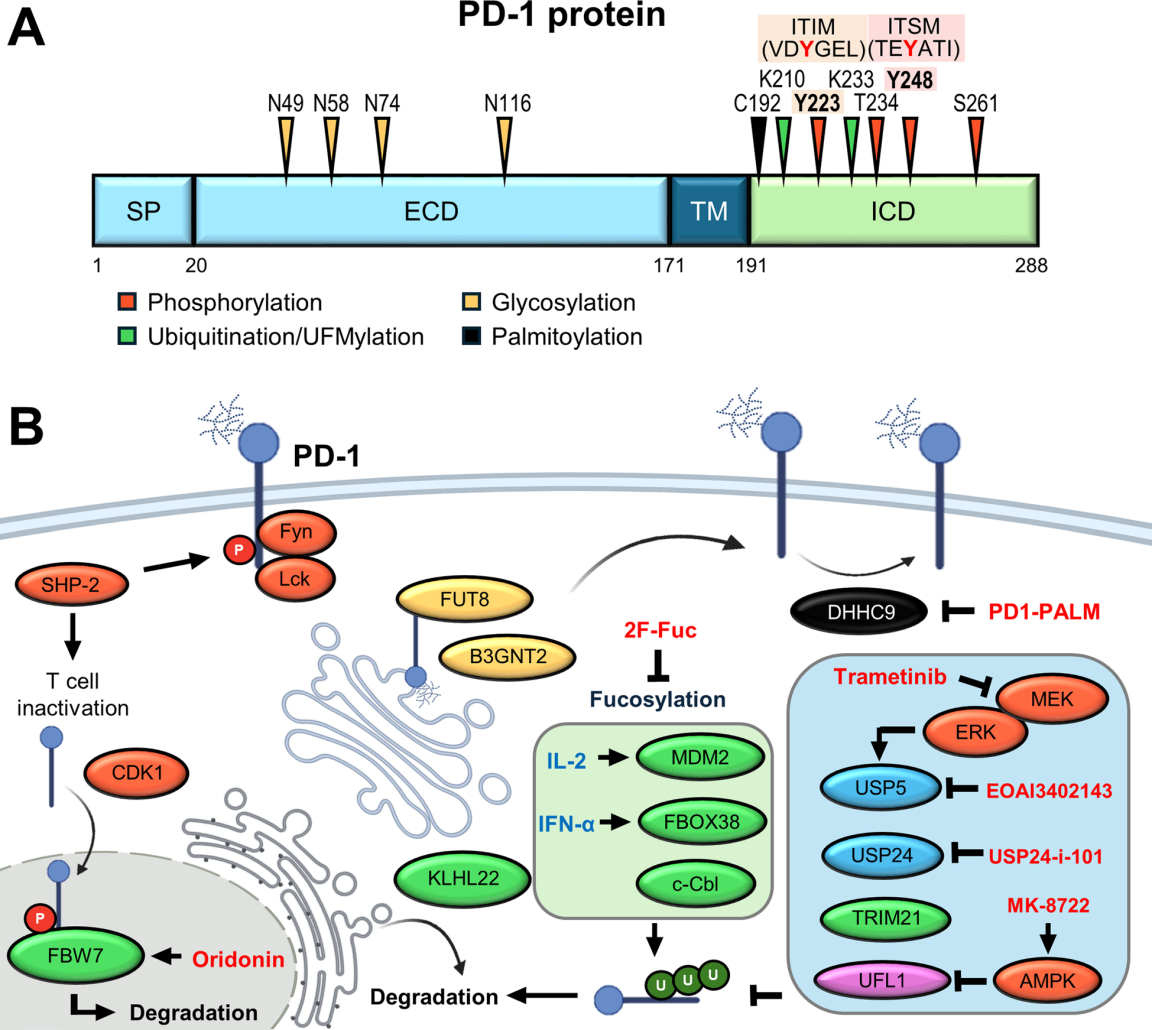
PTMs regulate PD-1 stability and immunosuppressive signaling. **A** Key serine, threonine, tyrosine, lysine, and cysteine residues of PD-L1 are modified through phosphorylation, ubiquitination, glycosylation, and palmitoylation. **B** Schematic of PD-1 intracellular trafficking and degradation regulated by PTM. Deubiquitination by USP234 or Phosphorylation by CDK1 and ERK promotes nuclear translocation and stabilization via USP5 binding, while cytoplasmic PD-1 is targeted for degradation by E3 ligases such as FBXO38, KLHL22, and c-Cbl. UFMylation by UFL1 stabilizes PD-1, whereas AMPK-dependent UFL1 phosphorylation inhibits this process and promotes degradation. Inhibitors shown in red letters antagonize the activity of enzymes that regulate PD-1 stability, offering potential strategies to reinvigorate T cell function and enhance responses to ICB

Programmed cell death-1 (PD-1) is a type I transmembrane glycoprotein with a structure consisting of an extracellular domain, a transmembrane region, and a cytoplasmic tail. PD-1 is predominantly expressed on immune cells, including T cells, B cells, NK cells, macrophages, and DCs. Upon engagement with its ligands, PD-L1 (CD274, B7-H1) and PD-L2 (CD273, B7-DC) expressed on tumor cells or APCs, PD-1 transduces inhibitory signals that dampen cytotoxic CD8^+^ T cell activation and contribute to tumor immune evasion in cancer [2]. In Treg, PD-1^hi^ Tregs exhibit a dysfunctional and exhausted phenotype [88]. PD-1 negatively regulates Treg cells by downregulating the STAT5/CD30 axis, thereby limiting their suppressive function [89]. Notably, PD-1 blockade enhances the proliferation and suppressive function of Tregs [90]. In addition to T cells, the upregulation of PD-1 expression in tumor-associated macrophages impairs their phagocytic capability against tumor cells, while PD-1 blockade restores macrophage phagocytosis, thereby reducing tumor growth [91]. Moreover, PD-1-expressing tumor-infiltrating DCs suppress T cell activity, characterized by a reduction in IL-2 and IFN-γ secretion, as well as T cell proliferation [92]. Growing evidence suggests that PD-1 has a suppressive function in NK cells. PD-1/PD-L1 exerts an inhibitory effect by inactivating the PI3K/AKT signaling pathway and promotes apoptosis in NK cells, which is correlated with a poor prognosis in various digestive cancers [93, 94]. Elevated PD-1 expression in protumorigenic regulatory B cells suppresses tumor-specific T-cell immunity by secreting immunosuppressive cytokine IL-10 [95]. This evidence suggests PD-1 expression not only regulates T cell immunity but also modulates the whole immune system within the TME (Fig. 2).

#### Phosphorylation

Phosphorylation serves as an initial trigger for PD-1 signal transduction. The cytoplasmic tail of PD-1 contains two tyrosine-based structural motifs, an immunoreceptor tyrosine-based inhibitory motif (ITIM) and an immunoreceptor tyrosine-based switch motif (ITSM). Upon PD-L1 ligation, PD-1 is phosphorylated by the Src-family kinases, such as tyrosine-protein kinase Fyn and lymphocyte-specific protein tyrosine kinase (Lck) at Y223 (ITIM) and Y248 (ITSM), thereby recruiting *Src* homology region 2 domain-containing phosphatase-2 (SHP-2) to dephosphorylate proximal signaling molecules downstream of CD28 and the T cell receptor (TCR), such as RAS/ERK, PI3K/AKT, and calcium-mediated signaling [96]. Although SHP-2 may interact with both ITIM and ITSM of PD-1, phosphorylation site Y248 of PD-1 is crucial for PD-1 inhibitory function [97]. Based on these findings, an antibody specific for phosphorylated PD-1 at Y248 was generated to detect PD-1-mediated inhibitory signaling. Upregulated phosphorylation of PD-1 at Y248 is observed in tumor-infiltrating lymphocytes (TILs), especially in PD-1^+^TIM-3^+^ exhausted TILs. Notably, PD-1 phospho-signal was decreased in PD-1 blockade-treated Hodgkin lymphoma patients [98]. Recent evidence also highlights non-canonical phosphorylation events that regulate PD-1 trafficking and protein interactions. For instance, CDK1-mediated phosphorylation at S261 facilitates PD-1 nuclear translocation, thereby promoting its interaction with nuclear-localized E3 ligase F-box and WD repeat domain-containing 7 (FBW7), which triggers PD-1 degradation [99]. Additionally, ERK-mediated phosphorylation at T234 enhances the binding of PD-1 to the deubiquitinase USP5, protecting PD-1 from degradation and sustaining its expression in TILs [100].

#### Ubiquitination

Ubiquitination plays a crucial role in regulating the stability, activity, and subcellular localization of the PD-1 protein. Multiple E3 ligases have been identified as regulators of PD-1 turnover in various cellular compartments. For example, F-box only protein 38 (FBXO38) processes K48-linked polyubiquitination of PD-1 at K233 after PD-1 internalization, targeting it for proteasomal degradation [101]. Kelch-like family member 22 (KLHL22) induces PD-1 ubiquitination at K210 and K233 in T cells to degrade incompletely glycosylated PD-1 before being transported to the plasma membrane. 5-Fluorouracil (5-FU) chemotherapy downregulates KLHL22 expression, leading to PD-1 accumulation and impaired anti-tumor immunity, which suggests chemotherapy-induced immunosuppression [102]. Casitas B lineage lymphoma (c-Cbl) destabilizes PD-1 at the plasma membrane in both T cells and macrophages, contributing to enhanced anti-tumor responses [103]. FBW7 has been shown to regulate K48-linked polyubiquitination at K233, thereby mediating degradation in the nucleus. CDK1-mediated phosphorylation primes PD-1 protein for nuclear translocation and binding to FBW7 [99]. Mouse double minute 2 (MDM2) destabilizes PD-1 glycosidase, N-glycanase 1, as well as directly promoting K48-linked polyubiquitination of deglycosylated PD-1 at K78 [104]. Recently, TRIM21 has been reported to catalyze the K63-linked ubiquitination at K233, which stabilizes PD-1 by antagonizing K48-linked ubiquitination and degradation. TRIM21 deficiency in CAR-T cells reduces PD-1 expression and augments anti-tumor activity [105].

#### Deubiquitination

Counteracting ubiquitin ligases, DUBs remove ubiquitin chains from modified proteins, thereby modulating protein stability, activity, and localization (100). A study revealed USP5 as the first PD-1 DUB. It interacts with PD-1 in tumor-infiltrating T cells and protects PD-1 from ubiquitin-mediated degradation. ERK-mediated phosphorylation at T234 promotes interaction of PD-1 with USP5 [100]. USP24, a more recently characterized DUB, either directly removes K48-linked polyubiquitin or counteracts E3 ligase c-Cbl binding, leading to PD-1 stabilization. IL-6/STAT3 signal enhances USP24 activity in T cells, which correlates with high PD-1 expression and poor immunotherapy response in NSCLC patients [106]. Together, the interplay between E3 ligases and DUBs serves as a critical checkpoint for controlling PD-1 abundance and T cell anti-tumor immunity.

#### Glycosylation

PD-1 is a highly glycosylated protein, and this PTM is critical for its protein folding, stability, membrane trafficking, ligand-receptor binding, and regulation of T cell activity [107]. A previous report demonstrates that upon TCR activation, the glycosyltransferase B3GNT2 is upregulated to initiate the synthesis of poly-LacNAc at asparagine 49 (N49), N58, N74, and N116 sites. Inhibition of PD-1 N-glycosylation leads to the accumulation of ubiquitination on PD-1 and degradation, thereby reactivating T cell-mediated immunity [108]. Glycosylation of PD-1, especially at the N58 site, promotes PD-1 protein stability and reinforces binding affinity for ligand PD-L1 in TILs [108]. Moreover, this site influences the binding efficacy of monoclonal antibody camrelizumab [109], suggesting a critical role of glycosylation in the development of anti-PD-1 immunotherapies. Another study demonstrates that upon T cell activation, PD-1 undergoes core-fucosylation at N49 and N74 in an alpha 1,6-fucosyltransferase FUT8-dependent manner [110]. Moreover, T153, S157, S159, and T168 are modified by sialylated mucin-type O-glycan with core 1- and core 2-based structures [111].

#### Palmitoylation

Palmitoylation is the reversible attachment of palmitic acid to cysteine residues via thioester bonds, impacting a dynamic phenomenon that influences a large number of cellular properties of proteins, ranging from protein stability, membrane domain organization, protein trafficking, and protein function [112]. PD-1 is palmitoylated at C192 by the palmitoyltransferase DHHC9. This PTM facilitates PD-1 association with RAB11, promoting its recycling endosome localization and protecting it from lysosomal degradation. Notably, palmitoylation of tumor intrinsic PD-1 activates mTOR signaling and promotes tumor cell proliferation [113].

#### UFMylation

In contrast to the role of UFMylation in destabilizing PD-L1 [71], UFL1, the UFM1 E3 ligase, promotes the stability of PD-1 through antagonizing K48-linked ubiquitination, thereby preventing PD-1 from undergoing proteasomal degradation. Notably, the activity of UFL1 is tightly regulated by the cellular metabolic state. The energy sensor AMPK phosphorylates UFL1 at T536, facilitating its interaction with the adaptor protein 14-3-3. This phosphorylation-dependent binding inhibits the association between UFL1 and PD-1, thereby reducing PD-1 UFMylation and promoting its degradation [114].

### TIM-3

T cell immunoglobulin and mucin-domain containing-3 (TIM-3), encoded by *HAVCR2*, is an inhibitory checkpoint expressed not only on dysfunctional or exhausted T cells but also on DCs and macrophages within the TME [4, 115]. Originally identified as a ligand for Galectin-9, TIM-3 negatively regulates TCR signaling and cytokine production, thereby contributing to immune tolerance in cancer [116, 117]. More recent studies have shown that Galectin-9 can also interact with PD-1, functioning cooperatively with TIM-3 to induce T cell apoptosis, thus highlighting Galectin-9 as a key immunoregulatory ligand and potential therapeutic target [118]. In addition to Galectin-9, carcinoembryonic antigen-related cell adhesion molecule 1 (CEACAM-1) has been identified as a functional ligand for TIM-3, and it plays a role in mediating immune suppression and promoting T cell exhaustion through heterodimeric interaction and co-signaling [119, 120].

While the immunoregulatory role of TIM-3 in T cells has been extensively studied, its expression and function in non-T cell compartments have garnered increasing attention [121]. In the myeloid compartment, TIM-3 restricts the activation of the cGAS-STING pathway in tumor-infiltrating DCs by limiting the uptake of extracellular DNA and by sequestering high-mobility group box 1 (HMGB1), an alarmin that facilitates nucleic acid-mediated innate immune sensing. This suppresses type I interferon responses and contributes to immune evasion [122, 123]. Recent studies further suggest that TIM-3 promotes DC maturation and enhances antigen processing and presentation, whereas its inhibition impairs DC differentiation and T cell activation [124].

In tumor-associated macrophages, TIM-3 expression is upregulated by TGF-β and correlates with an M2-like immunosuppressive phenotype [125, 126]. Intriguingly, glioma-intrinsic TIM-3 has been shown to regulate not only the malignant behaviors of glioma cells but also the recruitment and polarization of macrophages toward an anti-inflammatory and pro-tumorigenic state via a TIM-3/IL-6 signaling axis. Mechanistically, TIM-3 modulates IL-6 expression through activation of the NF-κB pathway [127]. Similar regulatory pathways are observed in NK cells, where TIM-3 expression is induced by TNF-α through NF-κB signaling, and is associated with reduced cytotoxic activity and IFN-γ production, particularly under chronic inflammatory or tumor conditions [128, 129].

These findings collectively underscore TIM-3 as a multifaceted immunoregulatory receptor that integrates diverse extracellular cues such as Galectin-9, CEACAM-1, and HMGB1, across both adaptive and innate immune compartments. This functional diversification, characterized by cell type-specific ligand usage and context-dependent signaling, warrants further investigation into its PTMs and therapeutic potential.

**Fig. 3:**
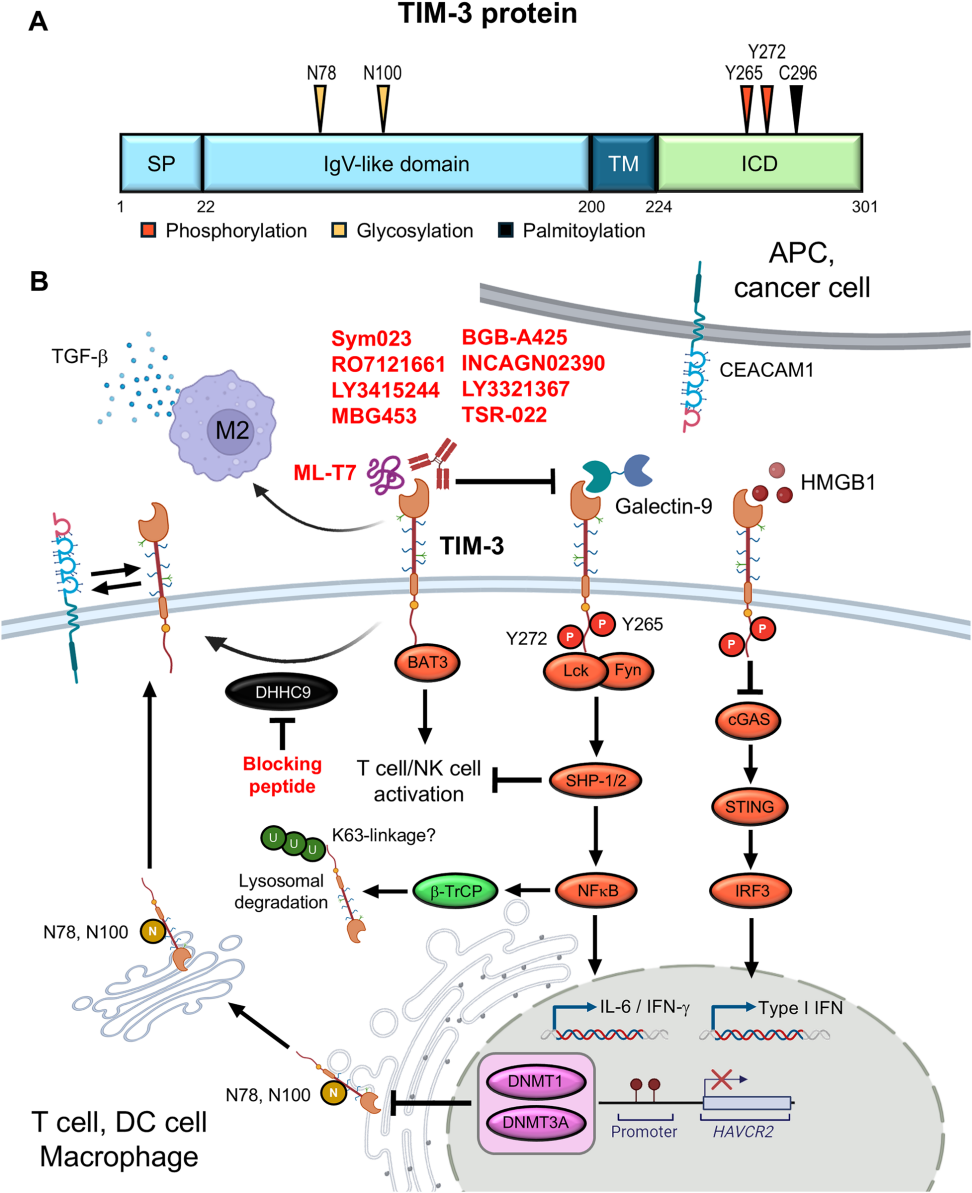
PTMs and ligand-induced signaling regulate TIM-3 expression and immunosuppressive activity in the TME. **A** Key Asparagine, tyrosine, and cysteine residues of TIM-3 are modified through phosphorylation, glycosylation, and palmitoylation. **B** Mechanistic overview of TIM-3 signaling across immune compartments. In T and NK cells, ligand engagement induces the phosphorylation of intracellular tyrosine residues, leading to BAT3 dissociation and the recruitment of SHP1/2, thereby suppressing effector function. In DCs, TIM-3 modulates cGAS-STING signaling by sequestering extracellular DNA and HMGB1, reducing type I IFN production. In macrophages, TGF-β-induced TIM-3 expression promotes M2 polarization through β-TrCP-mediated ubiquitination and NF-κB activation. DHHC9-catalyzed palmitoylation stabilizes membrane-bound TIM-3, while glycosylation ensures proper folding and surface expression. Epigenetic repression by DNMT1 and DNMT3A further regulates *HAVCR2* transcription. Multiple blocking antibodies and inhibitors (red) targeting TIM-3 or its regulatory enzymes are under development

PTMs critically regulate the stability, trafficking, and immunomodulatory activity of checkpoints [130]. For TIM-3, multiple PTMs have been identified that fine-tune its functions in T cells, DCs, and macrophages within the TME (Fig. 3).

#### Phosphorylation

In resting T cells, Y256 enables association with HLA-B-associated transcript 3 (Bat3), a scaffold that blocks inhibitory signaling [131, 132]. Upon ligand binding, Y256 becomes phosphorylated, resulting in Bat3 dissociation and subsequent recruitment of SH2 domain-containing kinases such as Fyn and Lck, leading to suppression of TCR signaling and cytokine production. Mutation at this site disrupts SHP-1/2 engagement and restores effector function [133]. Moreover, PTPN2 deficiency enhances TIM-3 signaling and exhaustion phenotypes, likely through dysregulated phospho-tyrosine signaling [134, 135]. A similar Bat3-dependent mechanism is also present in DCs, where TIM-3-Bat3 interaction restrains the acquisition of a tolerogenic phenotype. Loss of Bat3 in DCs drives unfolded protein response activation and steroidogenesis, suppressing T cell priming and accelerating tumor progression [136]. These findings suggest that Bat3-mediated control of TIM-3 may constitute a conserved regulatory axis in both adaptive and innate immune cells.

#### Ubiquitination

Although specific ubiquitination sites on TIM-3 remain uncharacterized, recent studies in hepatic macrophages have revealed a functional β-TrCP-dependent polyubiquitination mechanism. Elevated β-TrCP expression promotes TIM-3 ubiquitination and plasma membrane translocation, augmenting PI3K interaction and inhibiting mTOR signaling. This axis fosters M2 macrophage polarization and enhances TGF-β release, resulting in aggravated fibrosis in NASH models [137]. This study uncovers a previously underappreciated TIM-3-E3 ligase interaction in myeloid regulation, positioning ubiquitination as a potential immunomodulatory checkpoint in chronic inflammatory diseases.

#### Glycosylation

N-linked glycosylation, particularly at conserved asparagine residues (e.g., N78, N100) in the IgV domain, is essential for proper folding, ER exit, and extracellular interactions of TIM-3 [138]. Glycosylation of TIM-3 and PD-1 supports formation of a trimeric TIM-3/Galectin-9/PD-1 lattice on the immune synapse, where PD-1’s N116-linked glycan is crucial for Galectin-9 binding [118].

#### Methylation

TIM-3 expression is epigenetically regulated through both DNA methylation and histone modifications, particularly at the *HAVCR2* promoter. Hypomethylation of CpG islands in this region has been linked to elevated TIM-3 mRNA and protein expression in exhausted T cells and TAMs across multiple cancers [139]. In glioblastoma, high *MGMT* promoter methylation is associated with lower TIM-3 expression and improved survival, indicating immune regulation and enhanced chemotherapy sensitivity. TCGA data further reveal an inverse link between *HAVCR2* methylation and T cell dysfunction [140]. In cervical cancer, TIM-3 and its ligand, Galectin-9, are co-repressed by SUV39H1-mediated H3K9me3 and DNMT3A-dependent DNA methylation, resulting in impaired immune recognition and tumor progression. Pharmacological inhibition of DNA methyltransferases (e.g., 5-aza-dC) restores TIM-3 expression and partially rescues T cell function [141]. Moreover, overexpression of DNMT1 or DNMT3A suppresses TIM-3 expression, whereas promoter demethylation reverses this effect [142], highlighting DNA methylation as a dynamic and therapeutically targetable regulator of TIM-3, which can be a potential biomarker for predicting immunotherapy responsiveness.

#### Palmitoylation

Beyond phosphorylation, palmitoylation at C296 has emerged as a functionally critical lipid modification. This process, catalyzed by DHHC9, stabilizes TIM-3 on the cell membrane and facilitates its suppressive activity in exhausted T cells. Targeting DHHC9-mediated palmitoylation restores cytokine secretion and enhances effector function, suggesting a tractable vulnerability in exhausted lymphocytes [143].

#### Ectodomain shedding

TIM-3 undergoes ectodomain shedding mediated by ADAM10 and ADAM17, generating soluble TIM-3 (sTIM-3). The cleavage site of ADAM17 is located between E181 and D190 [144]. Functionally, sTIM-3 attenuates CD8⁺ T-cell responses and promotes terminal T-cell exhaustion through interaction with CEACAM-1 on T cells [145]. Myeloid cells have been identified as a major source of sTIM-3. Experimental study further indicates that sTIM-3 overexpression facilitates tumor progression and contributes to resistance against PD-1 blockade in various murine tumor models [146]. Clinically, serum sTIM-3 levels are markedly elevated across multiple cancer types and correlate with advanced disease stage [147]. In metastatic clear cell renal cell carcinoma (ccRCC) and NSCLC patients, high basal sTIM-3 levels are associated with poor OS following anti-PD-1 monotherapy [146, 148].

### TIGIT

T cell immunoreceptor with Ig and ITIM domains (TIGIT) is an inhibitory checkpoint initially identified on activated and exhausted CD8⁺ T cells, Tregs, and NK cells. It binds several nectin/nectin-like ligands, including PVR (CD155), PVRL2 (CD112), PVRL3 (Nectin-3, CD113), and PVRL4 (Nectin-4), all commonly expressed by tumor cells and APCs [18]. Functional studies have shown that Nectin‑3/4-TIGIT engagement attenuates CD8⁺ T cell proliferation and cytokine secretion, while also reducing NK cell cytotoxicity [149]. Similarly, the PVRL2-TIGIT interaction inhibits both T and NK cell effector functions, and combined blockade of TIGIT and PVRL2 significantly enhances antitumor responses in vivo [150]. The well-characterized PVR-TIGIT axis suppresses T cell activation by outcompeting costimulatory signaling (e.g., CD226) and directly dampens NK cell activity; disrupting this axis restores cytotoxicity and IFN-γ production [151].

Beyond its suppressive role in lymphocytes, TIGIT also exerts significant regulatory effects on myeloid cells, including macrophages and DCs, thereby contributing to TME remodeling. Recent studies have demonstrated that TIGIT engagement on DCs via its high-affinity ligand PVR reprograms cytokine output toward an IL-10^high^/IL-12^low^ immunosuppressive profile, which impairs effective T cell priming and fosters immune tolerance within the TME [152].

Moreover, TIGIT directly inhibits NK cell effector functions by limiting cytotoxic granule release and IFN-γ production. Blockade of TIGIT restores NK cytotoxicity in preclinical models of NSCLC and diffuse large B-cell lymphoma [153, 154]. Notably, TIGIT expression in tumors correlates with the presence of tolerogenic macrophages and immature DCs. Its ligands (e.g., CD155) are highly expressed on tumor and stromal cells, reinforcing immunosuppression and preventing effective T cell priming [155]. Taken together, these findings highlight the central role of TIGIT in immunoregulation by modulating cellular crosstalk and cytokine balance within the TME [156]. Through its interaction with multiple nectin-family ligands, TIGIT suppresses the effector functions of CD8⁺ T cells and NK cells, while simultaneously reprogramming DCs and macrophages into tolerogenic phenotypes. This dual regulatory function in both the lymphoid and myeloid compartments contributes to immune exclusion and facilitates tumor immune evasion.

**Fig. 4:**
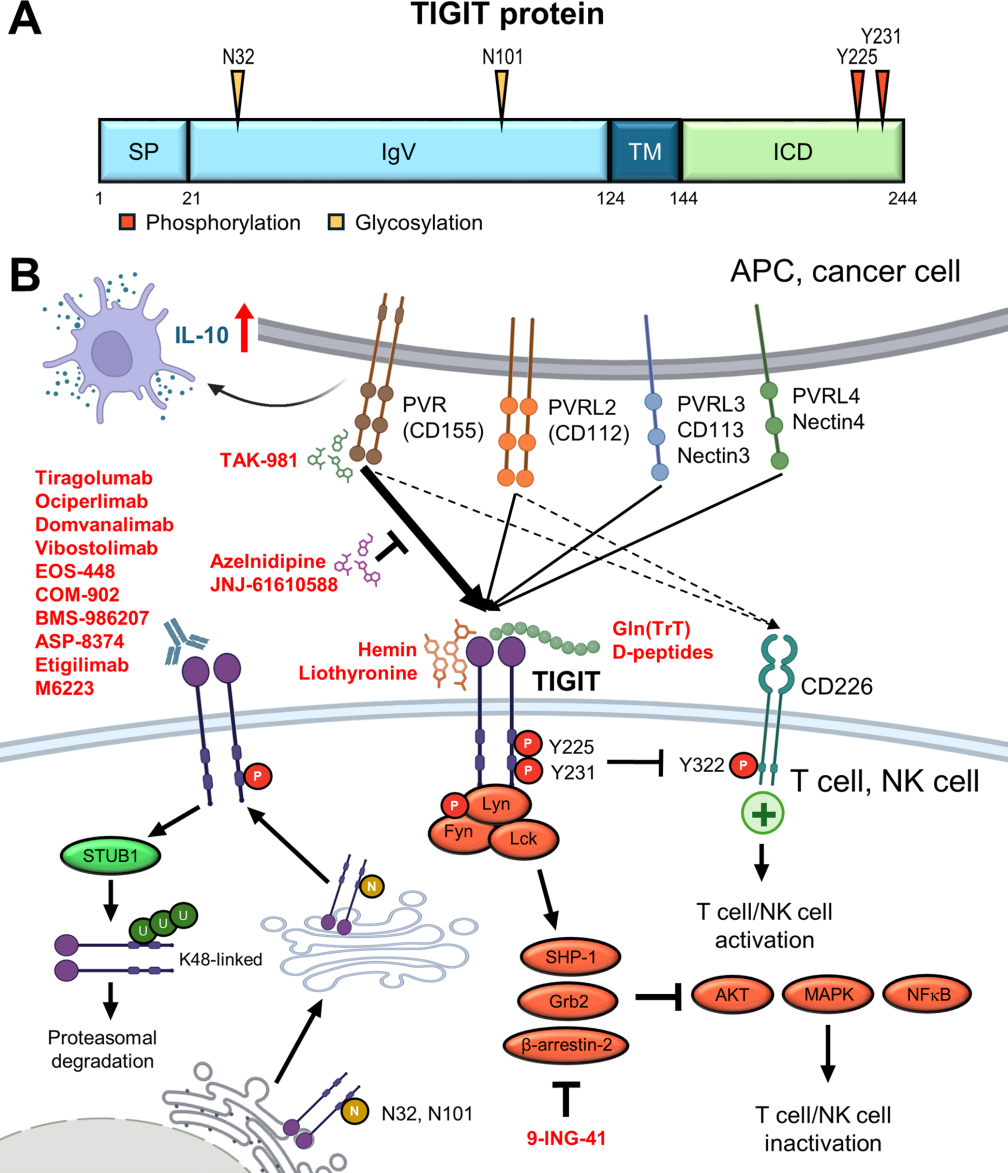
TIGIT checkpoint function is shaped by ligand-dependent signaling and PTMs that coordinate suppression across lymphoid and myeloid compartments. **A** Key Asparagine, tyrosine, and lysine residues of TIM-3 are modified through phosphorylation, glycosylation, and ubiquitination. **B** Mechanistic overview of TIGIT-mediated immune suppression. TIGIT binds multiple nectin-family ligands (CD155, CD112, CD113, Nectin-4) expressed by tumor cells and APCs, outcompeting CD226 and suppressing T/NK cell activity. Upon ligand engagement, TIGIT becomes phosphorylated by Src family kinases (Lck, Fyn), recruiting SHP1, Grb2, and β-arrestin-2 to inhibit downstream AKT/MAPK/NF-κB signaling. STUB1-mediated K48-linked ubiquitination facilitates proteasomal degradation of TIGIT. N-linked glycosylation promotes surface expression and ligand binding. TIGIT also reprograms DCs to secrete IL-10 and fosters tolerogenic myeloid phenotypes. Therapeutic agents targeting TIGIT or its PTM regulators (red) aim to restore immune activation within the TME

TIGIT is regulated by multiple PTMs, including phosphorylation, ubiquitination, and N-linked glycosylation, all of which shape its expression, stability, and immunoregulatory function within the TME (Fig. 4).

#### Phosphorylation

TIGIT is phosphorylated at tyrosine residues Y225 and Y231 within its intracellular ITIM and immunoreceptor tyrosine tail (ITT)-like motifs. Upon ligand engagement, these sites are phosphorylated by Src-family kinases (e.g., Lck, Fyn), facilitating the recruitment of Grb2, β-arrestin-2, and SHIP-1, which in turn suppress the AKT, MAPK, and NF-κB signaling pathways. This dampens cytokine production and cytotoxic function in T and NK cells [157]. Mechanistic studies have demonstrated that mutation of Y225 disrupts SHIP-1 recruitment and reverses the inhibitory effects of TIGIT on NK cells [158, 159]. Moreover, TIGIT phosphorylation has been shown to modulate the activity of CD226, a key co-stimulatory receptor. In dual PD-1/TIGIT blockade models, CD226 phosphorylation at Y322 is restored, highlighting a critical axis of regulatory crosstalk in T-cell activation [160, 161]. Overall, this phosphorylation-dependent suppression mechanism closely parallels that of PD-1, further reinforcing TIGIT’s role as a central inhibitory checkpoint in both adaptive and innate immune responses.

#### Ubiquitination

Recent findings demonstrate that STUB1, an E3 ubiquitin ligase, mediates K48-linked polyubiquitination of TIGIT, particularly under hypoxic conditions within tumors [162]. This promotes TIGIT degradation via the proteasome, thereby regulating its surface abundance and checkpoint function. This process also offers a rapid and reversible means of tuning immune suppression within the TME. Notably, phosphorylation of ITIM/ITT tyrosine residues may serve as a priming signal for E3 ligase recruitment, as observed in other inhibitory receptors. In parallel, its primary ligand, CD155, is also subject to TRAF6-mediated ubiquitination, which influences its membrane stability and ligand-receptor dynamics [163, 164]. Together, these findings underscore the importance of ubiquitination in regulating both TIGIT and its ligands, with implications for checkpoint stability, immune evasion, and therapeutic targeting in the TME.

#### Glycosylation

TIGIT undergoes N-linked glycosylation at conserved Asn-X-Ser/Thr motifs within its IgV-like extracellular domain. N32 and N101 have been identified as key sites essential for proper protein folding, membrane trafficking, and ligand binding [165]. Disruption of these glycosylation sites reduces TIGIT surface stability and impairs its interactions with CD155 and PVRL2, thereby compromising its immunoinhibitory function on T and NK cells [166]. Functionally, this glycan dependency parallels that of TIM-3, which forms Galectin-9-dependent lattices with PD-1 and exerts suppressive effects. Notably, TIGIT’s principal ligand, CD155, is also N-glycosylated specifically at N105, that enhances its surface expression and facilitates effective TIGIT engagement [167]. Collectively, these findings underscore the pivotal role of N-linked glycosylation in regulating the TIGIT–CD155 axis, with implications for immune suppression and therapeutic targeting within the TME.

#### Methylation

TIGIT expression is epigenetically regulated through DNA methylation, particularly within its promoter region. Recent studies have demonstrated that *TIGIT* promoter hypomethylation correlates strongly with elevated expression in various immune cell subsets within the TME. For instance, in HNSCC, *TIGIT* promoter hypomethylation was associated with increased TIGIT expression on TILs and a poor prognosis [168]. Similarly, single-cell transcriptomic analyses combined with methylation profiling in colorectal and gastric cancers revealed that hypomethylation of *TIGIT* enhances its transcriptional activation in exhausted T cells [169]. Functional experiments further confirmed that treatment with DNA methyltransferase inhibitors (e.g., 5-aza-2′-deoxycytidine) upregulates TIGIT expression in both CD4⁺ and CD8⁺ T cells [170], suggesting that DNA methylation acts as a gatekeeper of TIGIT expression in the immune system. These findings raise the possibility that epigenetic remodeling may synergize with checkpoint blockade by altering the threshold of TIGIT availability on T cells.

#### SUMOylation

In addition to ubiquitination, SUMOylation has emerged as a distinct post-translational mechanism regulating TIGIT. An earlier study found that SUMOylation of NK-activating ligands on tumor cells can alter their surface expression and impair NK cell recognition [171]. A recent study demonstrated that SUMOylation maintains the surface stability of CD155 and enhances its interaction with TIGIT on T and NK cells, thereby promoting immune suppression [172]. These findings suggest that SUMOylation of CD155 may act as an upstream regulator of TIGIT signaling by stabilizing ligand availability on tumor cells. This opens up new possibilities for combinatorial immunotherapy that targets both SUMO pathways and checkpoints, thereby overcoming tumor-induced immune tolerance.

### CTLA-4

**Fig. 5:**
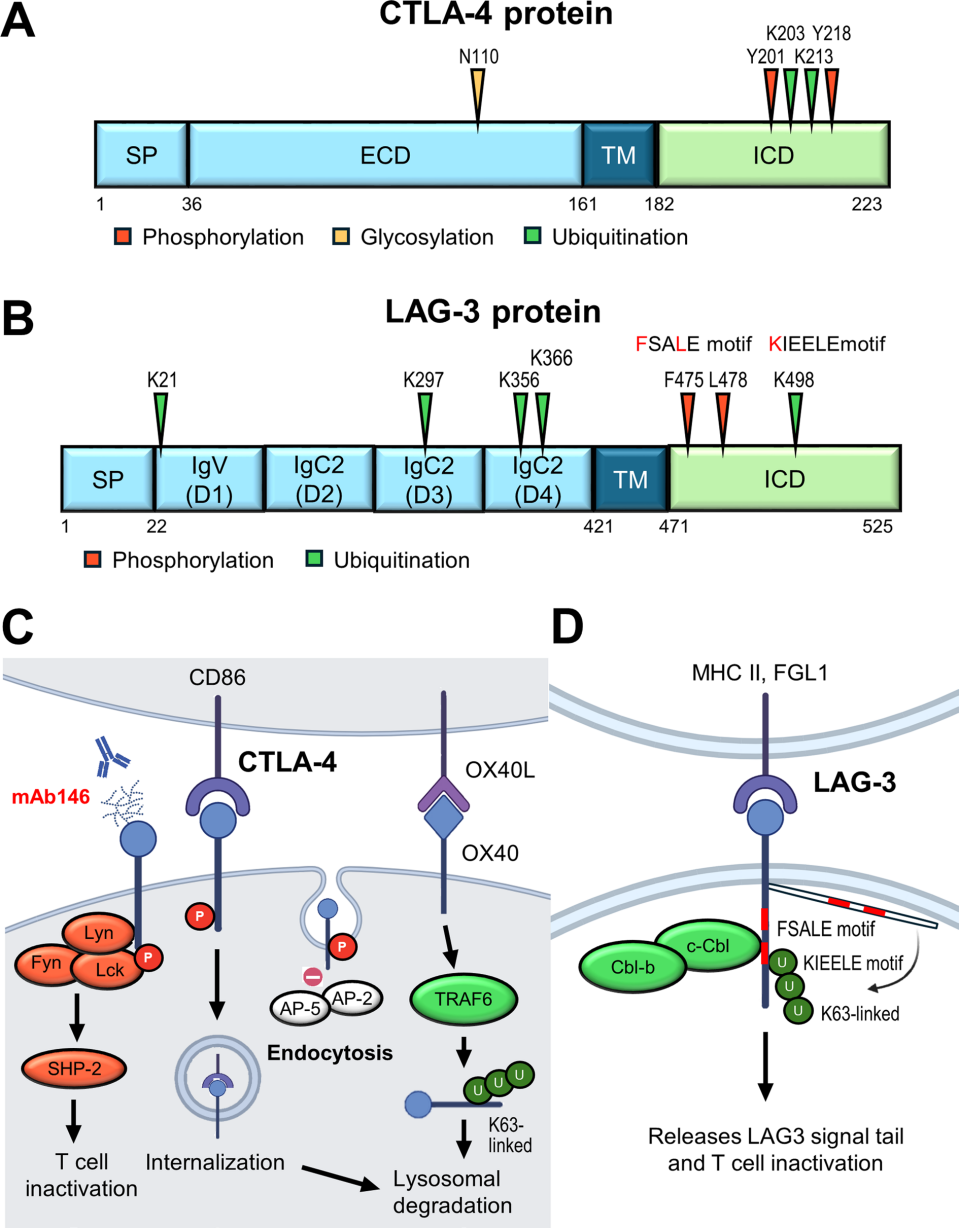
PTMs regulate the immunosuppressive functions of CTLA-4 and LAG-3. **A** and **B** Key asparagine, tyrosine, and lysine residues of CTLA-4 and LAG-3 are modified through phosphorylation, ubiquitination, and glycosylation. **C** Schematic of CTLA-4 phosphorylation-mediated suppression of T cell activity through SHP2 recruitment to dampen TCR signaling, ligand internalization, and inhibition of CTLA-4 endocytosis. TRAF6-mediated K63-linked lysosomal degradation regulates CTLA-4 protein turnover. Inhibitors shown in red letters bind to N110 on glycosylated CTLA-4 and interfere with the interaction between CTLA-4 and its ligand. **D** Ubiquitination by Cbl-b and c-Cbl releases the cytoplasmic tail from the membrane to execute LAG-3 suppressive function

Cytotoxic T-lymphocyte-associated protein 4 (CTLA-4), also known as CD152, is a pivotal checkpoint belonging to the immunoglobulin (Ig) superfamily. Under resting conditions, CTLA-4 is predominantly localized in intracellular vesicles. CTLA-4 is mainly expressed by CD4^+^ and CD8^+^ T cells, as well as Tregs, and to a lesser extent by B cells, innate lymphoid cells, DCs, and cancer cells [3]. CTLA-4 on leukemic B cells processes trans-endocytosis of CD80 on APCs. Insufficient expression of co-stimulatory molecules on APCs suppresses T cell activity and IL-2 production [173]. Moreover, deletion of CTLA-4 on antigen-loaded DCs augmented T cell response [174]. Upon TCR stimulation, intracellular CTLA-4 is rapidly mobilized toward the immunological synapse, the site of TCR engagement, where it exerts its regulatory effects [175]. CTLA-4 inhibits T cell activation by multiple mechanisms. For instance, as a homolog of CD28, CTLA-4 competes for the same ligands, CD80 and CD86, on APCs with a significantly higher affinity. Through this competitive binding, CTLA-4 interferes with CD28-mediated co-stimulatory signaling, resulting in the inhibition of both cytokine production and T-cell proliferation [176]. Moreover, CTLA-4 mediates the trans-endocytosis of CD80 and CD86 from the surface of APCs, thereby reducing the availability of these co-stimulatory ligands for CD28 interaction [177]. This mechanism is well-characterized in Treg, where CTLA-4 binds CD80/CD86 on APCs, sequestering them through trans-endocytosis and thus diminishing CD28-mediated activation of conventional T cells [178]. Therefore, CD28 on conventional T cells cannot interact with CD80/86, resulting in decreased T-cell activation [179]. In addition to competing ligand interaction, CTLA-4 is also able to recruit the phosphatase SHP-2 to the plasma membrane. SHP-2 dephosphorylates downstream targets of the TCR and CD28 signaling pathways, further reinforcing the suppression of T cell activation [180]. Together, these findings underscore the multifaceted role of CTLA-4 in immune regulation. The broad expression and function of CTLA-4 across multiple immune cell types highlight its central importance in maintaining immune homeostasis, as well as its potential as a therapeutic target in cancer immunotherapy (Fig. 5A, C).

#### Phosphorylation

The cytoplasmic domain of CTLA-4 contains two key tyrosine residues: Y201 and Y218. Phosphorylation of these residues plays a crucial role in modulating CTLA-4 localization and suppressive function. These tyrosine residues are initially discovered to be phosphorylated by Src family tyrosine kinases, such as Fyn, Lyn, and Lck upon T cell activation [181, 182]. Y201 is located in the YVKM motif, and its phosphorylation inhibits the interaction with the plasma membrane-associated adaptor complexes AP-2 and AP-50, thus preventing clathrin-mediated endocytosis and promoting transient surface retention of CTLA-4 [183, 184]. In addition to its role in trafficking, phosphorylated Y201 serves as a docking site for tyrosine phosphatase SHP-2 [185, 186]. SHP-2 subsequently dephosphorylates Src homology and collagen, thereby attenuating Ras signaling and suppressing TCR-driven T cell activation and cell proliferation [184]. Interestingly, the p85 subunit of PI3 kinase and JAK2 are also revealed to interact with phosphorylated Y201 of CTLA-4 [187].

Phosphorylation at Y218 has been linked to the interaction with the serine/threonine protein phosphatase PP2A, although the functional consequence of this association remains unclear [188]. Notably, a recent study suggests that Y218 phosphorylation of CTLA-4 was critical for ligand internalization in malignant B cells. Mutation of Y218 to phenylalanine impairs CD86 internalization and CTLA-4-CD86 ligation-mediated Tyk2/STAT3 activity, and reduction of B cell lymphoma cell proliferation [189]. Furthermore, the YVKM motif activity is required for LPS-responsive beige-like anchor protein (LRBA)-mediated vesicular trafficking of CTLA-4 to the cell surface. LRBA competes with AP-1 for binding to this motif, thereby protecting CTLA-4 from lysosomal degradation and promoting its surface expression [190]. Despite extensive studies, the implications of these PTMs remain partially inconsistent, warranting further investigation. Moreover, the role of serine/threonine phosphorylation in CTLA-4 regulation has yet to be elucidated.

#### Ubiquitination

CTLA-4 protein turnover is mainly regulated by lysosomal degradation. CTLA-4 expression is dynamic, and it rapidly undergoes ubiquitination after TCR activation. CTLA-4 contains five lysine residues in its cytoplasmic tail, among which ubiquitination of K203 and K213 is critical for lysosomal degradation. Yu et al. revealed that TNF receptor-associated factor 6 (TRAF6) mediates K63-linked polyubiquitination and subsequent lysosomal degradation of CTLA-4. T cell-specific TRAF6 knockout impairs the antitumor activity of CD8^+^ T cells [191]. In addition, the endosomal deubiquitinase USP8 has been identified as a scaffold protein that associates with the adaptor protein HD-PTP to facilitate efficient sorting of CTLA-4 to lysosomes [192]. However, the detailed molecular mechanisms underlying this process remain to be elucidated.

#### Glycosylation

CTLA-4 comprises two N-linked glycosylation sites at residues N78 and N110, both of which are implicated in CTLA-4 dimerization [193]. Although these residues are not directly involved in ligand binding, their glycosylation affects the overall conformation of the receptor, which in turn influences ligand interaction. Moreover, TCR signaling drives metabolite flux through the hexosamine and β1,6-GlcNAc branched N-glycosylation pathways, which in turn enhance CTLA-4 surface retention and subsequently contribute to sustained immune suppression [194]. Beyond N-glycosylation, advanced mass spectrometry techniques have also revealed O-glycosylation in CTLA-4-Ig fusion proteins. These O-glycans in the linker region interfere with the reformation of inter-chain disulfide bonds and reduce protein aggregation. This finding has significant implications for therapeutic protein design and provides a framework for assessing the structural similarity and stability of CTLA-4-Ig biosimilars [195, 196].

### LAG-3

Lymphocyte activation gene-3 (LAG-3), also known as CD223, is a transmembrane protein broadly expressed by T cells, NK cells, B cells, and plasmacytoid dendritic cells (pDCs) [5]. LAG-3 is a type I transmembrane glycoprotein comprised of four extracellular Ig-like domains (D1-D4), a connecting peptide, and an intracellular domain. Structurally, LAG-3 shares approximately 20% amino acid identity with CD4 and has a higher binding affinity to MHC class II molecules than CD4 [197]. Distinct from classical inhibitory receptors, LAG-3 lacks ITIM motifs in the cytoplasmic tail. Instead, it contains three conserved motifs that are critical for its immunomodulatory function. The KIEELE motif and FSALE are required for LAG-3-mediated inhibition [198, 199]. The EP (glutamic acid-proline rich tandem repeat) motif plays a crucial role in modulating LAG-3 trafficking to the cell surface following stimulation and promotes the dissociation of Lck from CD3/TCR, CD4, and CD8, thereby attenuating T-cell activation [200, 201].

LAG-3 inhibits T cell activation, cytokine production, and proliferation upon engagement with its canonical ligand MHC class II, thereby promoting tumor immune escape [181]. Moreover, LAG-3 contributes to an immunosuppressive environment via inducing IL-6 secretion and impairing IFN-α production in tumor-associated pDCs, and promotes monocytes to release CCL2, resulting in the recruitment of MDSCs [202]. LAG-3 signaling suppresses NK cell function by inhibiting IFN-γ production and downregulating the glycolysis pathway [203]. Recent findings identified other LAG-3-binding partners as the non-canonical ligands, including Galectin-3, liver and lymph node sinusoidal endothelial cell C-type lectin (LSECtin), α-synuclein, fibrinogen-like protein 1 (FGL1), and the TCR-CD3 complex (Fig. 5B, D).

#### Phosphorylation

A potential phosphorylation site has been identified at S484 within the FSALE motif. However, its functional relevance remains unclear, as a point mutation to alanine at this site does not alter the suppressive function of LAG-3. In contrast, residues F475 and L478 within the same FSALE motif play a critical role in inhibiting T cell activation by directly suppressing IL-2 secretion and its downstream cellular effects [204].

#### Ubiquitination

LAG-3 undergoes both linear and polyubiquitination. Linear ubiquitination is mediated by the linear ubiquitin chain assembly complex (LUBAC), while OTU deubiquitinase with linear linkage specificity (OTULIN) acts as a specific deubiquitinase. LAG-3 consists of five lysine residues (K21, K297, K356, K366, and K498). However, mutating the lysine to arginine results in only a slight reduction in LUBAC-mediated linear ubiquitination. Interestingly, this ubiquitination is markedly diminished when lysine mutations are combined with serine mutations (S484A or S497A). Despite these findings, the functional consequences of LUBAC/OTULIN-mediated linear ubiquitination on LAG-3 activity and its immunosuppressive role remain unclear [205]. Recently, Jiang and associates revealed that LAG-3 undergoes K11 and K63-linked non-degradative polyubiquitination upon engagement with ligands MHC class II and FGL1. In resting cells, the cytoplasmic FSALE motif is masked by membrane association through the basic-rich stretch, limiting its inhibitory signaling. Ligand engagement induces ubiquitination at K498 (located within the KIEELE motif) by E3 ligases c-Cbl and Cbl-b, which leads to dissociation of the cytoplasmic domain from the membrane and subsequently exposing its inhibitory FSALE motif [206].

#### Glycosylation

LAG-3 is a highly glycosylated protein that harbors multiple glycosylation sites in the extracellular domains. Accumulated reports suggest that glycosylation modulates interactions with certain ligands, Gal-3 and LSECtin binding [207, 208], whereas other ligands, such as FGL1, MHC-II, and α-syn, directly bind to LAG-3 at the D1 or D2 domain [197]. Thus far, the impact of glycosylation on LAG-3 function remains largely unexplored.

#### Ectodomain shedding

The cleavage of the LAG-3 ectodomain is mediated by ADAM10 and ADAM17 within the short connecting peptide between the membrane-proximal D4 domain and the transmembrane region, resulting in the release of soluble LAG-3 (sLAG-3) [209]. ADAM10 predominantly mediates constitutive cleavage, whereas ADAM17-dependent shedding is induced by TCR signaling in a protein kinase C theta-dependent manner. This PTM is reported to enhance T-cell proliferation and cytokine production [210]. Within the TME, sLAG-3 released from conventional CD4⁺ T cells has been shown to promote both CD4⁺ and CD8⁺ T-cell functionality. Clinically, low surface LAG-3 expression coupled with high ADAM10 expression on conventional CD4⁺ T cells correlates with improved survival and responsiveness to PD-1 blockade in patients with HNSCC and melanoma [211]. Circulating levels of sLAG-3 have been found to be elevated in various cancer types. Several studies have reported that high sLAG-3 expression is associated with advanced disease stages, including those in ccRCC and hepatocellular carcinoma (HCC) [212, 213]. However, in gastric cancer, increased sLAG-3 levels have been linked to stronger antitumor immune responses and reduced cancer progression [214], suggesting potential context-dependent roles. Importantly, elevated baseline sLAG-3 concentrations have been found in poor ICB response patients with solid tumors [215, 216]. Collectively, these findings indicate that sLAG-3 represents a dynamic immunoregulatory factor and a potential prognostic biomarker, though its predictive value requires further clinical validation.

### VISTA

V-domain immunoglobulin suppressor of T cell activation (VISTA), also known as B7‑H5, is an emerging checkpoint that plays a pivotal role in reshaping the TME, particularly through its regulation of MDSCs, macrophages, and DCs. Unlike PD-1 or CTLA-4, which primarily function in T cells, VISTA is highly expressed on myeloid lineage cells and mediates immunosuppression by promoting M2-like polarization, limiting the release of pro-inflammatory cytokines, and impairing antigen presentation [6, 217]. Studies have shown that VISTA expression is upregulated in tumor-infiltrating macrophages in response to TGF-β or IL-4 stimulation and correlates with a poor prognosis across several cancer types [218, 219].

Functionally, VISTA serves as both an inhibitory receptor and a ligand, depending on cellular context [220]. As a receptor on T cells, it transduces suppressive signals upon agonistic engagement, promoting peripheral tolerance by inducing apoptosis and clonal deletion of antigen-specific T cells. Conversely, VISTA deficiency disrupts T cell quiescence and enhances their spontaneous activation, underscoring its intrinsic role in maintaining T cell homeostasis [221]. As a ligand predominantly expressed on myeloid cells, VISTA interacts with counter-receptors such as P-selectin glycoprotein ligand-1 (PSGL-1) on T cells, particularly under the acidic condition characteristic of the TME. This interaction suppresses T cell proliferation and cytokine production, contributing to the development of immune-excluded or “cold” tumors [222, 223]. In addition, recent studies have identified leucine-rich repeats and immunoglobulin-like domains 1 (LRIG1) as a novel binding partner for VISTA that functions independently of pH, further contributing to CD8⁺ T cell dysfunction and tumor immune evasion [224]. Moreover, single-cell transcriptomic and functional analyses have identified VISTA as a hallmark of immunosuppressive TAMs and tolerogenic DC subsets [225]. The VISTA⁺ myeloid populations exhibit diminished antigen-presenting capacity and suppress T cell priming through STAT3 activation and polyamine-driven metabolic reprogramming, consequently limiting effective antitumor immune responses [226].

**Fig. 6:**
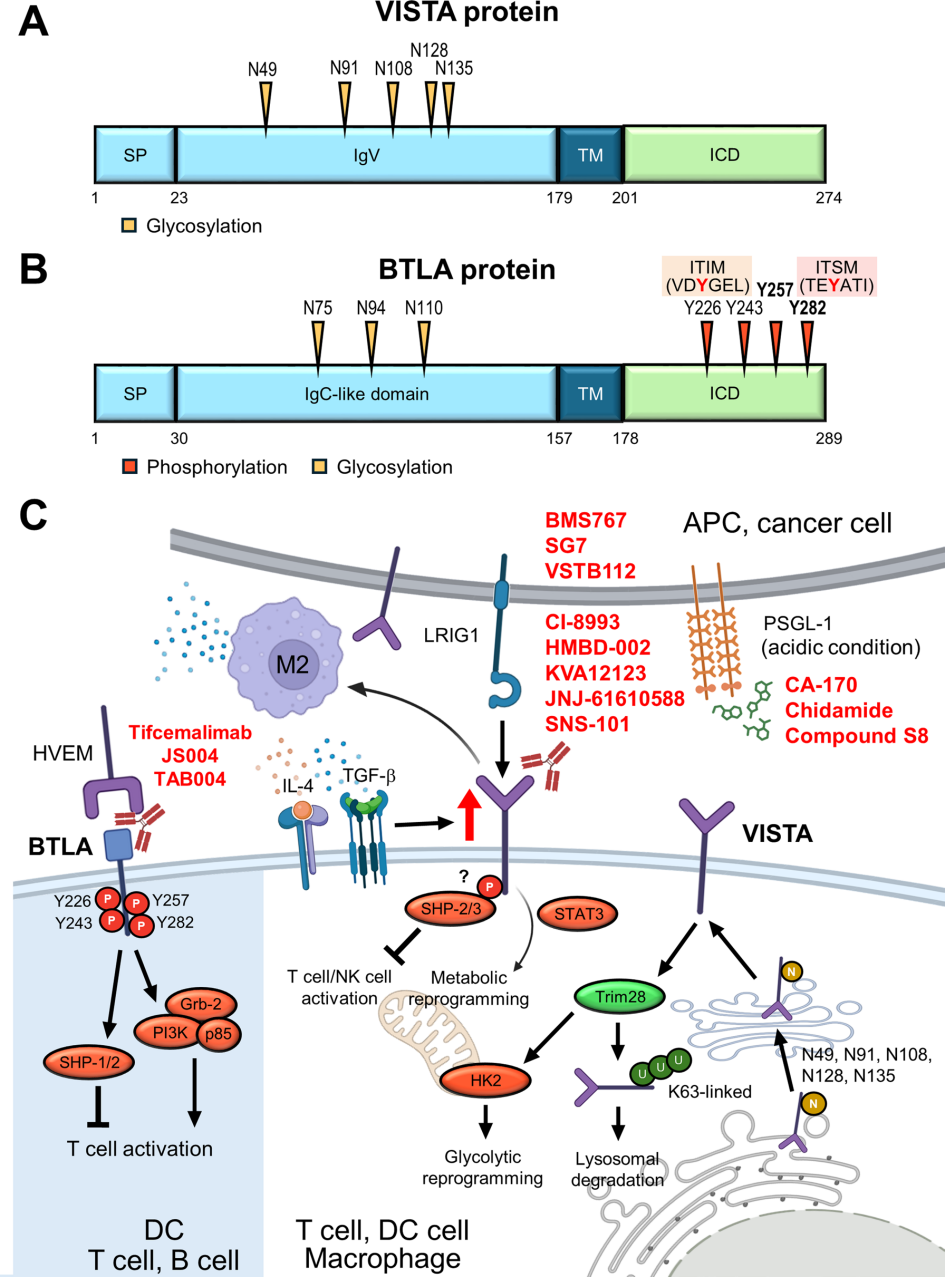
VISTA and BTLA integrate distinct PTMs and ligand interactions to suppress immune activation across myeloid and lymphoid compartments. **A** Key asparagine residues of VISTA are modified through glycosylation. **B** Key glycosylation and phosphorylation residues of BTLA. **C** Mechanistic illustration of VISTA and BTLA checkpoint signaling. In the TME, VISTA is highly expressed on myeloid cells and transmits suppressive signals through ligand interactions (e.g., PSGL-1 under acidic pH, LRIG1), activation of SHP2 and STAT3, and TRIM28-mediated K63-linked ubiquitination that enhances glycolytic reprogramming via HK2. Glycosylation is critical for VISTA surface localization and function (right). BTLA, in contrast, acts predominantly in T cells, where HVEM engagement induces phosphorylation at Y226/Y243 via Src-family kinases (Lck, Fyn), leading to SHP1/2 recruitment and suppression of TCR signaling (left). Multiple VISTA-targeting monoclonal antibodies and small molecules, shown in red letters, are under development to block its receptor–ligand interaction and restore antitumor immunity (upper)

PTMs are emerging as crucial regulators of VISTA that can influence its stability, trafficking, and immune regulatory function in the TME [227]. While PTMs-mediated controls have been well-characterized for other checkpoints, such as PD-1 and TIM-3, our understanding of VISTA’s PTM landscape remains limited but is rapidly evolving. Among these, glycosylation and ubiquitination represent the best-characterized PTMs, with functional implications in both adaptive and innate immune compartments (Fig. 6A, C).

#### Phosphorylation

VISTA differs from PD-1 in that its cytoplasmic tail lacks ITIM/ITSM motifs, and site-specific phosphorylation has not yet been shown. Work to date emphasizes pH-dependent ligand biology and immune modulation rather than defined cytoplasmic signaling. While YxxQ and PxxP motifs have been noted, they have not been validated as phosphorylation-dependent modules in VISTA [228, 229]. Likewise, despite β-TrCP’s recognition of phosphodegrons such as DSGxxS in other substrates, no phosphodegron or priming kinase has been identified for VISTA [230, 231]. Mapping VISTA phosphorylation, if present, is therefore a meaningful next step that could uncover actionable couplers between extracellular cues and receptor turnover/signaling.

#### Ubiquitination

Recent evidence has revealed that VISTA is subject to K63-linked ubiquitination, a PTM that modulates its immunoregulatory function in innate immune cells. In a study of sepsis-associated encephalopathy, TRIM28, an E3 ubiquitin ligase, was shown to catalyze K63-linked polyubiquitination of VISTA in microglia, leading to its stabilization and enhanced signaling activity. Mechanistically, ubiquitinated VISTA promotes the expression of hexokinase 2 (HK2) and activates glycolytic reprogramming, which contributes to M2-like polarization of microglia and suppression of neuroinflammation. Importantly, genetic or pharmacologic disruption of TRIM28-mediated ubiquitination reduces VISTA levels and restores inflammatory responses [232]. Although this study was conducted in the context of central nervous system inflammation, it provides compelling support that E3 ligase-dependent ubiquitin signaling regulates VISTA function beyond cancer, and raises the possibility that similar ubiquitin-mediated pathways may modulate VISTA activity in TAMs or DCs. These findings highlight ubiquitination as a key PTM controlling stability and immunosuppressive capacity of VISTA, and suggest TRIM28 as a potential upstream regulator of VISTA in diverse pathological settings.

#### Glycosylation

A recent study demonstrated that VISTA is heavily N‑glycosylated at multiple conserved asparagine residues (N49, N91, N108, N128, N135) within its IgV ectodomain. Targeted mutagenesis (N to A) or enzymatic deglycosylation with PNGase F significantly reduced its molecular weight, impaired stable cell surface expression, and altered subcellular localization in renal carcinoma cells (RCC) [233]. Glycosylation-deficient VISTA also exhibited decreased ligand-binding capacity, indicating that these glycans are structural determinants of both protein trafficking and functional interactions, a mechanism reminiscent of glycosylation roles in other B7-family checkpoints.

#### Acetylation

Emerging evidence indicates that acetylation-related epigenetic mechanisms regulate VISTA expression in innate immune cells. In LPS-stimulated microglia, loss of H3K27ac at the *Vsir* promoter and enhancer regions correlates with reduced transcription alongside decreased chromatin accessibility for PU.1 and other myeloid transcription factors. Although direct lysine acetylation of VISTA protein was not shown, the results support a model where chromatin acetylation dynamics repress VISTA under inflammatory conditions. While this study did not directly demonstrate lysine acetylation on the VISTA protein itself, it provides compelling support that VISTA expression is modulated at the chromatin level via acetylation dynamics, particularly under inflammatory conditions [234].

A recent publication expands on this concept by illustrating that histone deacetylase (HDAC) activity is frequently upregulated in inflammatory or TMEs, which can further suppress checkpoint genes via chromatin remodeling. Together, these data suggest that HDAC inhibitors or histone acetyltransferase modulators may restore VISTA expression in specific cellular settings, enabling either the upregulation or downregulation of immunosuppressive signaling, depending on the disease context [235].

### BTLA

B and T lymphocyte attenuator (BTLA), a member of the CD28 immunoglobulin superfamily, functions as an inhibitory checkpoint primarily expressed on activated T cells, B cells, DCs, and some innate lymphoid subsets. Its ligand, herpesvirus entry mediator (HVEM), is broadly expressed on hematopoietic and non-hematopoietic cells, including tumor cells [8]. Within the TME, the BTLA-HVEM axis delivers inhibitory signals that suppress effector T cell activity, promote exhaustion, and reduce cytokine production, particularly in CD8⁺ tumor-infiltrating lymphocytes [236]. Phosphorylation is not only the fundamental PTM of BTLA currently supported by mechanistic data, but also a critical modulator of its suppressive function in the TME and poor clinical outcomes [237, 238]. BTLA is frequently co-expressed with other inhibitory receptors [18]. Future studies exploring additional PTMs of BTLA and developing pharmacologic agents to target its phosphorylation axis could offer new avenues for immunomodulatory therapies (Fig. 6B, C).

#### Phosphorylation

BTLA transduces inhibitory signals upon engagement with its ligand HVEM through phosphorylation of two conserved tyrosine motifs within its cytoplasmic domain: an ITIM and an ITSM, corresponding to Y257 and Y282, respectively. Interestingly, additional phosphorylation sites (Y226 and Y243) via Grb-2–mediated recruitment of PI3K have been reported. These phosphorylation sites promote T cell activation rather than inhibition. Upon HVEM binding, Src family kinases phosphorylate these tyrosine residues, enabling the recruitment of the phosphatases SHP-1 and SHP-2. These phosphatases then attenuate TCR signaling by dephosphorylating key downstream effectors, including CD3ζ and ZAP70, thereby suppressing T-cell activation and IL-2 production. Mutational analyses further demonstrate that disruption of these tyrosine residues abrogates BTLA’s inhibitory function, underscoring their essential role in immune suppression [239, 240].

#### Glycosylation

BTLA is a glycoprotein whose Ig-like extracellular domain carries three conserved N-linked glycosylation sites, including N75, N94, and N110, which are independently leveraged by glyco-selective antibody designs [241]. BTLA engages the N-terminal cysteine-rich domain of HVEM via a binding surface distinct from other CD28-like receptors, indicating that core recognition is preserved despite peripheral glycan decoration [242]. Functionally, N-glycan branching promotes BTLA endocytosis, thereby decreasing the surface abundance of BTLA; conversely, reduced branching elevates surface BTLA and strengthens its inhibitory restraint on hyperactive T cells. Although single-site functional dissection remains limited in the literature, current data establish BTLA N-glycosylation, particularly branching state, as a key determinant of BTLA surface turnover [243]. From a translational perspective, agents targeting the BTLA–HVEM interface should account for glycosylation-dependent trafficking, since glycan-tuned endocytosis and surface levels can modulate effective target engagement at the plasma membrane [244].

### SIRPα

Signal regulatory protein alpha (SIRPα) is an inhibitory receptor primarily expressed on myeloid cells, where it plays a pivotal role in sculpting the immunosuppressive landscape of the TME via both CD47-dependent and -independent mechanisms. The canonical CD47-SIRPα "don't eat me" axis inhibits macrophage-mediated phagocytosis by localizing SIRPα to the phagocytic synapse and suppressing integrin activation [7, 245]. Disruption of this axis restores phagocytosis, promotes the presentation of tumor antigens, and enhances T cell priming. Recent studies further show that SIRPα blockade reverses immunosuppression in TAMs and reprograms MDSCs toward pro-inflammatory phenotypes. Blocking CD47-SIRPα signaling restores reactive oxygen species (ROS) production and antigen presentation in MDSCs, thereby improving anti-tumor immunity [246]. In addition, single-cell transcriptomic profiling has identified a novel subset of SPP1⁺ macrophages expressing SIRPα that were enriched in the TME and associated with therapeutic resistance, highlighting SIRPα’s role in TAM-driven immune evasion [247].

Beyond CD47, SIRPα exerts intrinsic immunoregulatory effects. In colorectal cancer, SIRPα-expressing TAMs suppress antitumor responses independently of CD47, whereas SIRPα-deficient macrophages enhance CD8⁺ T cell activation, particularly when combined with radiotherapy [248, 249]. SIRPα also regulates innate lymphoid cells, particularly group 2 ILCs (ILC2s). Its engagement suppresses IL-5 and IL-13 production by modulating JAK/STAT and ERK/MAPK signaling and cellular metabolism. Genetic deletion or antibody blockade of SIRPα enhances cytokine secretion and effector functions in ILC2s [250]. Clinically, elevated SIRPα expression in HCC correlates with a poor prognosis and increased infiltration of immunosuppressive myeloid cells; therefore, it may serve as a potential prognostic biomarker [251]. Therapeutically, SIRPα blockade promotes pro-inflammatory polarization of TAMs and DCs, boosts CD8⁺ T cell-mediated immunity, and suppresses tumor progression in preclinical models [252]. Together, these findings establish SIRPα as a multifaceted regulator of immune suppression, integrating myeloid, lymphoid, and stromal interactions to reshape the TME and influence immunotherapeutic outcomes.

**Fig. 7:**
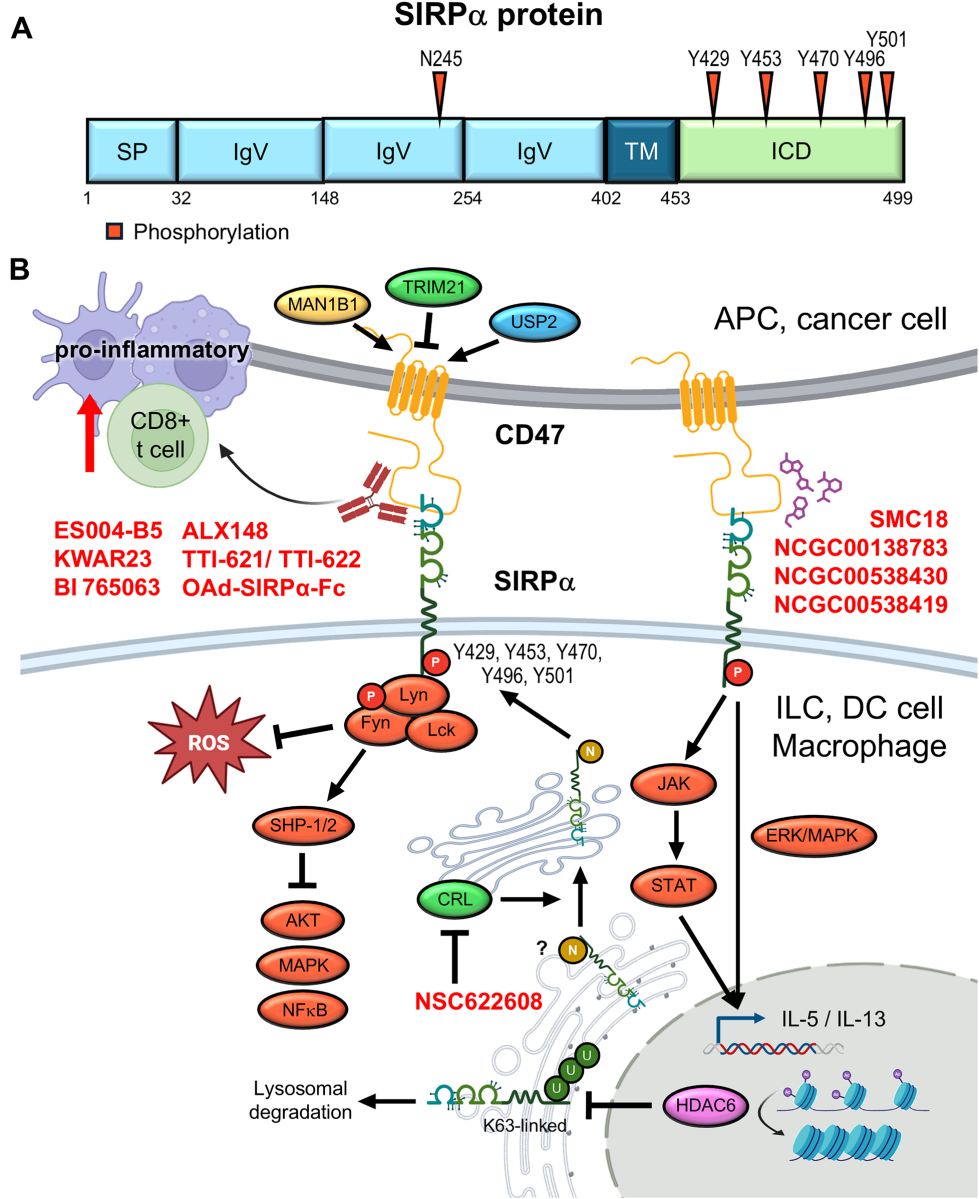
SIRPα checkpoint activity is regulated by emerging PTMs that shape myeloid immunosuppression in the TME. **A** Key lysine residues of SIRPα are modified through phosphorylation. **B** Mechanistic schematic of SIRPα signaling. CD47 engagement induces the tyrosine phosphorylation of SIRPα and the activation of SHP1/2, thereby inhibiting phagocytosis, ROS production, and inflammatory responses in TAMs, DCs, and MDSCs. Beyond CD47, SIRPα also suppresses ILC2 cytokine production through JAK/STAT and MAPK pathways. HDAC6 inhibition enhances SIRPα acetylation and promotes lysosomal degradation, thereby restoring phagocytic function. Glycosylation mediates SIRPα cis-clustering and membrane organization, while neddylation blockade destabilizes CRL targets and downregulates SIRPα expression. Multiple blocking antibodies and inhibitors (red) targeting these regulatory nodes may potentiate CD47-SIRPα blockade therapies

PTMs critically shape the immune-regulatory landscape orchestrated by SIRPα within the TME. As a central myeloid checkpoint receptor, SIRPα integrates extracellular cues and intracellular signaling cascades to modulate phagocytosis, cytokine responses, and antigen presentation. These regulatory processes are not solely controlled by ligand availability but are also dynamically influenced by PTMs that alter stability, membrane localization, and signaling potential of SIRPα. In the TME, such PTMs may determine whether SIRPα sustains an immunosuppressive state favoring tumor progression or shifts toward a pro-inflammatory phenotype that supports antitumor immunity. Emerging evidence suggests that therapeutic manipulation of these PTMs can destabilize SIRPα or interrupt its downstream inhibitory circuits, thereby reactivating innate immune effectors such as TAMs and DCs. Thus, understanding how SIRPα is regulated at the post-translational level provides valuable insight into its context-dependent function and offers new strategies to fine-tune immune responses against cancer [253] (Fig. 7).

#### Phosphorylation

SIRPα harbors multiple ITIM tyrosine residues (e.g., Y429, Y453, Y470, Y496) that are phosphorylated by Src family kinases upon engagement of CD47 or integrins. This phosphorylation event recruits SHP-1 and SHP-2, inhibiting downstream PI3K-Akt2, MAPK, and NF-κB pathways to suppress phagocytosis and inflammatory activation [254, 255].

In macrophages, phosphorylated SIRPα dampens proinflammatory responses and facilitates tumor immune evasion, whereas its inhibition enhances ROS production, antigen presentation, and antitumor immunity [256]. CD47 ligation alone elicits modest phosphorylation, but inflammatory stimuli such as LPS and IFN-γ substantially amplify Src-mediated phosphorylation and immunosuppressive signaling [257]. In podocytes, SIRPα phosphorylation modulates cytoskeletal organization via FAK at Y597 and influences immune signaling through Syk [258]. Elevated phosphorylation at Y501 has also been observed in rat glomeruli, indicating conserved regulatory functions beyond immune cells [259]. Altogether, phosphorylation functions as a molecular switch for SIRPα, enabling SHP-mediated immunosuppression, while its dephosphorylation or cleavage relieves this inhibition and promotes inflammatory responses. Within the TME, this phosphorylation-dependent axis is pivotal for myeloid suppression and represents a potential target to boost antitumor immunity [260].

#### Ubiquitination

SIRPα is a key checkpoint that transduces inhibitory signals through its interaction with CD47. Despite its central role in immune regulation, there is currently no direct evidence that SIRPα is subjected to ubiquitination. In contrast, multiple studies have demonstrated that CD47 is tightly regulated via ubiquitination and deubiquitination, potentially impacting SIRPα signaling. For example, USP2 has been shown to deubiquitinate and stabilize CD47, whereas inhibition of USP2 enhances its degradation and promotes macrophage phagocytosis [261]. TRIM21, an E3 ligase, mediates K48-linked ubiquitination and proteasomal degradation of CD47, which is attenuated by Src-induced phosphorylation following EGFR activation [262]. Deglycosylation of CD47 by MAN1B1 also promotes its internalization and antitumor immune activation, although the involvement of ubiquitination was not directly investigated [263]. Additionally, CD47 ubiquitination has been reported to facilitate both lysosomal and proteasomal degradation, thereby amplifying innate immune responses [264]. While the ubiquitination of SIRPα remains uncharacterized, the loss or degradation of CD47 may alter the fate of its receptor. Therefore, future investigations could focus on identifying E3 ubiquitin ligases that interact with SIRPα and evaluating whether mutating its intracellular lysine residues affects receptor stability under conditions of ligand depletion or proteasome inhibition.

#### Glycosylation

Although glycosylation of SIRPα is not essential for its trans-interaction with CD47, N-linked glycans appear to play a structural role in modulating receptor conformation and membrane organization. In particular, glycan modifications have been shown to facilitate cis-dimerization of SIRPα on the cell surface, which may influence its spatial distribution and functional regulation [265]. Disruption of these cis interactions between SIRPα and CD47 has been shown to enhance macrophage-mediated phagocytosis, suggesting that cis-clustering of SIRPα contributes to the restraint of checkpoint signals at the cell surface [266]. These findings suggest that glycosylation may indirectly regulate immune responses by influencing the availability and spatial dynamics of SIRPα through cis-association.

#### Acetylation

Current evidence supports an HDAC6-dependent control of the CD47–SIRPα checkpoint at the level of receptor turnover. Pharmacologic HDAC6 inhibition increases macrophage phagocytosis and reduces SIRPα abundance, thereby potentiating anti-CD47 therapy [267, 268]. Mechanistically, these effects are consistent with HDAC6-regulated trafficking toward lysosomal degradation (e.g., altered endolysosomal flux and surface residency), but site-resolved acetylation on human SIRPα has not yet been mapped. In vivo, HDAC6 blockade also remodels the myeloid compartment (promoting M1-like polarization) and enhances CD8⁺ T-cell infiltration, creating synergy with anti-CD47 in tumor growth control [269]. Taken together, HDAC6 acts as an actionable upstream regulator of SIRPα checkpoint output, while the exact acetyl-lysine residues and directness of the modification remain to be defined.

#### Neddylation

Recent studies have shown that inhibition of neddylation using the NEDD8-activating enzyme (NAE) inhibitor MLN4924 (Pevonedistat) can enhance macrophage-mediated phagocytosis. Mechanistically, this effect is driven by inactivation of Cullin-RING E3 ligases (CRLs), leading to altered turnover of regulatory proteins involved in SIRPα signaling. Although SIRPα itself is not directly neddylated, NAE inhibition reduces its surface expression, possibly through destabilization of upstream factors or by promoting lysosomal degradation, thereby relieving the inhibitory checkpoint imposed by the CD47-SIRPα axis [270]. This work further supports the role of neddylation in modulating innate immune checkpoints by demonstrating that CRL inactivation reprograms macrophage phenotype toward a pro-phagocytic state. Neddylation blockade enhances antigen presentation, increases pro-inflammatory cytokine production, and promotes the internalization and degradation of SIRPα. The findings suggest that targeting the neddylation-CRL system indirectly suppresses SIRPα-mediated inhibition and enhances the efficacy of anti-CD47 immunotherapy [271].

## Targeting PTMs of checkpoints

While monoclonal antibodies targeting PD-L1, PD-1, or CTLA-4 have transformed cancer immunotherapy, resistance to immunotherapy, both primary and acquired, remains a major clinical challenge [272]. For patients unresponsive to ICB, PTMs present two promising solutions. First, checkpoint degradation via small-molecule intervention bypasses ligand-receptor dependence and mitigates receptor redundancy. Second, expanding therapeutic focus beyond T cell-tumor interactions to encompass the broader immune ecosystem holds transformative potential [22]. Targeting PTMs in these compartments may reprogram the suppressive TME, enhance antigen presentation, and improve response rates in otherwise non-responsive patients. Table 2 integrates these PTM-targeted approaches as a new paradigm, transitioning from receptor blockade to functional rewiring, which enables more durable and robust immune activation [273]. The drugs described in Figs. 1, 2, 3, 4, 5, 6 and 7 are included with their clinical status emphasized (Table 2).

### PD-L1

Recent research highlights that pharmacologically destabilizing PD-L1 through PTMs can overcome resistance and enhance the therapeutic potency of ICB. Targeting upstream kinases can activate E3 ligases that mark PD-L1 for proteasomal degradation. For example, ES-072, an EGFR inhibitor, induces GSK3α activation, which recruits the E3 ligase ARIH1 to ubiquitinate PD-L1, thereby enhancing its degradation. Combining ES-072 with anti-CTLA-4 significantly suppresses tumor growth in mouse breast allografts [27]. Similarly, the CK2 inhibitor CX4945 promotes CUL3-mediated PD-L1 degradation and synergizes with Tim-3 blockade in syngeneic mouse models of breast cancer [31]. Moreover, GSK2578215 inhibits LRRK2 activity, downregulating PD-L1 stability and sensitizing PDAC to PD-L1 blockade [32].

Metabolic reprogramming is another effective strategy to modulate PD-L1 stability in cancer. Metformin, a well-known anti-diabetic drug, has been shown to directly induce antitumor effects by inhibiting the PI3K-Akt-mTOR and Ras-MAPK signaling pathways. Metformin also induces PD-L1 degradation via AMPK activation and robustly suppresses breast tumor growth when combined with CTLA-4 blockade [29]. Likewise, AMPK agonist A-769662 reduces PD-L1 protein levels and improves anti-CTLA-4 efficacy in colon tumor models [28]. AMPK agonist induces the degradation of both PD-1 and PD-L1, underscoring its potent immunomodulatory effects. Canagliflozin, an SGLT2 inhibitor, disrupts the interaction between SGLT2 and PD-L1, exposing PD-L1 to SPOP-mediated ubiquitination and proteasomal degradation [37].

Targeting glycosylation to destabilize PD-L1 is also an emerging area. Gefitinib, an EGFR inhibitor, reduces PD-L1 glycosylation and its interaction with PD-1, thereby amplifying the anti-PD-1 immune response [26]. Etoposide, a chemotherapeutic agent, suppresses STT3 isoforms and reduces glycosylated PD-L1 levels, thereby enhancing the efficacy of Tim-3 blockade [58]. In addition, a glyco-specific antibody, STM108, induces the internalization and degradation of PD-L1; its conjugation with the potent antimitotic drug monomethyl auristatin E results in significant tumor shrinkage [57].

Inhibiting deubiquitinases to destabilize PD-L1 is another promising strategy. For instance, Curcumin, a CSN5 inhibitor, decreases PD-L1 expression and enhances the cytotoxic T cell activity. Combining curcumin with anti-CTLA-4 leads to marked tumor suppression [49]. ML364, a selective USP2 inhibitor, destabilizes HIV-1 Vpr binding protein kinase and indirectly increases PD-L1 expression, thereby improving the tumor response to anti-PD-1 treatment [53]. Furthermore, the reduction of palmitoylation by inhibiting specific enzymes shows therapeutic potential. 2-Bromopalmitate inhibits the palmitoylation process, which is crucial for PD-L1 membrane stability, and enhances T-cell immunity against colon cancer [68]. Delivering siRNA of ZDHHC9 palmitoyltransferase using polymeric nanoparticles, combined with anti-PD-L1 therapy, significantly delays tumor growth [274]. Other novel strategies, such as a competitive peptide PTPR, disrupt the TMUB1-PD-L1 interaction, promoting PD-L1 degradation and boosting anti-CTLA-4 efficacy [38].

Furthermore, an IL-6 monoclonal antibody blocks the IL-6/JAK1-mediated stabilization of PD-L1, thereby boosting the effectiveness of anti-TIM-3 therapy [30]. NU-7441, a selective DNA-PK inhibitor, decreases PD-L1 stability and shows potent tumor growth inhibition and a significant increase in T cell infiltration [33]. Together, these findings underscore the potential of targeting diverse PTMs and associated pathways to destabilize PD-L1 to enhance the overall therapeutic benefit of ICB.

### PD-1

Several small molecules and biological agents have been identified to modulate PD-1 protein stability through PTMs, thereby enhancing antitumor immune responses. Approaches to either augment E3 ligase activity or inhibit deubiquitinases to degrade PD-1 are an emerging area of research. Oridonin, a natural diterpenoid, activates the E3 ligase FBW7, thereby enhancing its interaction with PD-1 and promoting PD-1 ubiquitination and degradation. Combination treatment with Oridonin and PD-1 blockade results in significantly greater antitumor efficacy compared to monotherapy [88]. Moreover, IL-2 treatment restores the transcription of the E3 ligase FBXO38 in tumor-infiltrating T cells, resulting in reduced PD-1 expression and enhanced T cell function [101]. IFN-α induces and activates p53, which upregulates MDM2 in both cancer cells and T cells. MDM2 subsequently reduces glycosylated PD-1 levels. Combination therapy with IFN-α and PD-1 blockade produces a remarkable synergistic effect in colon cancer models. Importantly, this effect is dependent on functional MDM2, as it is abrogated in Mdm2⁻/⁻ T cells [104].

To target PD-1 deubiquitination, specific inhibitors of deubiquitinases have been explored. ERK-mediated phosphorylation of PD-1 enhances its interaction with the deubiquitinase USP5, thereby stabilizing PD-1 protein. Combined treatment with the USP5 selective inhibitor EOAI3402143 and the MEK inhibitor trametinib effectively suppresses colon tumor growth in mice, showing superior tumor control compared to anti-PD-1 therapy. In addition, the combination of EOAI3402143 with anti-CTLA-4 further improves OS [100]. An additional report suggests that targeting PD-1 stability with the USP24-specific inhibitor USP24-i-101 promotes PD-1 degradation, boosts cytotoxic T cell activity, suppresses lung tumor growth, and shows superior therapeutic effects when combined with anti-CTLA-4 immunotherapy [106].

Beyond ubiquitination, several studies focused on interfering with other PTMs of PD-1, including glycosylation, palmitoylation, and UFMylation. For instance, the monoclonal antibody STM418 specifically recognizes glycosylated PD-1, exhibiting a higher binding affinity than nivolumab or pembrolizumab, which effectively blocks the PD-1/PD-L1 interaction and enhances antitumor immunity [108]. Moreover, 2-fluoro-L-fucose (2F-Fuc), a metabolic fucosylation inhibitor, enhances the functionality of T cells. 2F-Fuc-treated OT-I T cells exhibit improved intratumoral expansion and elevated inflammatory cytokine production. Combination with anti-PD-1 therapy yields a more durable therapeutic response in the later stages of treatment [110]. Palmitoylation of PD-1 by DHHC9 maintains the stability of PD-1. Disruption of this PTM using competitive peptides such as PD1-PALM reduces PD-1 expression in vitro in a dose-dependent manner. However, its in vivo efficacy remains to be validated [113]. AMPK activation by MK-8722 disrupts PD-1 UFMylation, triggering its ubiquitination and degradation. Combined treatment with MK-8722 and anti-CTLA-4 shows a synergistic tumor-suppressive effect [114].

### TIM-3

Recent research has highlighted TIM-3 as a promising and multifaceted immunotherapy target across diverse tumor types, especially when co-targeted with PD-1 [275, 276]. Beyond ICB, there is growing interest in exploiting TIM-3’s post-translational regulation and receptor biology to enhance antitumor immunity. Notably, palmitoylation of TIM-3 at C296 by DHHC9 stabilizes its surface expression and supports immune suppression, whereas blocking this PTM promotes proteasomal degradation and restores effector T and NK cell functions [143]. Emerging strategies now include both monoclonal antibodies and small molecules that disrupt ligand binding or receptor stability. Among antibody-based therapies, agents such as Sym023, MBG453, BGB-A425, LY3321367, TSR-022, and INCAGN02390 have progressed through early-phase clinical trials, demonstrating safety and preliminary efficacy, particularly in combination with PD-1 blockade [277–280]. Bispecific antibodies, such as RO7121661 (PD-1/TIM-3) and LY3415244 (TIM-3/PD-L1), aim to synergize dual checkpoint inhibition but face challenges, including immunogenicity [281]. Meanwhile, ML-T7, a small molecule inhibitor, targets TIM-3–ligand interactions and has shown strong preclinical efficacy, restoring CD8⁺ T cell function and synergizing with anti-PD-1 therapy [282]. TIM-3 targeting also holds potential beyond T cells, including in NK cells and DCs, broadening its impact within the TME [283]. Collectively, these findings underscore TIM-3 as both a checkpoint and a regulatory hub, with ongoing therapeutic innovations aiming to manipulate its signaling, ligand binding, and stability to overcome tumor immune evasion.

### TIGIT

In addition to its intrinsic inhibitory signaling, TIGIT modulates immune suppression through FcγR engagement when targeted by Fc-competent antibodies such as tiragolumab, ociperlimab (BGB-A1217), or TIGIT × 4-1BB bispecific antibody [284–286], leading to enhanced MHC class II expression, improved antigen presentation by TAMs and DCs, and reprogramming of exhausted CD8⁺ T cells into memory-like subsets [287]. While monoclonal antibodies remain the mainstream approach, increasing efforts have been directed toward small-molecule and peptide-based inhibitors, particularly those modulating PTMs or directly blocking TIGIT-PVR interactions [288, 289].

Notably, Liothyronine, a thyroid hormone analog, was repositioned to disrupt TIGIT-PVR binding and restore CD8⁺ T cell effector function in preclinical models. Hemin, a heme derivative, and Azelnidipine [290–292], a calcium channel blocker, also exhibited inhibitory activity against the TIGIT-CD155 axis, showing immune-restorative effects even in PD-1-resistant tumors. Furthermore, 9-ING-41 (Elraglusib), a GSK-3β inhibitor, indirectly blocks TIGIT signaling while enhancing cytokine production and tumor regression [293]. Dual inhibitors such as Gln(TrT) simultaneously target the TIGIT/PVR and PD-1/PD-L1 axes, promoting CD8⁺ T cell activation [294, 295]. Advanced drug screening using DNA-encoded library and machine learning approaches has yielded novel small molecules with micromolar inhibitory potency against TIGIT–CD155 binding [296]. D-peptides generated via phage display disrupt TIGIT/PVR engagement and restore T cell functionality [297]. Importantly, pharmacologic targeting of PTMs has emerged as a novel strategy to regulate TIGIT indirectly. TAK-981, a SUMOylation pathway inhibitor, was shown to decrease the expression of CD155 (PVR), the primary ligand for TIGIT, thereby amplifying the efficacy of TIGIT blockade by promoting CD8⁺ T cell infiltration and activity within the TME [172].

A growing number of clinical-stage anti-TIGIT agents are under development. Among the most advanced are domvanalimab (AB-154) [298] and vibostolimab (MK-7684) [299], all of which have demonstrated TIGIT blockade with Fc-effector engagement and favorable profiles in early-phase trials. Other antibodies including EOS-448, COM-902, and BMS-986207, and ASP-8374 [300–303], were each engineered with distinct Fc formats and binding epitopes to maximize immune activation while minimizing toxicity [304]. Importantly, etigilimab (OMP-313M32) evaluated in combination with nivolumab in a Phase 1a/b trial, shows promise in reversing T cell exhaustion via TIGIT blockade [305]. Additionally, M6223 blocks TIGIT ligand binding and mediates FcγR-driven depletion of TIGIT⁺ regulatory and exhausted T cells [306]. Collectively, these therapies represent a maturing class of checkpoint inhibitors, often paired with PD-1 blockade to enhance clinical efficacy, with ongoing studies dissecting their synergistic mechanisms, pharmacokinetics, and long-term benefit [155].

### CTLA-4

Several studies have highlighted promising strategies to modulate CTLA-4 stability and targeting its PTMs. OX86, an anti-OX40 agonist antibody, activates the OX40–OX40L signaling pathway to induce expression of TRAF6-mediated K63-linked polyubiquitination, which accelerates CTLA-4 degradation. Treatment with OX86 significantly enhances the activity of TILs and synergizes with anti-PD-L1 therapy, resulting in improved antitumor effects [192]. In addition, Li and associates developed a functional humanized antibody, mAb146, that cross-reacts with both human and murine CTLA-4. This antibody specifically binds to N110 on CTLA-4, showing greater blockade of ligand binding compared to ipilimumab, underscoring the critical role of CTLA-4 glycosylation in modulating its biological function [307].

However, the development of small-molecule inhibitors directly targeting CTLA-4 PTMs remains limited. Recently, artificial intelligence (AI)-based screening has emerged as a novel approach to address this challenge. Sobhani et al. utilized an AI algorithm based on deep convolutional neural networks, trained to recognize putative binding pockets on CTLA-4. Through this AI-driven approach, several candidate compounds that successfully bind to CTLA-4 and inhibit its interaction with CD80 were identified. These compounds were validated in molecular and cellular assays and had demonstrated significant tumor growth inhibition in CTLA-4 humanized tumor-bearing mice [308].

### LAG-3

Eftilagimod alpha (efti; IMP321) is a recombinant sLAG-3 that functions as a potent MHC class II agonist. By binding to MHC class II molecules, efti exerts immunostimulatory effects by promoting DC maturation and enhancing type 1 T helper responses, which in turn lead to more robust activation of cytotoxic CD8⁺ T cells. In a phase I clinical trial (NCT03252938), twelve patients with metastatic solid tumors were treated with the combination of avelumab and efti. Among these patients, five (42%) achieved a partial response, and the 12-month OS rate reached 75% [309]. Additionally, a phase II study (NCT03625323) evaluated efti in combination with pembrolizumab in patients with metastatic NSCLC resistant to PD-1/PDL1 blockade. This combination improved clinical outcomes, with a reported median OS of 9.9 months [310]. These reports highlight the potential of efti as a potent immunostimulatory agent to overcome resistance to PD-1/PD-L1 therapy.

### VISTA

Therapeutic agents targeting VISTA employ diverse mechanisms to overcome immunosuppression within the TME. Humanized antibodies like BMS767, SG7, and VSTB112 target the histidine-rich C–C′ loop, a critical region for ligand binding (e.g., PSGL-1 and VSIG‑3). Among these, BMS767 exhibits pH-selective binding, preferentially active in the acidic TME, and can enhance IFN-γ production and T cell proliferation [311]. SG7 and VSTB112 block ligand interaction across both acidic and neutral pH; SG7 also restores NFAT signaling in T cells and reduces suppressive PMN-MDSCs in vivo. A BMS767-SG7-VSTB112 antibody cocktail shows synergistic competition with VISTA ligands, reversing T cell suppression more effectively [312, 313]. Clinical-stage agents further expand these approaches. CI‑8993, a fully human IgG1κ monoclonal antibody, binds the C–C′ loop and inhibits VISTA signaling while depleting VISTA⁺ myeloid cells via Fcγ receptor (FcγR)-mediated mechanisms. It has also been radiolabeled to assess receptor occupancy, showing safety and favorable pharmacokinetics in early-phase trials [314, 315]. Similarly, HMBD‑002 employs an Fc-inert IgG4 format to avoid immune toxicity while effectively disrupting VISTA-VSIG3/HVEM interactions and synergizing with anti-PD-1 therapy in preclinical studies [316]. KVA12123, a next-generation IgG1, binds VISTA across both pH environments, blocks interactions with multiple ligands (PSGL-1, VSIG3, VSIG8, LRIG1), and demonstrates strong receptor occupancy and anti-tumor efficacy with minimized FcγR engagement in Phase I/II studies [317]. JNJ-61610588 (Onvatilimab) is a fully human IgG1κ anti‑VISTA antibody that is currently evaluated for safety and efficacy in advanced cancer patients, acting via traditional antibody blockade of VISTA signaling [318].

In the realm of small molecules, CA‑170 is the first-in-class oral dual inhibitor of VISTA and PD‑L1. It targets an allosteric site, disrupting the VISTA–VSIG3 interaction, and has advanced through Phase II studies in solid tumors and Hodgkin lymphoma [319]. Additionally, Chidamide (HBI‑8000), originally an oral HDAC inhibitor, has been found to block VISTA-PSGL-1 binding under acidic pH, enhancing CD8⁺ T cell responses in vivo [212]. Compound S8, a bifunctional small molecule, dually targets PD‑L1 and VISTA with micromolar potency and shows promising in vitro immunomodulatory activity [313]. Lastly, SNS‑101, a pH-selective antibody, is designed to inhibit VISTA only in acidic tumor conditions, augmenting anti-PD-1 efficacy while minimizing systemic immune-related toxicity [320].

Collectively, these VISTA-targeted therapies highlight a broad spectrum of immunomodulatory strategies, from epitope-specific antibody blockade and Fc-engineering to pH-conditioned selectivity and orally bioavailable dual checkpoint inhibition, offering versatile platforms to disrupt VISTA-mediated immune escape in cancer.

### BTLA

Preclinical studies demonstrate that blockade of the BTLA-HVEM interaction, or interference with phosphorylation-mediated SHP recruitment, can restore antitumor T cell activity [321]. Translating this into clinical practice, the humanized anti-BTLA antibody Icatolimab (JS004/TAB004, also named Tifcemalimab) has entered multiple early-phase trials, including NCT04137900, NCT05000684, and NCT04477772, and has demonstrated acceptable safety with preliminary antitumor activity in both solid tumors and hematologic malignancies [322]. Updated trial results confirmed the clinical activity of Icatolimab, particularly when combined with PD-1 inhibitors [323, 324]. In NSCLC and melanoma, dual inhibition of BTLA and PD-1 signaling reinvigorates exhausted T cells and improves tumor control [325].

Beyond Icatolimab, additional strategies are under investigation. Anti-HVEM antibodies can disrupt HVEM-mediated suppression of T-cell responses, while HVEM-Fc fusion proteins have shown promise in lung cancer models as decoys to rewire signaling [326]. Moreover, structure-guided studies of BTLA-binding antibodies (e.g., 22B3, 25F7) have clarified epitope topography and cis-complex regulation, which may inform the rational design of selective BTLA antagonists [327].

Collectively, these findings indicate that targeting the BTLA–HVEM axis—either as monotherapy, through ligand blockade, or in rational combinations—represents a promising strategy to overcome resistance to PD-1/PD-L1 therapies and improve outcomes. Ongoing Phase I/II trials will be critical to define the therapeutic value of BTLA, and provide a strong rationale for combination strategies with PD-1, TIGIT, or CTLA-4 in next-generation checkpoint immunotherapy [323, 328].

### SIRPα

SIRPα, an inhibitory receptor highly expressed on myeloid cells, binds to CD47, a "don't eat me" signal expressed on tumor cells, to inhibit phagocytosis and enable immune evasion [329]. Therapeutic strategies targeting SIRPα have expanded beyond monoclonal antibodies to encompass diverse modalities that reprogram innate immunity within the TME. Among these, oncolytic adenoviruses (OAds) engineered to locally express SIRPα-Fc have shown efficacy in preclinical tumor models by reprogramming TAMs and DCs toward a pro-inflammatory, antigen-presenting phenotype, leading to enhanced CD8⁺ T cell responses and synergy with PD-1 blockade [330].

Similarly, pan-allelic neutralizing antibodies such as ES004‑B5 and KWAR23 effectively block SIRPα-CD47 interactions, promote phagocytosis, and stimulate T cell priming within the TME. Clinical-stage fusion proteins such as BI765063, ALX148 (Evorpacept), TTI-621, and TTI-622 [331–333] incorporate modified SIRPα extracellular domains with attenuated FcγR binding. These agents are under evaluation in hematologic and solid tumors, and have demonstrated the ability to enhance phagocytic clearance, activate adaptive immunity, and overcome myeloid-derived tolerance in both preclinical and early-phase clinical settings.

Beyond protein-based approaches, small-molecule inhibitors are emerging as attractive alternatives. NCGC00138783, identified through high-throughput screening by Miller and associates, acts as a direct SIRPα antagonist by engaging key residues within the CD47-binding interface (Leu30, Gly34, Pro35, Gln52, Lys53, Lys93), thereby disrupting the inhibitory SIRPα–CD47 axis [273]. Two structurally related analogs, NCGC00538430 and NCGC00538419, were also identified and demonstrated comparable antagonistic activity.

Furthermore, SMC18, a dual-pathway small molecule, simultaneously targets SIRPα-CD47 and PD-1-PD‑L1 checkpoints. It has been shown to synergistically activate macrophage phagocytosis and restore CD8⁺ T cell function in vitro, supporting its potential utility in combination immunotherapy [334]. In addition, NSC622608 and MLN4924, a neddylation inhibitor targeting NAE, disrupt the activity of CRLs, inducing cell cycle arrest, DNA damage, and apoptosis [335]. These effects intersect with innate immune signaling and may complement SIRPα blockade strategies, as well as DNA damage and apoptotic pathways that intersect with innate immune responses [270].

Collectively, these therapeutic strategies, including oncolytic vectors, neutralizing antibodies, Fc-engineered fusion proteins, and small-molecule inhibitors, highlight the centrality of SIRPα-mediated immune suppression in cancer and offer versatile avenues for therapeutic intervention.

## Future perspective

PTMs have emerged as universal and dynamic regulators of checkpoints, orchestrating their protein stability, intracellular trafficking, surface localization, and ligand-receptor interactions. Although individual checkpoints such as PD-L1, PD-1, TIM-3, TIGIT, VISTA, CTLA-4, LAG-3, VISTA, BTLA, SIRPα, and others exhibit distinct expression profiles and cellular contexts, they converge upon shared PTM-regulated pathways. Ubiquitination, glycosylation, phosphorylation, methylation, and palmitoylation collectively fine-tune immune receptor homeostasis in ways highly responsive to TME cues.

This mechanistic insight has catalyzed the development of PTM-targeted therapeutics, particularly small-molecule inhibitors and glyco-engineered antibodies, as promising alternatives or complements to conventional ICB (Table 2). Such strategies offer greater spatiotemporal control and compatibility with existing modalities, including CAR-T, metabolic inhibitors, and epigenetic drugs [105]. With the use of small-molecule inhibitors, glyco-engineered antibodies, and epigenetic modulators, it is now possible to apply combinatorial strategies to degrade, relocalize, or silence checkpoints with cell-type specificity [289]. The treatment landscape is rapidly expanding. Furthermore, checkpoint PTMs offer predictive value as biomarkers; profiling patient-specific PTM signatures may guide treatment selection and predict therapeutic resistance or relapse [18, 313, 334].

In recent years, several novel immunoregulatory receptors have garnered attention as potential next-generation checkpoints [336]. Among them, members of the Siglec family (notably Siglec-15, Siglec-7, and Siglec-9) stand out [337]. Siglec-15 has been characterized as a glyco-checkpoint involved in suppressing T cell responses in the TME [338]. Recent reports implicate Siglec-7 in tumor immune escape and correlation with poor prognosis in breast cancer [339, 340]. Although direct evidence for PTM regulation of these receptors is limited, their inherent dependence on glycan interactions makes them strong candidates for modulation by glycosylation, sialylation, or related modifications.

Another emerging candidate is HHLA2, which are overexpressed in RCC and may function as an inhibitory checkpoint. Given the fact that many canonical checkpoints are regulated at the PTM level, exploring whether HHLA2 undergoes phosphorylation, ubiquitination, or glycosylation will be important [341]. Beyond these, molecules such as NKG2A, B7-H6, and Galectin-3 have also been proposed in recent literature to play immunomodulatory roles that may qualify them as checkpoints pending further validation [342, 343].

## Conclusions

This review highlights PTMs as central regulators of immune checkpoint biology and underscores their potential as therapeutic targets. Emerging checkpoints such as Siglec-15, SLAMF7, and HHLA2, though still lacking detailed PTM characterization, represent promising candidates for future investigation. Advancing studies on these molecules and their regulatory modifications will broaden our understanding of checkpoint biology and pave the way for new therapeutic avenues. Looking forward, the integration of advanced proteomic technologies, PTM-specific inhibitors, and well-designed translational trials will be crucial in translating these mechanistic insights into effective therapies. From a clinical perspective, PTM-centered interventions—ranging from small-molecule inhibitors to glyco-engineered antibodies and next-generation biologics—offer promising opportunities to overcome resistance to PD-1/PD-L1 blockade and to deliver durable, personalized cancer immunotherapies.

## Data Availability

No datasets were generated or analysed during the current study.

## Abbreviations

2F-Fuc: 2-Fluoro-L-fucose
ADAM10: A disintegrin and metalloproteinase 10
AI: Artificial intelligence
AMPK: AMP-activated protein kinase
APCs: Antigen-presenting cells
ARIH1: Ariadne-1 homolog 1
B3GNT3: Beta-1,3-N-acetylglucosaminyltransferase 3
B4GALT1: β-1,4-Galactosyltransferase 1
Bat3: HLA-B-associated transcript 3
BTLA: B and T lymphocyte attenuator
β-TrCP: β-Transducin repeat containing E3 ubiquitin protein ligase
CAR-T: Chimeric antigen receptor T cells
c-Cbl: Casitas B lineage lymphoma
ccRCC: Clear cell renal cell carcinoma
CDK4: Cyclin D-cyclin-dependent kinase 4
CEACAM-1: Carcinoembryonic antigen-related cell adhesion molecule 1
CHST8: Carbohydrate sulfotransferase 8
CK2: Casein kinase 2
CRLs: Cullin-RING E3 ligases
CSN5: COP9 signalosome 5
CTLA-4: Cytotoxic T-lymphocyte-associated protein 4
CUL3: Cullin 3
DCs: Dendritic cells
DHHC3: DHHC-type palmitoyltransferase 3
DNA-PKcs: DNA-dependent protein kinase catalytic subunit
DUB: Deubiquitinase
EGF: Epidermal growth factor
EGFR: Epidermal growth factor receptor
ER: Endoplasmic reticulum
ERAD: ER-associated degradation
FBW7: F-box and WD repeat domain-containing 7
FcγR: Fcγ receptor
FGL1: Fibrinogen-like protein 1
GFRFBXO38: F-box only protein 38
GLT1D1: Glycosyltransferase 1 domain-containing 1
GSK3β: Glycogen synthase kinase 3β
HCC: Hepatocellular carcinoma
HDAC2: Histone deacetylase 2
HK2: Hexokinase 2
HMGB1: High mobility group box 1
HNSCC: Head and neck squamous carcinoma cancer
HUWE1: HECT, UBA, and WWE domain protein 1
HVEM: Herpesvirus entry mediator
ICB: Immune checkpoint blockade
Ig: Immunoglobulin
IL-10: Interleukin-10
ILC2: Innate lymphoid cells group 2
ISG15: Interferon-stimulated gene 15
ITIM: Immunoreceptor tyrosine-based inhibitory motif
ITSM: Immunoreceptor tyrosine-based switch motif
ITT: Immunoreceptor tyrosine tail
JAK1: Janus Kinase 1
KEAP1: Kelch-like ECH-associated protein 1
KLHL22: Kelch-like family member 22
LacNAc: Poly-N-acetyllactosamine
LAG-3: Lymphocyte activation gene-3
Lck: Lymphocyte specific protein tyrosine kinase
LKB 1: Liver kinase B1
LPS: Lipopolysaccharide
LRBA: LPS-responsive beige-like anchor protein
LRIG1: Leucine-rich repeats and immunoglobulin like domains 1
LRRK2: Leucine-rich repeat kinase 2
LSECtin: Liver and lymph node sinusoidal endothelial cell C-type lectin
LUBAC: Linear ubiquitin chain assembly complex
MDM2: Mouse double minute 2
MDSCs: Myeloid-derived suppressor cells
MGAT5: Mannoside acetyl-glucosaminyltransferase 5
MIB2: Mind bomb homolog 2
MMP-13: Matrix metallopeptidase 13
NAE: NEDD8-activating enzyme
NK: Natural killer cells
NSCLC: Non-small cell lung cancer
OAds: Oncolytic adenoviruses
OS: Overall survival
PD-1: Programmed cell death-1
OTULIN: OTU deubiquitinase with linear linkage specificity
PD-L1: Programmed death ligand-1
PDAC: Pancreatic ductal adenocarcinoma
pDC: Plasmacytoid dendritic cell
PFS: Progression-free survival
PSGL-1: P-selectin glycoprotein ligand-1
PTM: Post-translational modification
RCC: Renal carcinoma cells
ROS: Reactive oxygen species
SETD7: SET domain containing 7
SGLT2: Sodium-glucose cotransporter 2
SHP-2: Src homology region 2 domain-containing phosphatase-2
SIRPα: Signal regulatory protein alpha
Skp2: S-phase kinase-associated protein 2
sLAG-3: Soluble LAG-3
sPD-L1: Soluble PD-L1
SPOP: Speckle-type POZ protein
sTIM-3: Soluble TIM-3
ST3GAL4: ST3 beta-galactoside alpha-2,3-sialyltransferase 4
STT3A: STT3 oligosaccharyltransferase complex catalytic subunit A
SUMOylation: Small ubiquitin-like modifier modification
TAM: Tumor-associated macrophages
TBK1: TANK-binding kinase 1
TCR: T cell receptor
TIGIT: T cell immunoreceptor with Ig and ITIM domains
TIL: Tumor-infiltrating lymphocytes
TIM-3: T cell immunoglobulin and mucin-domain containing-3
TME: Tumor microenvironment
TMUB1: Transmembrane and ubiquitin-like domain-containing protein 1
TRAF6: TNF receptor-associated factor 6
TRIM28: Tripartite motif containing 28
UFL1: UFM1 specific ligase 1
UFM1: Ubiquitin-fold modifier 1
USP: Ubiquitin‐specific peptidase
USP9X: Ubiquitin-specific peptidase 9, X-linked
VISTA: V-domain immunoglobulin suppressor of T cell activation

## References

[1] Han Y, Liu D, Li L. PD-1/PD-L1 pathway: current researches in cancer. Am J Cancer Res. 2020;10(3):727–42. 32266087 PMC7136921

[2] Riella LV, Paterson AM, Sharpe AH, Chandraker A. Role of the PD-1 pathway in the immune response. Am J Transplant. 2012;12(10):2575–87. 10.1111/j.1600-6143.2012.04224.x. 22900886 10.1111/j.1600-6143.2012.04224.x PMC3784243

[3] Kim GR, Choi JM. Current understanding of cytotoxic T lymphocyte antigen-4 (CTLA-4) signaling in T-cell biology and disease therapy. Mol Cells. 2022;45(8):513–21. 10.14348/molcells.2022.2056. 35950451 10.14348/molcells.2022.2056 PMC9385567

[4] Acharya N, Sabatos-Peyton C, Anderson AC. Tim-3 finds its place in the cancer immunotherapy landscape. J Immunother Cancer. 2020;8(1):e000911. 10.1136/jitc-2020-000911. 32601081 10.1136/jitc-2020-000911 PMC7326247

[5] Ruffo E, Wu RC, Bruno TC, Workman CJ, Vignali DAA. Lymphocyte-activation gene 3 (LAG3): the next immune checkpoint receptor. Semin Immunol. 2019;42:101305. 10.1016/j.smim.2019.101305. 31604537 10.1016/j.smim.2019.101305 PMC6920665

[6] Blando J, Sharma A, Higa MG, Zhao H, Vence L, Yadav SS, et al. Comparison of immune infiltrates in melanoma and pancreatic cancer highlights VISTA as a potential target in pancreatic cancer. Proc Natl Acad Sci U S A. 2019;116(5):1692–7. 30635425 10.1073/pnas.1811067116 PMC6358697

[7] Logtenberg MEW, Scheeren FA, Schumacher TN. The CD47-SIRPα immune checkpoint. Immunity. 2020;52(5):742–52. 32433947 10.1016/j.immuni.2020.04.011 PMC7340539

[8] Murphy TL, Murphy KM. Slow down and survive: enigmatic immunoregulation by BTLA and HVEM. Annu Rev Immunol. 2010;28:389–411. 20307212 10.1146/annurev-immunol-030409-101202

[9] Alsaafeen BH, Ali BR, Elkord E. Resistance mechanisms to immune checkpoint inhibitors: updated insights. Mol Cancer. 2025;24(1):20. 39815294 10.1186/s12943-024-02212-7 PMC11734352

[10] Cai L, Li Y, Tan J, Xu L, Li Y. Targeting LAG-3, TIM-3, and TIGIT for cancer immunotherapy. J Hematol Oncol. 2023;16(1):101. 37670328 10.1186/s13045-023-01499-1 PMC10478462

[11] Koyama S, Akbay EA, Li YY, Herter-Sprie GS, Buczkowski KA, Richards WG, et al. Adaptive resistance to therapeutic PD-1 blockade is associated with upregulation of alternative immune checkpoints. Nat Commun. 2016;7(1):10501. 10.1038/ncomms10501. 26883990 10.1038/ncomms10501 PMC4757784

[12] Limagne E, Richard C, Thibaudin M, Fumet JD, Truntzer C, Lagrange A, et al. Tim-3/galectin-9 pathway and mMDSC control primary and secondary resistances to PD-1 blockade in lung cancer patients. Oncoimmunology. 2019;8(4):e1564505. 10.1080/2162402X.2018.1564505. 30906658 10.1080/2162402X.2018.1564505 PMC6422400

[13] Cillo AR, Cardello C, Shan F, Karapetyan L, Kunning S, Sander C, et al. Blockade of LAG-3 and PD-1 leads to co-expression of cytotoxic and exhaustion gene modules in CD8+ T cells to promote antitumor immunity. Cell. 2024;187(16):4373-4388.e15. 10.1016/j.cell.2024.06.036. 39121849 10.1016/j.cell.2024.06.036 PMC11346583

[14] Tawbi HA, Schadendorf D, Lipson EJ, Ascierto PA, Matamala L, Castillo Gutiérrez E, et al. Relatlimab and Nivolumab versus Nivolumab in untreated advanced melanoma. N Engl J Med. 2022;386(1):24–34. 10.1056/NEJMoa2109970. 34986285 10.1056/NEJMoa2109970 PMC9844513

[15] Drazic A, Myklebust LM, Ree R, Arnesen T. The world of protein acetylation. Biochim Biophys Acta. 2016;1864(10):1372–401. 10.1016/j.bbapap.2016.06.007. 27296530 10.1016/j.bbapap.2016.06.007

[16] Wilkinson KA, Henley JM. Mechanisms, regulation and consequences of protein SUMOylation. Biochem J. 2010;428(2):133–45. 10.1042/BJ20100158. 20462400 10.1042/BJ20100158 PMC3310159

[17] Nguyen HM, Gaikwad S, Oladejo M, Agrawal MY, Srivastava SK, Wood LM. Interferon stimulated gene 15 (ISG15) in cancer: an update. Cancer Lett. 2023;556:216080. 10.1016/j.canlet.2023.216080. 36736853 10.1016/j.canlet.2023.216080

[18] Hu Q, Shi Y, Wang H, Bing L, Xu Z. Post-translational modifications of immune checkpoints: unlocking new potentials in cancer immunotherapy. Exp Hematol Oncol. 2025;14(1):37. 40087690 10.1186/s40164-025-00627-6 PMC11907956

[19] Oh SA, Wu DC, Cheung J, Navarro A, Xiong H, Cubas R, et al. Expression by dendritic cells is a key regulator of T-cell immunity in cancer. Nat Cancer. 2020;1(7):681–91. 35122038 10.1038/s43018-020-0075-x

[20] Sprooten J, Vanmeerbeek I, Datsi A, Govaerts J, Naulaerts S, Laureano RS, et al. Lymph node and tumor-associated PD-L1+ macrophages antagonize dendritic cell vaccines by suppressing CD8+ T cells. Cell Rep Med. 2024;5(1):101377. 10.1016/j.xcrm.2023.101377. 38232703 10.1016/j.xcrm.2023.101377 PMC10829875

[21] Wang L, Guo W, Guo Z, Yu J, Tan J, Simons DL, et al. PD-L1-expressing tumor-associated macrophages are immunostimulatory and associate with good clinical outcome in human breast cancer. Cell Rep Med. 2024;5(2):101420. 10.1016/j.xcrm.2024.101420. 38382468 10.1016/j.xcrm.2024.101420 PMC10897617

[22] Shen M, Wang J, Yu W, Zhang C, Liu M, Wang K, et al. A novel MDSC-induced PD-1-PD-L1+ B-cell subset in breast tumor microenvironment possesses immuno-suppressive properties. Oncoimmunology. 2018;7(4):e1413520. 10.1080/2162402X.2017.1413520. 29632731 10.1080/2162402X.2017.1413520 PMC5889195

[23] Khan AR, Hams E, Floudas A, Sparwasser T, Weaver CT, Fallon PG. PD-L1hi B cells are critical regulators of humoral immunity. Nat Commun. 2015;6(1):5997. 10.1038/ncomms6997. 25609381 10.1038/ncomms6997

[24] Zheng Y, Han L, Chen Z, Li Y, Zhou B, Hu R, et al. PD-L1+CD8+ T cells enrichment in lung cancer exerted regulatory function and tumor-promoting tolerance. iScience. 2022;25(2):103785. 35146396 10.1016/j.isci.2022.103785 PMC8819393

[25] Brochez L, Meireson A, Chevolet I, Sundahl N, Ost P, Kruse V. Challenging PD-L1 expressing cytotoxic T cells as a predictor for response to immunotherapy in melanoma. Nat Commun. 2018;9(1):2921. 10.1038/s41467-018-05047-1. 30050132 10.1038/s41467-018-05047-1 PMC6062523

[26] Li CW, Lim SO, Xia W, Lee HH, Chan LC, Kuo CW, et al. Glycosylation and stabilization of programmed death ligand-1 suppresses T-cell activity. Nat Commun. 2016;7:12632. 27572267 10.1038/ncomms12632 PMC5013604

[27] Wu Y, Zhang C, Liu X, He Z, Shan B, Zeng Q, et al. ARIH1 signaling promotes anti-tumor immunity by targeting PD-L1 for proteasomal degradation. Nat Commun. 2021;12(1):2346. 33879767 10.1038/s41467-021-22467-8 PMC8058344

[28] Dai X, Bu X, Gao Y, Guo J, Hu J, Jiang C, et al. Energy status dictates PD-L1 protein abundance and anti-tumor immunity to enable checkpoint blockade. Mol Cell. 2021;81(11):2317-2331.e6. 10.1016/j.molcel.2021.03.037. 33909988 10.1016/j.molcel.2021.03.037 PMC8178223

[29] Cha JH, Yang WH, Xia W, Wei Y, Chan LC, Lim SO, et al. Metformin promotes antitumor immunity via endoplasmic-reticulum-associated degradation of PD-L1. Mol Cell. 2018;71(4):606-620.e7. 10.1016/j.molcel.2018.07.030. 30118680 10.1016/j.molcel.2018.07.030 PMC6786495

[30] Chan LC, Li CW, Xia W, Hsu JM, Lee HH, Cha JH, et al. IL-6/JAK1 pathway drives PD-L1 Y112 phosphorylation to promote cancer immune evasion. J Clin Invest. 2019;129(8):3324–38. 10.1172/JCI126022. 31305264 10.1172/JCI126022 PMC6668668

[31] Zhao X, Wei Y, Chu YY, Li Y, Hsu JM, Jiang Z, et al. Phosphorylation and stabilization of PD-L1 by CK2 suppresses dendritic cell function. Cancer Res. 2022;82(11):2185–95. 10.1158/0008-5472.CAN-21-2300. 35385574 10.1158/0008-5472.CAN-21-2300 PMC11957750

[32] Sun K, Zhang X, Lao M, He L, Wang S, Yang H, et al. Targeting leucine-rich repeat serine/threonine-protein kinase 2 sensitizes pancreatic ductal adenocarcinoma to anti-PD-L1 immunotherapy. Mol Ther. 2023;31(10):2929–47. 10.1016/j.ymthe.2023.07.021. 37515321 10.1016/j.ymthe.2023.07.021 PMC10556191

[33] Miao Z, Li J, Wang Y, Shi M, Gu X, Zhang X, et al. Hsa_circ_0136666 stimulates gastric cancer progression and tumor immune escape by regulating the miR-375/PRKDC axis and PD-L1 phosphorylation. Mol Cancer. 2023;22(1):205. 38093288 10.1186/s12943-023-01883-y PMC10718020

[34] Zhang J, Bu X, Wang H, Zhu Y, Geng Y, Nihira NT, et al. Cyclin D-CDK4 kinase destabilizes PD-L1 via cullin 3-SPOP to control cancer immune surveillance. Nature. 2018;553(7686):91–5. 29160310 10.1038/nature25015 PMC5754234

[35] Zhang H, Xia Y, Wang F, Luo M, Yang K, Liang S, et al. Aldehyde dehydrogenase 2 mediates alcohol-induced colorectal cancer immune escape through stabilizing PD-L1 expression. Adv Sci. 2021;8(10):2003404. 10.1002/advs.202003404. 10.1002/advs.202003404 PMC8132160 34026438

[36] Zhang Y, Zeng L, Wang M, Yang Z, Zhang H, Gao L, et al. RIG-I promotes immune evasion of colon cancer by modulating PD-L1 ubiquitination. J Immunother Cancer. 2023;11(9):e007313. 10.1136/jitc-2023-007313. 37758653 10.1136/jitc-2023-007313 PMC10537859

[37] Ding L, Chen X, Zhang W, Dai X, Guo H, Pan X, et al. Canagliflozin primes antitumor immunity by triggering PD-L1 degradation in endocytic recycling. J Clin Invest. 2023;133(1):e154754. 10.1172/JCI154754. 36594471 10.1172/JCI154754 PMC9797339

[38] Shi C, Wang Y, Wu M, Chen Y, Liu F, Shen Z, et al. Promoting anti-tumor immunity by targeting TMUB1 to modulate PD-L1 polyubiquitination and glycosylation. Nat Commun. 2022;13(1):6951. 36376293 10.1038/s41467-022-34346-x PMC9663433

[39] Jing W, Wang G, Cui Z, Xiong G, Jiang X, Li Y, et al. FGFR3 destabilizes PD-L1 via NEDD4 to control T-cell-mediated bladder cancer immune surveillance. Cancer Res. 2022;82(1):114–29. 10.1158/0008-5472.CAN-21-2362. 34753771 10.1158/0008-5472.CAN-21-2362

[40] Li J, Shi D, Li S, Shi X, Liu Y, Zhang Y, et al. KEAP1 promotes anti-tumor immunity by inhibiting PD-L1 expression in NSCLC. Cell Death Dis. 2024;15(2):175. 38413563 10.1038/s41419-024-06563-3 PMC10899596

[41] Gao Y, Zou T, Xu P, Wang Y, Jiang Y, Chen YX, et al. Fusobacterium nucleatum stimulates cell proliferation and promotes PD-L1 expression via IFIT1-related signal in colorectal cancer. Neoplasia. 2023;35:100850. 10.1016/j.neo.2022.100850. 36371909 10.1016/j.neo.2022.100850 PMC9664554

[42] Jiang C, He L, Xiao S, Wu W, Zhao Q, Liu F. E3 ubiquitin ligase RNF125 suppresses immune escape in head and neck squamous cell carcinoma by regulating PD-L1 expression. Mol Biotechnol. 2023;65(6):891–903. 36344734 10.1007/s12033-022-00587-w

[43] Yi J, Tavana O, Li H, Wang D, Baer RJ, Gu W. Targeting USP2 regulation of VPRBP-mediated degradation of p53 and PD-L1 for cancer therapy. Nat Commun. 2023;14(1):1941. 37024504 10.1038/s41467-023-37617-3 PMC10079682

[44] Guo W, Ma J, Guo S, Wang H, Wang S, Shi Q, et al. A20 regulates the therapeutic effect of anti-PD-1 immunotherapy in melanoma. J Immunother Cancer. 2020;8(2):e001866. 10.1136/jitc-2020-001866. 33298620 10.1136/jitc-2020-001866 PMC7733187

[45] Mezzadra R, Sun C, Jae LT, Gomez-Eerland R, de Vries E, Wu W, et al. Identification of CMTM6 and CMTM4 as PD-L1 protein regulators. Nature. 2017;549(7670):106–10. 28813410 10.1038/nature23669 PMC6333292

[46] Qian G, Guo J, Vallega KA, Hu C, Chen Z, Deng Y, et al. Membrane-associated RING-CH 8 functions as a novel PD-L1 E3 ligase to mediate PD-L1 degradation induced by EGFR inhibitors. Mol Cancer Res. 2021;19(10):1622–34. 10.1158/1541-7786.MCR-21-0147. 34183449 10.1158/1541-7786.MCR-21-0147 PMC8492505

[47] Yu X, Li W, Liu H, Wang X, Coarfa C, Cheng C, et al. PD-L1 translocation to the plasma membrane enables tumor immune evasion through MIB2 ubiquitination. J Clin Invest. 2023;133(3):e160456. 10.1172/JCI160456. 36719382 10.1172/JCI160456 PMC9888393

[48] Lv L, Miao Q, Zhan S, Chen P, Liu W, Lv J, et al. LKB1 dictates sensitivity to immunotherapy through Skp2-mediated ubiquitination of PD-L1 protein in non-small cell lung cancer. J Immunother Cancer. 2024;12(12):e009444. 10.1136/jitc-2024-009444. 39694700 10.1136/jitc-2024-009444 PMC11660338

[49] Lim SO, Li CW, Xia W, Cha JH, Chan LC, Wu Y, et al. Deubiquitination and stabilization of PD-L1 by CSN5. Cancer Cell. 2016;30(6):925–39. 27866850 10.1016/j.ccell.2016.10.010 PMC5171205

[50] Jingjing W, Wenzheng G, Donghua W, Guangyu H, Aiping Z, Wenjuan W. Deubiquitination and stabilization of programmed cell death ligand 1 by ubiquitin-specific peptidase 9, X-linked in oral squamous cell carcinoma. Cancer Med. 2018;7(8):4004–11. 10.1002/cam4.1675. 29992764 10.1002/cam4.1675 PMC6089178

[51] Wang Y, Sun Q, Mu N, Sun X, Wang Y, Fan S, et al. The deubiquitinase USP22 regulates PD-L1 degradation in human cancer cells. Cell Commun Signal. 2020;18(1):112. 10.1186/s12964-020-00612-y. 32665011 10.1186/s12964-020-00612-y PMC7362500

[52] Wang Z, Kang W, Li O, Qi F, Wang J, You Y, et al. Abrogation of USP7 is an alternative strategy to downregulate PD-L1 and sensitize gastric cancer cells to T cells killing. Acta Pharm Sin B. 2021;11(3):694–707. 10.1016/j.apsb.2020.11.005. 33777676 10.1016/j.apsb.2020.11.005 PMC7982505

[53] Kuang Z, Liu X, Zhang N, Dong J, Sun C, Yin M, et al. USP2 promotes tumor immune evasion via deubiquitination and stabilization of PD-L1. Cell Death Differ. 2023;30(10):2249–64. 10.1038/s41418-023-01219-9. 37670038 10.1038/s41418-023-01219-9 PMC10589324

[54] Zhu D, Xu R, Huang X, Tang Z, Tian Y, Zhang J, et al. Deubiquitinating enzyme OTUB1 promotes cancer cell immunosuppression via preventing ER-associated degradation of immune checkpoint protein PD-L1. Cell Death Differ. 2021;28(6):1773–89. 10.1038/s41418-020-00700-z. 33328570 10.1038/s41418-020-00700-z PMC8184985

[55] Fu M, Li J, Xuan Z, Zheng Z, Liu Y, Zhang Z, et al. NDR1 mediates PD-L1 deubiquitination to promote prostate cancer immune escape via USP10. Cell Commun Signal. 2024;22(1):429. 10.1186/s12964-024-01805-5. 39227807 10.1186/s12964-024-01805-5 PMC11370014

[56] Young MJ, Wang SA, Chen YC, Liu CY, Hsu KC, Tang SW, et al. USP24-i-101 targeting of USP24 activates autophagy to inhibit drug resistance acquired during cancer therapy. Cell Death Differ. 2024;31(5):574–91. 38491202 10.1038/s41418-024-01277-7 PMC11093971

[57] Li CW, Lim SO, Chung EM, Kim YS, Park AH, Yao J, et al. Eradication of triple-negative breast cancer cells by targeting glycosylated PD-L1. Cancer Cell. 2018;33(2):187-201.e10. 29438695 10.1016/j.ccell.2018.01.009 PMC5824730

[58] Hsu JM, Xia W, Hsu YH, Chan LC, Yu WH, Cha JH, et al. STT3-dependent PD-L1 accumulation on cancer stem cells promotes immune evasion. Nat Commun. 2018;9(1):1908. 29765039 10.1038/s41467-018-04313-6 PMC5954021

[59] Wang D, Wu S, He J, Sun L, Zhu H, Zhang Y, et al. FAT4 overexpression promotes antitumor immunity by regulating the β-catenin/STT3/PD-L1 axis in cervical cancer. J Exp Clin Cancer Res. 2023;42(1):222. 37658376 10.1186/s13046-023-02758-2 PMC10472690

[60] Ma XM, Luo YF, Zeng FF, Su C, Liu X, Li XP, et al. TGF-β1-mediated PD-L1 glycosylation contributes to immune escape via c-Jun/STT3A pathway in nasopharyngeal carcinoma. Front Oncol. 2022;12:815437. 10.3389/fonc.2022.815437. 35311117 10.3389/fonc.2022.815437 PMC8930841

[61] Paul S, Das K, Ghosh A, Chatterjee A, Bhoumick A, Basu A, et al. Coagulation factor VIIa enhances programmed death-ligand 1 expression and its stability in breast cancer cells to promote breast cancer immune evasion. J Thromb Haemost. 2023;21(12):3522–38. 10.1016/j.jtha.2023.08.008. 37579880 10.1016/j.jtha.2023.08.008

[62] Liu X, Zhang Y, Han Y, Lu W, Yang J, Tian J, et al. Overexpression of GLT1D1 induces immunosuppression through glycosylation of PD-L1 and predicts poor prognosis in B-cell lymphoma. Mol Oncol. 2020;14(5):1028–44. 10.1002/1878-0261.12664. 32157792 10.1002/1878-0261.12664 PMC7191186

[63] Cui Y, Li J, Zhang P, Yin D, Wang Z, Dai J, et al. B4GALT1 promotes immune escape by regulating the expression of PD-L1 at multiple levels in lung adenocarcinoma. J Exp Clin Cancer Res. 2023;42(1):146. 37303063 10.1186/s13046-023-02711-3 PMC10259029

[64] Chong S, Liu Y, Bian Z, Hu D, Guo S, Dong C, et al. GALNT6 dual regulates innate immunity STING signaling and PD-L1 expression to promote immune evasion in pancreatic ductal adenocarcinoma. Cell Signal. 2025;134:111942. 10.1016/j.cellsig.2025.111942. 40541816 10.1016/j.cellsig.2025.111942

[65] Greco B, Malacarne V, De Girardi F, Scotti GM, Manfredi F, Angelino E, et al. Disrupting N-glycan expression on tumor cells boosts chimeric antigen receptor T cell efficacy against solid malignancies. Sci Transl Med. 2022;14(628):eabg3072. 35044789 10.1126/scitranslmed.abg3072

[66] Zeng K, Zeng Y, Zhan H, Zhan Z, Wang L, Xie Y, et al. SEC61G assists EGFR-amplified glioblastoma to evade immune elimination. Proc Natl Acad Sci U S A. 2023;120(32):e2303400120. 10.1073/pnas.2303400120. 37523556 10.1073/pnas.2303400120 PMC10410745

[67] Duan X, Xie Y, Yu J, Hu X, Liu Z, Li N, et al. MCT4/lactate promotes PD-L1 glycosylation in triple-negative breast cancer cells. J Oncol. 2022;2022:3659714. 10.1155/2022/3659714. 36199799 10.1155/2022/3659714 PMC9529401

[68] Cai K, Chen Q, Shi D, Huang S, Wang C, Ai Z, et al. Sialylation-dependent interaction between PD-L1 and CD169 promotes monocyte adhesion to endothelial cells. Glycobiology. 2023;33(3):215–24. 10.1093/glycob/cwad005. 36651496 10.1093/glycob/cwad005

[69] Singh R, Choi BK. Siglec1-expressing subcapsular sinus macrophages provide soil for melanoma lymph node metastasis. Elife. 2019;8:e48916. 10.7554/eLife.48916. 31872800 10.7554/eLife.48916 PMC6930078

[70] Chou WC, Chen WT, Kuo CT, Chang YM, Lu YS, Li CW, et al. Genetic insights into carbohydrate sulfotransferase 8 and its impact on the immunotherapy efficacy of cancer. Cell Rep. 2024;43(1):113641. 10.1016/j.celrep.2023.113641. 38165805 10.1016/j.celrep.2023.113641

[71] Yao H, Lan J, Li C, Shi H, Brosseau JP, Wang H, et al. Inhibiting PD-L1 palmitoylation enhances T-cell immune responses against tumours. Nat Biomed Eng. 2019;3(4):306–17. 30952982 10.1038/s41551-019-0375-6

[72] Yang Y, Hsu JM, Sun L, Chan LC, Li CW, Hsu JL, et al. Palmitoylation stabilizes PD-L1 to promote breast tumor growth. Cell Res. 2019;29(1):83–6. 30514902 10.1038/s41422-018-0124-5 PMC6318320

[73] Zhang S, Wang HY, Tao X, Chen Z, Levental I, Lin X. Palmitoylation of PD-L1 regulates its membrane orientation and immune evasion. Langmuir. 2025;41(8):5170–8. 39965093 10.1021/acs.langmuir.4c04441

[74] Zhou J, Ma X, He X, Chen B, Yuan J, Jin Z, et al. Dysregulation of PD-L1 by UFMylation imparts tumor immune evasion and identified as a potential therapeutic target. Proc Natl Acad Sci U S A. 2023;120(11):e2215732120. 10.1073/pnas.2215732120. 36893266 10.1073/pnas.2215732120 PMC10089188

[75] Romeo MA, Gilardini Montani MS, Santarelli R, Benedetti R, Arena A, Cirone M. Acetylation increases expression, interaction with TRAPPC4 and surface localization of PD-L1. Discov Oncol. 2023;14(1):152. 10.1007/s12672-023-00766-4. 37603071 10.1007/s12672-023-00766-4 PMC10442048

[76] Gao Y, Nihira NT, Bu X, Chu C, Zhang J, Kolodziejczyk A, et al. Acetylation-dependent regulation of PD-L1 nuclear translocation dictates the efficacy of anti-PD-1 immunotherapy. Nat Cell Biol. 2020;22(9):1064–75. 10.1038/s41556-020-0562-4. 32839551 10.1038/s41556-020-0562-4 PMC7484128

[77] Ma X, Jia S, Wang G, Liang M, Guo T, Du H, et al. TRIM28 promotes the escape of gastric cancer cells from immune surveillance by increasing PD-L1 abundance. Signal Transduct Target Ther. 2023;8(1):246. 10.1038/s41392-023-01450-3. 37357254 10.1038/s41392-023-01450-3 PMC10290989

[78] Huang C, Ren S, Chen Y, Liu A, Wu Q, Jiang T, et al. PD-L1 methylation restricts PD-L1/PD-1 interactions to control cancer immune surveillance. Sci Adv. 2023;9(21):eade4186. 37235656 10.1126/sciadv.ade4186 PMC10219601

[79] Qu T, Zhang W, Yan C, Ren D, Wang Y, Guo Y, et al. ISG15 targets glycosylated PD-L1 and promotes its degradation to enhance antitumor immune effects in lung adenocarcinoma. J Transl Med. 2023;21(1):341. 10.1186/s12967-023-04135-1. 37217923 10.1186/s12967-023-04135-1 PMC10204161

[80] Pitts SC, Schlom J, Donahue RN. Soluble immune checkpoints: implications for cancer prognosis and response to immune checkpoint therapy and conventional therapies. J Exp Clin Cancer Res. 2024;43(1):155. 10.1186/s13046-024-03074-z. 38822401 10.1186/s13046-024-03074-z PMC11141022

[81] Orme JJ, Jazieh KA, Xie T, Harrington S, Liu X, Ball M, et al. ADAM10 and ADAM17 cleave PD-L1 to mediate PD-(L)1 inhibitor resistance. Oncoimmunology. 2020;9(1):1744980. 10.1080/2162402X.2020.1744980. 32363112 10.1080/2162402X.2020.1744980 PMC7185206

[82] Dezutter-Dambuyant C, Durand I, Alberti L, Bendriss-Vermare N, Valladeau-Guilemond J, Duc A, et al. A novel regulation of PD-1 ligands on mesenchymal stromal cells through MMP-mediated proteolytic cleavage. Oncoimmunology. 2015;5(3):e1091146. 10.1080/2162402X.2015.1091146. 27141350 10.1080/2162402X.2015.1091146 PMC4839348

[83] Hira-Miyazawa M, Nakamura H, Hirai M, Kobayashi Y, Kitahara H, Bou-Gharios G, et al. Regulation of programmed-death ligand in the human head and neck squamous cell carcinoma microenvironment is mediated through matrix metalloproteinase-mediated proteolytic cleavage. Int J Oncol. 2018;52(2):379–88. 29345283 10.3892/ijo.2017.4221 PMC5741372

[84] Cheng Y, Wang C, Wang Y, Dai L. Soluble PD-L1 as a predictive biomarker in lung cancer: a systematic review and meta-analysis. Future Oncol. 2022;18(2):261–73. 10.2217/fon-2021-0641. 34874185 10.2217/fon-2021-0641

[85] Oh SY, Kim S, Keam B, Kim TM, Kim DW, Heo DS. Soluble PD-L1 is a predictive and prognostic biomarker in advanced cancer patients who receive immune checkpoint blockade treatment. Sci Rep. 2021;11(1):19712. 10.1038/s41598-021-99311-y. 34611279 10.1038/s41598-021-99311-y PMC8492653

[86] Chivu-Economescu M, Herlea V, Dima S, Sorop A, Pechianu C, Procop A, et al. Soluble PD-L1 as a diagnostic and prognostic biomarker in resectable gastric cancer patients. Gastric Cancer. 2023;26(6):934–46. 37668884 10.1007/s10120-023-01429-7 PMC12316475

[87] He Y, Zhang X, Zhu M, He W, Hua H, Ye F, et al. Soluble PD-L1: a potential dynamic predictive biomarker for immunotherapy in patients with proficient mismatch repair colorectal cancer. J Transl Med. 2023;21(1):25. 36639643 10.1186/s12967-023-03879-0 PMC9837921

[88] Lowther DE, Goods BA, Lucca LE, Lerner BA, Raddassi K, van Dijk D, et al. PD-1 marks dysfunctional regulatory T cells in malignant gliomas. JCI Insight. 2016;1(5):e85935. 10.1172/jci.insight.85935. 27182555 10.1172/jci.insight.85935 PMC4864991

[89] Lim JX, McTaggart T, Jung SK, Smith KJ, Hulme G, Laba S, et al. PD-1 receptor deficiency enhances CD30+ Treg cell function in melanoma. Nat Immunol. 2025;26(7):1074–86. 40457060 10.1038/s41590-025-02172-0 PMC12208868

[90] Kamada T, Togashi Y, Tay C, Ha D, Sasaki A, Nakamura Y, et al. PD-1+ regulatory T cells amplified by PD-1 blockade promote hyperprogression of cancer. Proc Natl Acad Sci U S A. 2019;116(20):9999–10008. 31028147 10.1073/pnas.1822001116 PMC6525547

[91] Gordon SR, Maute RL, Dulken BW, Hutter G, George BM, McCracken MN, et al. PD-1 expression by tumour-associated macrophages inhibits phagocytosis and tumour immunity. Nature. 2017;545(7655):495–9. 10.1038/nature22396. 28514441 10.1038/nature22396 PMC5931375

[92] Lim TS, Chew V, Sieow JL, Goh S, Yeong JP, Soon AL, et al. PD-1 expression on dendritic cells suppresses CD8+ T cell function and antitumor immunity. Oncoimmunology. 2015;5(3):e1085146. 10.1080/2162402X.2015.1085146. 27141339 10.1080/2162402X.2015.1085146 PMC4839350

[93] Farhat M, Croft W, Parry HM, Verma K, Kinsella FAM, Xu J, et al. PD-1 expression contributes to functional impairment of NK cells in patients with B-CLL. Leukemia. 2024;38(8):1813–7. 10.1038/s41375-024-02271-1. 38724674 10.1038/s41375-024-02271-1 PMC11286510

[94] Liu Y, Cheng Y, Xu Y, Wang Z, Du X, Li C, et al. Increased expression of programmed cell death protein 1 on NK cells inhibits NK-cell-mediated anti-tumor function and indicates poor prognosis in digestive cancers. Oncogene. 2017;36(44):6143–53. 10.1038/onc.2017.209. 28692048 10.1038/onc.2017.209 PMC5671935

[95] Xiao X, Lao XM, Chen MM, Liu RX, Wei Y, Ouyang FZ, et al. PD-1hi identifies a novel regulatory B-cell population in human hepatoma that promotes disease progression. Cancer Discov. 2016;6(5):546–59. 26928313 10.1158/2159-8290.CD-15-1408

[96] Patsoukis N, Wang Q, Strauss L, Boussiotis VA. Revisiting the PD-1 pathway. Sci Adv. 2020;6(38):eabd2712. 32948597 10.1126/sciadv.abd2712 PMC7500922

[97] Bardhan K, Aksoylar HI, Le Bourgeois T, Strauss L, Weaver JD, Delcuze B, et al. Phosphorylation of PD-1-Y248 is a marker of PD-1-mediated inhibitory function in human T cells. Sci Rep. 2019;9(1):17252. 10.1038/s41598-019-53463-0. 31754127 10.1038/s41598-019-53463-0 PMC6872651

[98] Bu X, Juneja VR, Reynolds CG, Mahoney KM, Bu MT, McGuire KA, et al. Monitoring PD-1 phosphorylation to evaluate PD-1 signaling during antitumor immune responses. Cancer Immunol Res. 2021;9(12):1465–75. 10.1158/2326-6066.CIR-21-0493. 34635486 10.1158/2326-6066.CIR-21-0493 PMC8642283

[99] Liu J, Wei L, Hu N, Wang D, Ni J, Zhang S, et al. FBW7-mediated ubiquitination and destruction of PD-1 protein primes sensitivity to anti-PD-1 immunotherapy in non-small cell lung cancer. J Immunother Cancer. 2022;10(9):e005116. 10.1136/jitc-2022-005116. 36104103 10.1136/jitc-2022-005116 PMC9476142

[100] Xiao X, Shi J, He C, Bu X, Sun Y, Gao M, et al. ERK and USP5 govern PD-1 homeostasis via deubiquitination to modulate tumor immunotherapy. Nat Commun. 2023;14(1):2859. 10.1038/s41467-023-38605-3. 37208329 10.1038/s41467-023-38605-3 PMC10199079

[101] Meng X, Liu X, Guo X, Jiang S, Chen T, Hu Z, et al. FBXO38 mediates PD-1 ubiquitination and regulates anti-tumour immunity of T cells. Nature. 2018;564(7734):130–5. 30487606 10.1038/s41586-018-0756-0

[102] Zhou XA, Zhou J, Zhao L, Yu G, Zhan J, Shi C, et al. KLHL22 maintains PD-1 homeostasis and prevents excessive T cell suppression. Proc Natl Acad Sci U S A. 2020;117(45):28239–50. 33109719 10.1073/pnas.2004570117 PMC7668036

[103] Lyle C, Richards S, Yasuda K, Napoleon MA, Walker J, Arinze N, et al. c-Cbl targets PD-1 in immune cells for proteasomal degradation and modulates colorectal tumor growth. Sci Rep. 2019;9(1):20257. 31882749 10.1038/s41598-019-56208-1 PMC6934810

[104] Wu Z, Cao Z, Yao H, Yan X, Xu W, Zhang M, et al. Coupled deglycosylation-ubiquitination cascade in regulating PD-1 degradation by MDM2. Cell Rep. 2023;42(7):112693. 10.1016/j.celrep.2023.112693. 37379210 10.1016/j.celrep.2023.112693

[105] Shi J, Zhang Z, Chen HY, Yao Y, Ke S, Yu K, et al. Targeting the TRIM21-PD-1 axis potentiates immune checkpoint blockade and CAR-T cell therapy. Mol Ther. 2025;33(3):1073–90. 10.1016/j.ymthe.2025.01.047. 39905727 10.1016/j.ymthe.2025.01.047 PMC11897759

[106] Hsieh HC, Young MJ, Chen KY, Su WC, Lin CC, Yen YT, et al. Deubiquitinase USP24 activated by IL-6/STAT3 enhances PD-1 protein stability and suppresses T cell antitumor response. Sci Adv. 2025;11(16):eadt4258. 40238877 10.1126/sciadv.adt4258 PMC12002121

[107] Kuo WT, Kuo IY, Hsieh HC, Wu ST, Su WC, Wang YC. Rab37 mediates trafficking and membrane presentation of PD-1 to sustain T cell exhaustion in lung cancer. J Biomed Sci. 2024;31(1):20. 38321486 10.1186/s12929-024-01009-6 PMC10848371

[108] Sun L, Li CW, Chung EM, Yang R, Kim YS, Park AH, et al. Targeting glycosylated PD-1 induces potent antitumor immunity. Cancer Res. 2020;80(11):2298–310. 10.1158/0008-5472.CAN-19-3133. 32156778 10.1158/0008-5472.CAN-19-3133 PMC7272274

[109] Liu K, Tan S, Jin W, Guan J, Wang Q, Sun H, et al. N-glycosylation of PD-1 promotes binding of camrelizumab. EMBO Rep. 2020;21(12):e51444. 10.15252/embr.202051444. 33063473 10.15252/embr.202051444 PMC7726772

[110] Okada M, Chikuma S, Kondo T, Hibino S, Machiyama H, Yokosuka T, et al. Blockage of core fucosylation reduces cell-surface expression of PD-1 and promotes anti-tumor immune responses of T cells. Cell Rep. 2017;20(5):1017–28. 10.1016/j.celrep.2017.07.027. 28768188 10.1016/j.celrep.2017.07.027

[111] Tit-Oon P, Wonglangka A, Boonkanta K, Ruchirawat M, Fuangthong M, Sasisekharan R, et al. Intact mass analysis reveals the novel O-linked glycosylation on the stalk region of PD-1 protein. Sci Rep. 2023;13(1):9631. 37316505 10.1038/s41598-023-36203-3 PMC10267102

[112] Jin J, Zhi X, Wang X, Meng D. Protein palmitoylation and its pathophysiological relevance. J Cell Physiol. 2021;236(5):3220–33. 10.1002/jcp.30122. 33094504 10.1002/jcp.30122

[113] Yao H, Li C, He F, Song T, Brosseau JP, Wang H, et al. A peptidic inhibitor for PD-1 palmitoylation targets its expression and functions. RSC Chem Biol. 2020;2(1):192–205. 34458782 10.1039/d0cb00157k PMC8341464

[114] He C, Xing X, Chen HY, Gao M, Shi J, Xiang B, et al. UFL1 ablation in T cells suppresses PD-1 UFMylation to enhance anti-tumor immunity. Mol Cell. 2024;84(6):1120-1138.e8. 10.1016/j.molcel.2024.01.024. 38377992 10.1016/j.molcel.2024.01.024

[115] Sakuma M, Katagata M, Okayama H, Nakajima S, Saito K, Sato T, et al. TIM-3 expression on dendritic cells in colorectal cancer. Cancers (Basel). 2024;16(10):1888. 10.3390/cancers16101888. 38791963 10.3390/cancers16101888 PMC11120027

[116] Zhu C, Anderson AC, Schubart A, Xiong H, Imitola J, Khoury SJ, et al. The Tim-3 ligand galectin-9 negatively regulates T helper type 1 immunity. Nat Immunol. 2005;6(12):1245–52. 16286920 10.1038/ni1271

[117] Kandel S, Adhikary P, Li G, Cheng K. The TIM3/Gal9 signaling pathway: an emerging target for cancer immunotherapy. Cancer Lett. 2021;10:67–78. 10.1016/j.canlet.2021.04.011 PMC8168453 33895262

[118] Yang R, Sun L, Li CF, Wang YH, Yao J, Li H, et al. Galectin-9 interacts with PD-1 and TIM-3 to regulate T cell death and is a target for cancer immunotherapy. Nat Commun. 2021;12(1):832. 33547304 10.1038/s41467-021-21099-2 PMC7864927

[119] Huang YH, Zhu C, Kondo Y, Anderson AC, Gandhi A, Russell A, et al. CEACAM1 regulates TIM-3-mediated tolerance and exhaustion. Nature. 2015;517(7534):386–90. 25363763 10.1038/nature13848 PMC4297519

[120] Yu S, Ren X, Meng F, Guo X, Tao J, Zhang W, et al. TIM3/CEACAM1 pathway involves in myeloid-derived suppressor cells induced CD8+ T cells exhaustion and bone marrow inflammatory microenvironment in myelodysplastic syndrome. Immunology. 2023;168(2):273–89. 35470423 10.1111/imm.13488

[121] Zhang J, Wang L, Guo H, Kong S, Li W, He Q, et al. The role of Tim-3 blockade in the tumor immune microenvironment beyond T cells. Pharmacol Res. 2024;209:107458. 10.1016/j.phrs.2024.107458. 39396768 10.1016/j.phrs.2024.107458

[122] Chiba S, Baghdadi M, Akiba H, Yoshiyama H, Kinoshita I, Dosaka-Akita H, et al. Tumor-infiltrating DCs suppress nucleic acid-mediated innate immune responses through interactions between the receptor TIM-3 and the alarmin HMGB1. Nat Immunol. 2012;13(9):832–42. 10.1038/ni.2376. 22842346 10.1038/ni.2376 PMC3622453

[123] de Mingo Pulido Á, Hänggi K, Celias DP, Gardner A, Li J, Batista-Bittencourt B, et al. The inhibitory receptor TIM-3 limits activation of the cGAS-STING pathway in intra-tumoral dendritic cells by suppressing extracellular DNA uptake. Immunity. 2021;54(6):1154-1167.e7. 10.1016/j.immuni.2021.04.019. 33979578 10.1016/j.immuni.2021.04.019 PMC8192496

[124] Chen S, Chen J, Kong Y, Li H, Chen Z, Luo L, et al. Knockdown of TIM3 hampers dendritic cell maturation and induces immune suppression by modulating T-cell responses. Int J Mol Sci. 2025;26(9):4332. 10.3390/ijms26094332. 40362568 10.3390/ijms26094332 PMC12072576

[125] Ocaña-Guzman R, Torre-Bouscoulet L, Sada-Ovalle I. TIM-3 regulates distinct functions in macrophages. Front Immunol. 2016;7:229. 10.3389/fimmu.2016.00229. 27379093 10.3389/fimmu.2016.00229 PMC4904032

[126] Katagata M, Okayama H, Nakajima S, Saito K, Sato T, Sakuma M, et al. TIM-3 expression and M2 polarization of macrophages in the TGFβ-activated tumor microenvironment in colorectal cancer. Cancers (Basel). 2023;15(20):4943. 37894310 10.3390/cancers15204943 PMC10605063

[127] Guo Q, Shen S, Guan G, Zhu C, Zou C, Cao J, et al. Cancer cell intrinsic TIM-3 induces glioblastoma progression. iScience. 2022;25(11):105329. 36325060 10.1016/j.isci.2022.105329 PMC9618784

[128] Zheng Y, Li Y, Lian J, Yang H, Li F, Zhao S, et al. TNF-α-induced Tim-3 expression marks the dysfunction of infiltrating natural killer cells in human esophageal cancer. J Transl Med. 2019;17(1):165. 31109341 10.1186/s12967-019-1917-0 PMC6528366

[129] Xu L, Huang Y, Tan L, Yu W, Chen D, Lu C, et al. Increased Tim-3 expression in peripheral NK cells predicts a poorer prognosis and Tim-3 blockade improves NK cell-mediated cytotoxicity in human lung adenocarcinoma. Int Immunopharmacol. 2015;29(2):635–41. 10.1016/j.intimp.2015.09.017. 26428847 10.1016/j.intimp.2015.09.017

[130] Li W, Li F, Zhang X, Lin HK, Xu C. Insights into the post-translational modification and its emerging role in shaping the tumor microenvironment. Signal Transduct Target Ther. 2021;6(1):422. 34924561 10.1038/s41392-021-00825-8 PMC8685280

[131] Lee J, Su EW, Zhu C, Hainline S, Phuah J, Moroco JA, et al. Phosphotyrosine-dependent coupling of Tim-3 to T-cell receptor signaling pathways. Mol Cell Biol. 2011;31(19):3963–74. 10.1128/MCB.05297-11. 21807895 10.1128/MCB.05297-11 PMC3187355

[132] Zhu C, Dixon KO, Newcomer K, Gu G, Xiao S, Zaghouani S, et al. Tim-3 adaptor protein Bat3 is a molecular checkpoint of T cell terminal differentiation and exhaustion. Sci Adv. 2021;7(18):eabd2710. 33931442 10.1126/sciadv.abd2710 PMC8087420

[133] Rangachari M, Zhu C, Sakuishi K, Xiao S, Karman J, Chen A, et al. Bat3 promotes T cell responses and autoimmunity by repressing Tim-3-mediated cell death and exhaustion. Nat Med. 2012;18(9):1394–400. 22863785 10.1038/nm.2871 PMC3491118

[134] LaFleur MW, Nguyen TH, Coxe MA, Miller BC, Yates KB, Gillis JE, et al. PTPN2 regulates the generation of exhausted CD8+ T cell subpopulations and restrains tumor immunity. Nat Immunol. 2019;20(10):1335–47. 31527834 10.1038/s41590-019-0480-4 PMC6754306

[135] Flosbach M, Oberle SG, Scherer S, Zecha J, von Hoesslin M, Wiede F, et al. PTPN2 deficiency enhances programmed T cell expansion and survival capacity of activated T cells. Cell Rep. 2020;32(4):107957. 10.1016/j.celrep.2020.107957. 32726622 10.1016/j.celrep.2020.107957 PMC7408006

[136] Tang R, Acharya N, Subramanian A, Purohit V, Tabaka M, Hou Y, et al. Tim-3 adapter protein Bat3 acts as an endogenous regulator of tolerogenic dendritic cell function. Sci Immunol. 2022;7(69):eabm0631. 35275752 10.1126/sciimmunol.abm0631 PMC9273260

[137] Li C, Fang L, Su X, Zhang J, Xiong H, Yu H, et al. Macrophage miR-4524a-5p/TBP promotes β-TrCP -TIM3 complex activation and TGFβ release and aggravates NAFLD-associated fibrosis. Cell Death Dis. 2025;216(1):315. 10.1038/s41419-025-07574-4 PMC12008196 40251185

[138] Vergoten G, Bailly C. N-glycosylation reinforces interaction of immune checkpoint TIM-3 with a small molecule ligand. Comput Biol Chem. 2023;104:107852. 10.1016/j.compbiolchem.2023.107852. 36965447 10.1016/j.compbiolchem.2023.107852

[139] Holderried TAW, de Vos L, Bawden EG, Vogt TJ, Dietrich J, Zarbl R, et al. Molecular and immune correlates of TIM-3 (HAVCR2) and galectin 9 (LGALS9) mRNA expression and DNA methylation in melanoma. Clin Epigenetics. 2019;11(1):161. 10.1186/s13148-019-0752-8. 31747929 10.1186/s13148-019-0752-8 PMC6868848

[140] Zhang J, Sai K, Wang XL, Ye SQ, Liang LJ, Zhou Y, et al. Tim-3 expression and MGMT methylation status association with survival in glioblastoma. Front Pharmacol. 2020;11:584652. 10.3389/fphar.2020.584652. 33041828 10.3389/fphar.2020.584652 PMC7522578

[141] Zhang L, Tian S, Zhao M, Yang T, Quan S, Yang Q, et al. SUV39H1-DNMT3A-mediated epigenetic regulation of Tim-3 and galectin-9 in the cervical cancer. Cancer Cell Int. 2020;20:325. 32699524 10.1186/s12935-020-01380-y PMC7370487

[142] Zhang L, Tian S, Pei M, Zhao M, Wang L, Jiang Y, et al. Crosstalk between histone modification and DNA methylation orchestrates the epigenetic regulation of the costimulatory factors, Tim‑3 and galectin‑9, in cervical cancer. Oncol Rep. 2019;42(6):2655–69. 31661141 10.3892/or.2019.7388 PMC6859457

[143] Zhang Z, Ren C, Xiao R, Ma S, Liu H, Dou Y, et al. Palmitoylation of TIM-3 promotes immune exhaustion and restrains antitumor immunity. Sci Immunol. 2024;9(101):eadp7302. 39546589 10.1126/sciimmunol.adp7302

[144] Möller-Hackbarth K, Dewitz C, Schweigert O, Trad A, Garbers C, Rose-John S, et al. A disintegrin and metalloprotease (ADAM) 10 and ADAM17 are major sheddases of T cell immunoglobulin and mucin domain 3 (Tim-3). J Biol Chem. 2013;288(48):34529–44. 10.1074/jbc.M113.488478. 24121505 10.1074/jbc.M113.488478 PMC3843067

[145] Chen C, Zhao F, Peng J, Zhao D, Xu L, Li H, et al. Soluble Tim-3 serves as a tumor prognostic marker and therapeutic target for CD8^+^ T cell exhaustion and anti-PD-1 resistance. Cell Rep Med. 2024;5(8):101686. 10.1016/j.xcrm.2024.101686. 39168104 10.1016/j.xcrm.2024.101686 PMC11384939

[146] Pourmir I, Benhamouda N, Tran T, Roux H, Pineau J, Gey A, et al. Soluble TIM-3, likely produced by myeloid cells, predicts resistance to immune checkpoint inhibitors in metastatic clear cell renal cell carcinoma. J Exp Clin Cancer Res. 2025;44(1):54. 10.1186/s13046-025-03293-y. 39953623 10.1186/s13046-025-03293-y PMC11827183

[147] Bailly C, Thuru X, Goossens L, Goossens JF. Soluble TIM-3 as a biomarker of progression and therapeutic response in cancers and other of human diseases. Biochem Pharmacol. 2023;209:115445. 10.1016/j.bcp.2023.115445. 36739094 10.1016/j.bcp.2023.115445

[148] Zizzari IG, Di Filippo A, Scirocchi F, Di Pietro FR, Rahimi H, Ugolini A, et al. Soluble immune checkpoints, gut metabolites and performance status as parameters of response to Nivolumab treatment in NSCLC patients. J Pers Med. 2020;10(4):208. 33158018 10.3390/jpm10040208 PMC7712566

[149] Wang F, Liu S, Liu F, Xu T, Ma J, Liang J, et al. TIGIT immune checkpoint blockade enhances immunity of human peripheral blood NK cells against castration-resistant prostate cancer. Cancer Lett. 2023;568:216300. 10.1016/j.canlet.2023.216300. 37414394 10.1016/j.canlet.2023.216300

[150] Yang J, Wang L, Byrnes JR, Kirkemo LL, Driks H, Belair CD, et al. PVRL2 suppresses antitumor immunity through PVRIG and TIGIT-independent pathways. Cancer Immunol Res. 2024;12(5):575–91. 10.1158/2326-6066.CIR-23-0722. 38588410 10.1158/2326-6066.CIR-23-0722 PMC11063765

[151] Worboys JD, Vowell KN, Hare RK, Ambrose AR, Bertuzzi M, Conner MA, et al. TIGIT can inhibit T cell activation via ligation-induced nanoclusters, independent of CD226 co-stimulation. Nat Commun. 2023;14(1):5016. 10.1038/s41467-023-40755-3. 37596248 10.1038/s41467-023-40755-3 PMC10439114

[152] Chiang EY, Mellman I. TIGIT-CD226-PVR axis: advancing immune checkpoint blockade for cancer immunotherapy. J Immunother Cancer. 2022;10(4):e004711. 10.1136/jitc-2022-004711. 35379739 10.1136/jitc-2022-004711 PMC8981293

[153] Lupo KB, Torregrosa-Allen S, Elzey BD, Utturkar S, Lanman NA, Cohen-Gadol AA, et al. TIGIT contributes to the regulation of 4-1BB and does not define NK cell dysfunction in glioblastoma. iScience. 2023;26(12):108353. 38053639 10.1016/j.isci.2023.108353 PMC10694670

[154] Hasan MF, Croom-Perez TJ, Oyer JL, Dieffenthaller TA, Robles-Carrillo LD, Eloriaga JE, et al. TIGIT expression on activated NK cells correlates with greater anti-tumor activity but promotes functional decline upon lung cancer exposure: implications for adoptive cell therapy and TIGIT-targeted therapies. Cancers (Basel). 2023;15(10):2712. 10.3390/cancers15102712. 37345049 10.3390/cancers15102712 PMC10216728

[155] Zhang P, Liu X, Gu Z, Jiang Z, Zhao S, Song Y, et al. Targeting TIGIT for cancer immunotherapy: recent advances and future directions. Biomark Res. 2024;12(1):7. 10.1186/s40364-023-00543-z. 38229100 10.1186/s40364-023-00543-z PMC10790541

[156] Joller N, Anderson AC, Kuchroo VK. LAG-3, TIM-3, and TIGIT: distinct functions in immune regulation. Immunity. 2024;57(2):206–22. 38354701 10.1016/j.immuni.2024.01.010 PMC10919259

[157] Liu S, Zhang H, Li M, Hu D, Li C, Ge B, et al. Recruitment of Grb2 and SHIP1 by the ITT-like motif of TIGIT suppresses granule polarization and cytotoxicity of NK cells. Cell Death Differ. 2013;20(3):456–64. 23154388 10.1038/cdd.2012.141 PMC3569986

[158] Jin HS, Park Y. Hitting the complexity of the TIGIT-CD96-CD112R-CD226 axis for next-generation cancer immunotherapy. BMB Rep. 2021;54(1):2–11. 33298247 10.5483/BMBRep.2021.54.1.229 PMC7851444

[159] Yeo J, Ko M, Lee DH, Park Y, Jin HS. TIGIT/CD226 axis regulates anti-tumor immunity. Pharmaceuticals (Basel). 2021;14(3):200. 10.3390/ph14030200. 33670993 10.3390/ph14030200 PMC7997242

[160] Ge Z, Zhou G, Campos Carrascosa L, Gausvik E, Boor PPC, Noordam L, et al. TIGIT and PD1 co-blockade restores ex vivo functions of human tumor-infiltrating CD8+ T cells in hepatocellular carcinoma. Cell Mol Gastroenterol Hepatol. 2021;12(2):443–64. 10.1016/j.jcmgh.2021.03.003. 33781741 10.1016/j.jcmgh.2021.03.003 PMC8255944

[161] Banta KL, Xu X, Chitre AS, Au-Yeung A, Takahashi C, O’Gorman WE, et al. Mechanistic convergence of the TIGIT and PD-1 inhibitory pathways necessitates co-blockade to optimize anti-tumor CD8+ T cell responses. Immunity. 2022;55(3):512-526.e9. 35263569 10.1016/j.immuni.2022.02.005 PMC9287124

[162] Liu J, Cheng Y, Zheng M, Yuan B, Wang Z, Li X, et al. Targeting the ubiquitination/deubiquitination process to regulate immune checkpoint pathways. Signal Transduct Target Ther. 2021;6(1):28. 10.1038/s41392-020-00418-x. 33479196 10.1038/s41392-020-00418-x PMC7819986

[163] van den Boomen DJ, Lehner PJ. Identifying the ERAD ubiquitin E3 ligases for viral and cellular targeting of MHC class I. Mol Immunol. 2015;68(2 Pt A):106–11. 26210183 10.1016/j.molimm.2015.07.005 PMC4678111

[164] Molfetta R, Milito ND, Zitti B, Lecce M, Fionda C, Cippitelli M, et al. The ubiquitin-proteasome pathway regulates Nectin2/CD112 expression and impairs NK cell recognition and killing. Eur J Immunol. 2019;49(6):873–83. 10.1002/eji.201847848. 30888046 10.1002/eji.201847848

[165] Lin YX, Hung MC, Hsu JL, Hsu JM. The N-linked glycosylations of TIGIT Asn32 and Asn101 facilitate PVR/TIGIT interaction. Biochem Biophys Res Commun. 2021;562:9–14. 10.1016/j.bbrc.2021.05.034. 34030043 10.1016/j.bbrc.2021.05.034

[166] Miao Y, Zhang C, Yang L, Zeng X, Hu Y, Xue X, et al. The activation of PPARγ enhances Treg responses through up-regulating CD36/CPT1-mediated fatty acid oxidation and subsequent N-glycan branching of TβRII/IL-2Rα. Cell Commun Signal. 2022;20(1):48. 10.1186/s12964-022-00849-9. 35392915 10.1186/s12964-022-00849-9 PMC8991706

[167] Tahara S, Okumura G, Matsuo T, Shibuya A, Shibuya K. Essential role of CD155 glycosylation in functional binding to DNAM-1 on natural killer cells. Int Immunol. 2024;36(6):317–25. 10.1093/intimm/dxae005. 38289706 10.1093/intimm/dxae005

[168] Niebel D, Fröhlich A, Zarbl R, Fietz S, de Vos L, Vogt TJ, et al. DNA methylation regulates TIGIT expression within the melanoma microenvironment, is prognostic for overall survival, and predicts progression-free survival in patients treated with anti-PD-1 immunotherapy. Clin Epigenetics. 2022;14(1):50. 10.1186/s13148-022-01270-2. 35410311 10.1186/s13148-022-01270-2 PMC9004005

[169] de Vos L, Carrillo Cano TM, Zarbl R, Klümper N, Ralser DJ, Franzen A, et al. CTLA4, PD-1, PD-L1, PD-L2, TIM-3, TIGIT, and LAG3 DNA methylation is associated with BAP1-aberrancy, transcriptional activity, and overall survival in uveal melanoma. J Immunother. 2022;45(7):324–34. 10.1097/CJI.0000000000000429. 35862127 10.1097/CJI.0000000000000429

[170] Sasidharan Nair V, Toor SM, Taha RZ, Shaath H, Elkord E. DNA methylation and repressive histones in the promoters of PD-1, CTLA-4, TIM-3, LAG-3, TIGIT, PD-L1, and galectin-9 genes in human colorectal cancer. Clin Epigenetics. 2018;10(1):104. 30081950 10.1186/s13148-018-0539-3 PMC6080402

[171] Zitti B, Molfetta R, Fionda C, Quatrini L, Stabile H, Lecce M, et al. Innate immune activating ligand SUMOylation affects tumor cell recognition by NK cells. Sci Rep. 2017;7(1):10445. 28874810 10.1038/s41598-017-10403-0 PMC5585267

[172] Kumar S, Schoonderwoerd MJA, Kroonen JS, de Graaf IJ, Sluijter M, Ruano D, et al. Targeting pancreatic cancer by TAK-981: a SUMOylation inhibitor that activates the immune system and blocks cancer cell cycle progression in a preclinical model. Gut. 2022;71(11):2266–83. 35074907 10.1136/gutjnl-2021-324834 PMC9554032

[173] Do P, Beckwith KA, Cheney C, Tran M, Beaver L, Griffin BG, et al. Leukemic B cell CTLA-4 suppresses costimulation of T cells. J Immunol. 2019;202(9):2806–16. 10.4049/jimmunol.1801359. 30910862 10.4049/jimmunol.1801359 PMC6478536

[174] Ghorbaninezhad F, Masoumi J, Bakhshivand M, Baghbanzadeh A, Mokhtarzadeh A, Kazemi T, et al. CTLA-4 silencing in dendritic cells loaded with colorectal cancer cell lysate improves autologous T cell responses in vitro. Front Immunol. 2022;13:931316. 10.3389/fimmu.2022.931316. 35979362 10.3389/fimmu.2022.931316 PMC9376327

[175] Linsley PS, Bradshaw J, Greene J, Peach R, Bennett KL, Mittler RS. Intracellular trafficking of CTLA-4 and focal localization towards sites of TCR engagement. Immunity. 1996;4(6):535–43. 8673700 10.1016/s1074-7613(00)80480-x

[176] Krummel MF, Allison JP. CD28 and CTLA-4 have opposing effects on the response of T cells to stimulation. J Exp Med. 1995;182(2):459–65. 10.1084/jem.182.2.459. 7543139 10.1084/jem.182.2.459 PMC2192127

[177] Qureshi OS, Zheng Y, Nakamura K, Attridge K, Manzotti C, Schmidt EM, et al. Trans-endocytosis of CD80 and CD86: a molecular basis for the cell-extrinsic function of CTLA-4. Science. 2011;332(6029):600–3. 21474713 10.1126/science.1202947 PMC3198051

[178] Zappasodi R, Serganova I, Cohen IJ, Maeda M, Shindo M, Senbabaoglu Y, et al. CTLA-4 blockade drives loss of Treg stability in glycolysis-low tumours. Nature. 2021;591(7851):652–8. 33588426 10.1038/s41586-021-03326-4 PMC8057670

[179] Tekguc M, Wing JB, Osaki M, Long J, Sakaguchi S. Treg-expressed CTLA-4 depletes CD80/CD86 by trogocytosis, releasing free PD-L1 on antigen-presenting cells. Proc Natl Acad Sci U S A. 2021;118(30):e2023739118. 10.1073/pnas.2023739118. 34301886 10.1073/pnas.2023739118 PMC8325248

[180] Schildberg FA, Klein SR, Freeman GJ, Sharpe AH. Coinhibitory pathways in the B7-CD28 ligand-receptor family. Immunity. 2016;44(5):955–72. 27192563 10.1016/j.immuni.2016.05.002 PMC4905708

[181] Chuang E, Lee KM, Robbins MD, Duerr JM, Alegre ML, Hambor JE, et al. Regulation of cytotoxic T lymphocyte-associated molecule-4 by Src kinases. J Immunol. 1999;162(3):1270–7. 10.4049/jimmunol.162.3.1270. 9973379

[182] Miyatake S, Nakaseko C, Umemori H, Yamamoto T, Saito T. Src family tyrosine kinases associate with and phosphorylate CTLA-4 (CD152). Biochem Biophys Res Commun. 1998;249(2):444–8. 10.1006/bbrc.1998.9191. 9712716 10.1006/bbrc.1998.9191

[183] Shiratori T, Miyatake S, Ohno H, Nakaseko C, Isono K, Bonifacino JS, et al. Tyrosine phosphorylation controls internalization of CTLA-4 by regulating its interaction with clathrin-associated adaptor complex AP-2. Immunity. 1997;6(5):583–9. 9175836 10.1016/s1074-7613(00)80346-5

[184] Zhang Y, Allison JP. Interaction of CTLA-4 with AP50, a clathrin-coated pit adaptor protein. Proc Natl Acad Sci U S A. 1997;94(17):9273–8. 9256472 10.1073/pnas.94.17.9273 PMC23153

[185] Marengère LE, Waterhouse P, Duncan GS, Mittrücker HW, Feng GS, Mak TW. Regulation of T cell receptor signaling by tyrosine phosphatase SYP association with CTLA-4. Science. 1996;272(5265):1170–3. 8638161 10.1126/science.272.5265.1170

[186] Guntermann C, Alexander DR. CTLA-4 suppresses proximal TCR signaling in resting human CD4(+) T cells by inhibiting ZAP-70 Tyr(319) phosphorylation: a potential role for tyrosine phosphatases. J Immunol. 2002;168(9):4420–9. 10.4049/jimmunol.168.9.4420. 11970985 10.4049/jimmunol.168.9.4420

[187] Chikuma S, Murakami M, Tanaka K, Uede T. Janus kinase 2 is associated with a box 1-like motif and phosphorylates a critical tyrosine residue in the cytoplasmic region of cytotoxic T lymphocyte associated molecule-4. J Cell Biochem. 2000;78(2):241–50. 10842319

[188] Teft WA, Chau TA, Madrenas J. Structure-function analysis of the CTLA-4 interaction with PP2A. BMC Immunol. 2009;10(1):23. 10.1186/1471-2172-10-23. 19405949 10.1186/1471-2172-10-23 PMC2683795

[189] Herrmann A, Lahtz C, Nagao T, Song JY, Chan WC, Lee H, et al. CTLA4 promotes Tyk2-STAT3-dependent B-cell oncogenicity. Cancer Res. 2017;77(18):5118–28. 10.1158/0008-5472.CAN-16-0342. 28716895 10.1158/0008-5472.CAN-16-0342 PMC5600851

[190] Lo B, Zhang K, Lu W, Zheng L, Zhang Q, Kanellopoulou C, et al. Patients with LRBA deficiency show CTLA4 loss and immune dysregulation responsive to abatacept therapy. Science. 2015;349(6246):436–40 (**AUTOIMMUNE DISEASE**). 26206937 10.1126/science.aaa1663

[191] Yu J, Cui J, Zhang X, Xu H, Chen Z, Li Y, et al. The OX40-TRAF6 axis promotes CTLA-4 degradation to augment antitumor CD8+ T-cell immunity. Cell Mol Immunol. 2023;20(12):1445–56. 10.1038/s41423-023-01093-y. 37932534 10.1038/s41423-023-01093-y PMC10687085

[192] Tey PY, Dufner A, Knobeloch KP, Pruneda JN, Clague MJ, Urbé S. Rapid turnover of CTLA4 is associated with a complex architecture of reversible ubiquitylation. J Cell Biol. 2025;224(1):e202312141. 10.1083/jcb.202312141. 39404738 10.1083/jcb.202312141 PMC11486831

[193] Darlington PJ, Kirchhof MG, Criado G, Sondhi J, Madrenas J. Hierarchical regulation of CTLA-4 dimer-based lattice formation and its biological relevance for T cell inactivation. J Immunol. 2005;175(2):996–1004. 10.4049/jimmunol.175.2.996. 16002699 10.4049/jimmunol.175.2.996

[194] Lau KS, Partridge EA, Grigorian A, Silvescu CI, Reinhold VN, Demetriou M, et al. Complex N-glycan number and degree of branching cooperate to regulate cell proliferation and differentiation. Cell. 2007;129(1):123–34. 10.1016/j.cell.2007.01.049. 17418791 10.1016/j.cell.2007.01.049

[195] Zhu L, Guo Q, Guo H, Liu T, Zheng Y, Gu P, et al. Versatile characterization of glycosylation modification in CTLA4-Ig fusion proteins by liquid chromatography-mass spectrometry. MAbs. 2014;6(6):1474–85. 10.4161/mabs.36313. 25484062 10.4161/mabs.36313 PMC4622558

[196] Song Y, Qian Y, Huang Z, Khattak SF, Li ZJ. Computational insights into O-glycosylation in a CTLA4 Fc-fusion protein linker and its impact on protein quality attributes. Comput Struct Biotechnol J. 2020;18:3925–35. 10.1016/j.csbj.2020.11.037. 33335689 10.1016/j.csbj.2020.11.037 PMC7734232

[197] Mariuzza RA, Shahid S, Karade SS. The immune checkpoint receptor LAG3: structure, function, and target for cancer immunotherapy. J Biol Chem. 2024;300(5):107241. 10.1016/j.jbc.2024.107241. 38556085 10.1016/j.jbc.2024.107241 PMC11061240

[198] Aigner-Radakovics K, De Sousa Linhares A, Salzer B, Lehner M, Izadi S, Castilho A, et al. The ligand-dependent suppression of TCR signaling by the immune checkpoint receptor LAG3 depends on the cytoplasmic RRFSALE motif. Sci Signal. 2023;16(805):eadg2610. 37788323 10.1126/scisignal.adg2610

[199] Workman CJ, Dugger KJ, Vignali DA. Cutting edge: molecular analysis of the negative regulatory function of lymphocyte activation gene-3. J Immunol. 2002;169(10):5392–5. 10.4049/jimmunol.169.10.5392. 12421911 10.4049/jimmunol.169.10.5392

[200] Bae J, Lee SJ, Park CG, Lee YS, Chun T. Trafficking of LAG-3 to the surface on activated T cells via its cytoplasmic domain and protein kinase C signaling. J Immunol. 2014;193(6):3101–12. 10.4049/jimmunol.1401025. 25108024 10.4049/jimmunol.1401025

[201] Guy C, Mitrea DM, Chou PC, Temirov J, Vignali KM, Liu X, et al. LAG3 associates with TCR-CD3 complexes and suppresses signaling by driving co-receptor-Lck dissociation. Nat Immunol. 2022;23(5):757–67. 35437325 10.1038/s41590-022-01176-4 PMC9106921

[202] Camisaschi C, De Filippo A, Beretta V, Vergani B, Villa A, Vergani E, et al. Alternative activation of human plasmacytoid DCs in vitro and in melanoma lesions: involvement of LAG-3. J Invest Dermatol. 2014;134(7):1893–902. 10.1038/jid.2014.29. 24441096 10.1038/jid.2014.29

[203] Ge H, Guo N, Liu Y, Lang B, Yin X, Yu X, et al. The inhibitory receptor LAG3 affects NK cell IFN-γ production through glycolysis and the PSAT1/STAT1/IFNG pathway. MBio. 2025;16(6):e0023025. 40298450 10.1128/mbio.00230-25 PMC12153268

[204] Maeda TK, Sugiura D, Okazaki IM, Maruhashi T, Okazaki T. Atypical motifs in the cytoplasmic region of the inhibitory immune co-receptor LAG-3 inhibit T cell activation. J Biol Chem. 2019;294(15):6017–26. 10.1074/jbc.RA119.007455. 30760527 10.1074/jbc.RA119.007455 PMC6463702

[205] Zhou L, Ge Y, Fu Y, Wu B, Zhang Y, Li L, et al. Global screening of LUBAC and OTULIN interacting proteins by human proteome microarray. Front Cell Dev Biol. 2021;9:686395. 10.3389/fcell.2021.686395. 34262903 10.3389/fcell.2021.686395 PMC8274477

[206] Jiang Y, Dai A, Huang Y, Li H, Cui J, Yang H, et al. Ligand-induced ubiquitination unleashes LAG3 immune checkpoint function by hindering membrane sequestration of signaling motifs. Cell. 2025;188(9):2354-2371.e18. 40101708 10.1016/j.cell.2025.02.014

[207] Kouo T, Huang L, Pucsek AB, Cao M, Solt S, Armstrong T, et al. Galectin-3 shapes antitumor immune responses by suppressing CD8+ T cells via LAG-3 and inhibiting expansion of plasmacytoid dendritic cells. Cancer Immunol Res. 2015;3(4):412–23. 10.1158/2326-6066.CIR-14-0150. 25691328 10.1158/2326-6066.CIR-14-0150 PMC4390508

[208] Baixeras E, Huard B, Miossec C, Jitsukawa S, Martin M, Hercend T, et al. Characterization of the lymphocyte activation gene 3-encoded protein. A new ligand for human leukocyte antigen class II antigens. J Exp Med. 1992;176(2):327–37. 1380059 10.1084/jem.176.2.327 PMC2119326

[209] Li N, Workman CJ, Martin SM, Vignali DA. Biochemical analysis of the regulatory T cell protein lymphocyte activation gene-3 (LAG-3; CD223). J Immunol. 2004;173(11):6806–12. 10.4049/jimmunol.173.11.6806. 15557174 10.4049/jimmunol.173.11.6806

[210] Li N, Wang Y, Forbes K, Vignali KM, Heale BS, Saftig P, et al. Metalloproteases regulate T-cell proliferation and effector function via LAG-3. EMBO J. 2007;26(2):494–504. 17245433 10.1038/sj.emboj.7601520 PMC1783452

[211] Andrews LP, Somasundaram A, Moskovitz JM, Szymczak-Workman AL, Liu C, Cillo AR, et al. Resistance to PD1 blockade in the absence of metalloprotease-mediated LAG3 shedding. Sci Immunol. 2020;5(49):eabc2728. 32680952 10.1126/sciimmunol.abc2728 PMC7901539

[212] Guo M, Qi F, Rao Q, Sun J, Du X, Qi Z, et al. Serum LAG-3 predicts outcome and treatment response in hepatocellular carcinoma patients with transarterial chemoembolization. Front Immunol. 2021;12:754961. 10.3389/fimmu.2021.754961. 34691076 10.3389/fimmu.2021.754961 PMC8530014

[213] Wang Q, Zhang J, Tu H, Liang D, Chang DW, Ye Y, et al. Soluble immune checkpoint-related proteins as predictors of tumor recurrence, survival, and T cell phenotypes in clear cell renal cell carcinoma patients. J Immunother Cancer. 2019;7(1):334. 10.1186/s40425-019-0810-y. 31783776 10.1186/s40425-019-0810-y PMC6884764

[214] Li N, Jilisihan B, Wang W, Tang Y, Keyoumu S. Soluble LAG3 acts as a potential prognostic marker of gastric cancer and its positive correlation with CD8+T cell frequency and secretion of IL-12 and INF-γ in peripheral blood. Cancer Biomark. 2018;23(3):341–51. 30223387 10.3233/CBM-181278 PMC13078572

[215] Gorgulho J, Roderburg C, Beier F, Bokemeyer C, Brümmendorf TH, Loosen SH, et al. Soluble lymphocyte activation gene-3 (sLAG3) and CD4/CD8 ratio dynamics as predictive biomarkers in patients undergoing immune checkpoint blockade for solid malignancies. Br J Cancer. 2024;130(6):1013–22. 38233492 10.1038/s41416-023-02558-7 PMC10951205

[216] Botticelli A, Zizzari IG, Scagnoli S, Pomati G, Strigari L, Cirillo A, et al. The role of soluble LAG3 and soluble immune checkpoints profile in advanced head and neck cancer: a pilot study. J Pers Med. 2021;11(7):651. 10.3390/jpm11070651. 34357118 10.3390/jpm11070651 PMC8304359

[217] Zhang RJ, Kim TK. VISTA-mediated immune evasion in cancer. Exp Mol Med. 2024;56(11):2348–56. 10.1038/s12276-024-01336-6. 39482534 10.1038/s12276-024-01336-6 PMC11612309

[218] Lin Y, Choukrani G, Dubbel L, Rockstein L, Freile JA, Qi Y, et al. VISTA drives macrophages towards a pro-tumoral phenotype that promotes cancer cell phagocytosis yet down-regulates T cell responses. Exp Hematol Oncol. 2024;13(1):35. 10.1186/s40164-024-00501-x. 38553748 10.1186/s40164-024-00501-x PMC10979580

[219] Vanmeerbeek I, Naulaerts S, Sprooten J, Laureano RS, Govaerts J, Trotta R, et al. Targeting conserved TIM3+VISTA+ tumor-associated macrophages overcomes resistance to cancer immunotherapy. Sci Adv. 2024;10(29):eadm8660. 39028818 10.1126/sciadv.adm8660 PMC11259173

[220] Niu X, Li B, Luo F, Li W, Zhou X, Zhao W. Vista as a context-dependent immune checkpoint: implications for tumor immunity and autoimmune pathogenesis. Biochim Biophys Acta (BBA). 2025;1880(3):189351. 10.1016/j.bbcan.2025.189351. 10.1016/j.bbcan.2025.189351 40350098

[221] ElTanbouly MA, Zhao Y, Nowak E, Li J, Schaafsma E, Le Mercier I, et al. VISTA is a checkpoint regulator for naïve T cell quiescence and peripheral tolerance. Science. 2020;367(6475):eaay0524. 31949051 10.1126/science.aay0524 PMC7391053

[222] Niu X, Zhao W, Zhou X, Luo F, Xiao Y, Luo T, et al. Chidamide functions as a VISTA/PSGL-1 blocker for cancer immunotherapy. Cancer Immunol Immunother. 2025;74(3):104. 39932560 10.1007/s00262-025-03955-y PMC11813839

[223] Johnston RJ, Su LJ, Pinckney J, Critton D, Boyer E, Krishnakumar A, et al. VISTA is an acidic pH-selective ligand for PSGL-1. Nature. 2019;574(7779):565–70. 31645726 10.1038/s41586-019-1674-5

[224] Ta HM, Roy D, Zhang K, Alban T, Juric I, Dong J, et al. Lrig1 engages ligand VISTA and impairs tumor-specific CD8+ T cell responses. Sci Immunol. 2024;9(95):eadi7418. 38758807 10.1126/sciimmunol.adi7418 PMC11334715

[225] Xu W, Dong J, Zheng Y, Zhou J, Yuan Y, Ta HM, et al. Immune-checkpoint protein VISTA regulates antitumor immunity by controlling myeloid cell-mediated inflammation and immunosuppression. Cancer Immunol Res. 2019;7(9):1497–510. 10.1158/2326-6066.CIR-18-0489. 31340983 10.1158/2326-6066.CIR-18-0489 PMC6726548

[226] Zhang K, Zakeri A, Alban T, Dong J, Ta HM, Zalavadia AH, et al. Vista promotes the metabolism and differentiation of myeloid-derived suppressor cells by STAT3 and polyamine-dependent mechanisms. Cell Rep. 2024;43(1):113661. 10.1016/j.celrep.2023.113661. 38175754 10.1016/j.celrep.2023.113661 PMC10851928

[227] Shekari N, Shanehbandi D, Kazemi T, Zarredar H, Baradaran B, Jalali SA, et al. VISTA and its ligands: the next generation of promising therapeutic targets in immunotherapy. Cancer Cell Int. 2023;23(1):265. 37936192 10.1186/s12935-023-03116-0 PMC10631023

[228] Mehta N, Maddineni S, Mathews II, Andres Parra Sperberg R, Huang PS, Cochran JR. Structure and functional binding epitope of V-domain Ig suppressor of T cell activation. Cell Rep. 2019;28(10):2509-2516.e5. 31484064 10.1016/j.celrep.2019.07.073

[229] Mahoney KM, Freeman GJ. Acidity changes immunology: a new VISTA pathway. Nat Immunol. 2020;21(1):13–6. 31822869 10.1038/s41590-019-0563-2

[230] Cheng Y, Gao WW, Tang HM, Deng JJ, Wong CM, Chan CP, et al. β-TrCP-mediated ubiquitination and degradation of liver-enriched transcription factor CREB-H. Sci Rep. 2016;6:23938. 27029215 10.1038/srep23938 PMC4814919

[231] Shimizu K, Fukushima H, Ogura K, Lien EC, Nihira NT, Zhang J, et al. The SCFβ-TRCP E3 ubiquitin ligase complex targets Lipin1 for ubiquitination and degradation to promote hepatic lipogenesis. Sci Signal. 2017;10(460):eaah4117. 28049764 10.1126/scisignal.aah4117 PMC5215841

[232] Xu Y, Zhu Y, Shi Y, Ye B, Bo L, Tao T. Immune checkpoint VISTA negatively regulates microglia glycolysis and activation via TRIM28-mediated ubiquitination of HK2 in sepsis-associated encephalopathy. Mol Neurobiol. 2025;62(4):4452–65. 39455538 10.1007/s12035-024-04572-z

[233] Emaldi M, Alamillo-Maeso P, Rey-Iborra E, Mosteiro L, Lecumberri D, Pulido R, et al. A functional role for glycosylated B7-H5/VISTA immune checkpoint protein in metastatic clear cell renal cell carcinoma. iScience. 2024;27(9):110587. 39262813 10.1016/j.isci.2024.110587 PMC11388181

[234] Borggrewe M, Grit C, Den Dunnen WFA, Burm SM, Bajramovic JJ, Noelle RJ, et al. VISTA expression by microglia decreases during inflammation and is differentially regulated in CNS diseases. Glia. 2018;66(12):2645–58. 30306644 10.1002/glia.23517 PMC6585704

[235] Abdrabou AM, Ahmed SU, Fan MJ, Duong BTV, Chen K, Lo PY, et al. Identification of VISTA regulators in macrophages mediating cancer cell survival. Sci Adv. 2024;10(48):eadq8122. 39602545 10.1126/sciadv.adq8122 PMC11601207

[236] Wojciechowicz K, Spodzieja M, Wardowska A. The BTLA-HVEM complex-the future of cancer immunotherapy. Eur J Med Chem. 2024;268:116231. 10.1016/j.ejmech.2024.116231. 38387336 10.1016/j.ejmech.2024.116231

[237] Ning Z, Liu K, Xiong H. Roles of BTLA in immunity and immune disorders. Front Immunol. 2021;12:654960. 10.3389/fimmu.2021.654960. 33859648 10.3389/fimmu.2021.654960 PMC8043046

[238] Arifin MZ, Leitner J, Egan D, Waidhofer-Söllner P, Kolch W, Zhernovkov V, et al. BTLA and PD-1 signals attenuate TCR-mediated transcriptomic changes. iScience. 2024;27(7):110253. 39021788 10.1016/j.isci.2024.110253 PMC11253514

[239] Xu X, Hou B, Fulzele A, Masubuchi T, Zhao Y, Wu Z, et al. PD-1 and BTLA regulate T cell signaling differentially and only partially through SHP1 and SHP2. J Cell Biol. 2020;219(6):e201905085. 10.1083/jcb.201905085. 32437509 10.1083/jcb.201905085 PMC7265324

[240] Hwang HJ, Lee JJ, Kang SH, Suh JK, Choi ES, Jang S, et al. The BTLA and PD-1 signaling pathways independently regulate the proliferation and cytotoxicity of human peripheral blood γδ T cells. Immunity Inflamm Dis. 2021;9(1):274–87. 10.1002/iid3.390. 10.1002/iid3.390 PMC7860523 33332777

[241] Antibodies specific to glycosylated BTLA. WO2017096017A1. 2017.

[242] Compaan DM, Gonzalez LC, Tom I, Loyet KM, Eaton D, Hymowitz SG. Attenuating lymphocyte activity: the crystal structure of the BTLA-HVEM complex. J Biol Chem. 2005;280(47):39553–61. 16169851 10.1074/jbc.M507629200

[243] Mkhikian H, Zhou RW, Saryan H, Sánchez CD, Balakrishnan A, Dang J, et al. N-glycan branching regulates BTLA opposite to PD-1 to limit T cell hyperactivity induced by branching deficiency. J Immunol. 2024;213(9):1329–37. 39269653 10.4049/jimmunol.2300568 PMC12091073

[244] Kuncewicz K, Bojko M, Battin C, Karczyńska A, Sieradzan A, Sikorska E, et al. BTLA-derived peptides as inhibitors of BTLA/HVEM complex formation - design, synthesis and biological evaluation. Biomed Pharmacother. 2023;165:115161. 10.1016/j.biopha.2023.115161. 37473684 10.1016/j.biopha.2023.115161

[245] Morrissey MA, Kern N, Vale RD. CD47 ligation repositions the inhibitory receptor SIRPA to suppress integrin activation and phagocytosis. Immunity. 2020;53(2):290-302.e6. 32768386 10.1016/j.immuni.2020.07.008 PMC7453839

[246] Zimarino C, Moody W, Davidson SE, Munir H, Shields JD. Disruption of CD47-SIRPα signaling restores inflammatory function in tumor-associated myeloid-derived suppressor cells. iScience. 2024;27(4):109546. 38577107 10.1016/j.isci.2024.109546 PMC10993187

[247] Chen K, Li Y, Ni J, Yang X, Zhou Y, Pang Y, et al. Identification of a novel subtype of SPP1+ macrophages expressing SIRPα: implications for tumor immune evasion and treatment response prediction. Exp Hematol Oncol. 2024;13(1):119. 10.1186/s40164-024-00587-3. 39696410 10.1186/s40164-024-00587-3 PMC11657677

[248] Huang C, Wang X, Wang Y, Feng Y, Wang X, Chen S, et al. Sirpα on tumor-associated myeloid cells restrains antitumor immunity in colorectal cancer independent of its interaction with CD47. Nat Cancer. 2024;5(3):500–16. 10.1038/s43018-023-00691-z. 38200243 10.1038/s43018-023-00691-z

[249] Bian Z, Shi L, Kidder K, Zen K, Garnett-Benson C, Liu Y. Intratumoral SIRPα-deficient macrophages activate tumor antigen-specific cytotoxic T cells under radiotherapy. Nat Commun. 2021;12(1):3229. 10.1038/s41467-021-23442-z. 34050181 10.1038/s41467-021-23442-z PMC8163884

[250] Sakano Y, Sakano K, Hurrell BP, Shafiei-Jahani P, Kazemi MH, Li X, et al. SIRPα engagement regulates ILC2 effector function and alleviates airway hyperreactivity via modulating energy metabolism. Cell Mol Immunol. 2024;21(10):1158–74. 10.1038/s41423-024-01208-z. 39160226 10.1038/s41423-024-01208-z PMC11442993

[251] Tomiyama T, Itoh S, Iseda N, Toshida K, Kosai-Fujimoto Y, Tomino T, et al. Clinical significance of signal regulatory protein alpha (SIRPα) expression in hepatocellular carcinoma. Ann Surg Oncol. 2023;30(6):3378–89. 10.1245/s10434-022-13058-y. 36641515 10.1245/s10434-022-13058-y

[252] Ji K, Zhang Y, Jiang S, Sun L, Zhang B, Hu D, et al. SIRPα blockade improves the antitumor immunity of radiotherapy in colorectal cancer. Cell Death Discov. 2023;9(1):180. 10.1038/s41420-023-01472-4. 37291116 10.1038/s41420-023-01472-4 PMC10250547

[253] Al-Sudani H, Ni Y, Jones P, Karakilic H, Cui L, Johnson LDS, et al. Targeting CD47-SIRPa axis shows potent preclinical anti-tumor activity as monotherapy and synergizes with PARP inhibition. NPJ Precis Oncol. 2023;7(1):69. 10.1038/s41698-023-00418-4. 37468567 10.1038/s41698-023-00418-4 PMC10356752

[254] Londino JD, Gulick D, Isenberg JS, Mallampalli RK. Cleavage of signal regulatory protein α (SIRPα) enhances inflammatory signaling. J Biol Chem. 2015;290(52):31113–25. 10.1074/jbc.M115.682914. 26534964 10.1074/jbc.M115.682914 PMC4692235

[255] Shi L, Kidder K, Bian Z, Chiang SKT, Ouellette C, Liu Y. SIRPα sequesters SHP-2 to promote IL-4 and IL-13 signaling and the alternative activation of macrophages. Sci Signal. 2021;14(702):eabb3966. 34582250 10.1126/scisignal.abb3966

[256] Shi L, Bian Z, Kidder K, Liang H, Liu Y. Non-Lyn Src family kinases activate SIRPα-SHP-1 to inhibit PI3K-Akt2 and dampen proinflammatory macrophage polarization. J Immunol. 2021;207(5):1419–27. 10.4049/jimmunol.2100266. 34348974 10.4049/jimmunol.2100266 PMC8387419

[257] Qian B, Lu R, Mao S, Chen Y, Yang M, Zhang W, et al. Podocyte SIRPα reduction aggravates lupus nephritis via promoting T cell inflammatory responses. Cell Rep. 2024;43(5):114249. 10.1016/j.celrep.2024.114249. 38758648 10.1016/j.celrep.2024.114249

[258] Xia Y, Zhao Y, Tian J, Yang X, Fan Y, Dong S, et al. SIRPα modulates the podocyte cytoskeleton through influencing the phosphorylation of FAK at tyrosine residue 597. Acta Biochim Biophys Sin (Shanghai). 2024;57(5):782–91. 39552221 10.3724/abbs.2024198 PMC12130709

[259] Kurihara H, Harita Y, Ichimura K, Hattori S, Sakai T. SIRP-alpha-CD47 system functions as an intercellular signal in the renal glomerulus. Am J Physiol Renal Physiol. 2010;299(3):F517–27. 20554646 10.1152/ajprenal.00571.2009

[260] Feng Y, Huang C, Wang Y, Chen J. SIRPα: a key player in innate immunity. Eur J Immunol. 2023;53(11):e2350375. 10.1002/eji.202350375. 37672390 10.1002/eji.202350375

[261] Dai P, Sun Y, Huang Z, Liu YT, Gao M, Liu HM, et al. USP2 inhibition unleashes CD47-restrained phagocytosis and enhances anti-tumor immunity. Nat Commun. 2025;16(1):4564. 40379682 10.1038/s41467-025-59621-5 PMC12084640

[262] Du L, Su Z, Wang S, Meng Y, Xiao F, Xu D, et al. EGFR-induced and c-Src-mediated CD47 phosphorylation inhibits TRIM21-dependent polyubiquitylation and degradation of CD47 to promote tumor immune evasion. Adv Sci. 2023;10(27):e2206380. 10.1002/advs.202206380. 10.1002/advs.202206380 PMC10520678 37541303

[263] Zhang J, Zhang C, Zang R, Chen W, Guo Y, Jiang H, et al. Targeting MAN1B1 potently enhances bladder cancer antitumor immunity via deglycosylation of CD47. Cancer Commun. 2025;45(9):1090–112. 10.1002/cac2.70040. 10.1002/cac2.70040 PMC12479130 40493414

[264] Gou Q, Yan B, Duan Y, Guo Y, Qian J, Shi J, et al. Ubiquitination of CD47 regulates innate anti-tumor immune response. Adv Sci. 2025;12(5):e2412205. 10.1002/advs.202412205. 10.1002/advs.202412205 PMC11792004 39665172

[265] Lee WY, Weber DA, Laur O, Stowell SR, McCall I, Andargachew R, et al. The role of cis dimerization of signal regulatory protein alpha (SIRPalpha) in binding to CD47. J Biol Chem. 2010;285(49):37953–63. 20826801 10.1074/jbc.M110.180018 PMC2992229

[266] Hayes BH, Tsai RK, Dooling LJ, Kadu S, Lee JY, Pantano D, et al. Macrophages show higher levels of engulfment after disruption of cis interactions between CD47 and the checkpoint receptor SIRPα. J Cell Sci. 2020;133(5):jcs237800. 31964705 10.1242/jcs.237800 PMC7064788

[267] Leslie KA, Lekka C, Richardson SJ, Russell MA, Morgan NG. Regulation of STAT1 signaling in human pancreatic β-cells by the lysine deacetylase HDAC6: a new therapeutic opportunity in Type 1 diabetes? Diabetes. 2024;73(9):1473–85. 10.2337/db24-0008. 38869827 10.2337/db24-0008

[268] Gracia-Hernandez M, Yende AS, Gajendran N, Alahmadi Z, Li X, Munoz Z, et al. Targeting HDAC6 improves anti-CD47 immunotherapy. J Exp Clin Cancer Res. 2024;43(1):60. 10.1186/s13046-024-02982-4. 38414061 10.1186/s13046-024-02982-4 PMC10898070

[269] Xu X, Wang Q, Guo K, Xu J, Lu Y, Chen H, et al. CD47 blockade reverses resistance to HDAC inhibitor by liberating anti-tumor capacity of macrophages. J Exp Clin Cancer Res. 2025;44(1):67. 10.1186/s13046-025-03335-5. 39994810 10.1186/s13046-025-03335-5 PMC11849317

[270] Yu H, Luo H, Chang L, Wang S, Geng X, Kang L, et al. The NEDD8-activating enzyme inhibitor MLN4924 reduces ischemic brain injury in mice. Proc Natl Acad Sci U S A. 2022;119(6):e2111896119. 10.1073/pnas.2111896119. 35101976 10.1073/pnas.2111896119 PMC8833173

[271] Li Y, Zhou H, Liu P, Lv D, Shi Y, Tang B, et al. SHP2 deneddylation mediates tumor immunosuppression in colon cancer via the CD47/SIRPα axis. J Clin Invest. 2023;133(4):e162870. 10.1172/JCI162870. 36626230 10.1172/JCI162870 PMC9927946

[272] Archilla-Ortega A, Domuro C, Martin-Liberal J, Muñoz P. Blockade of novel immune checkpoints and new therapeutic combinations to boost antitumor immunity. J Exp Clin Cancer Res. 2022;41(1):62. 10.1186/s13046-022-02264-x. 35164813 10.1186/s13046-022-02264-x PMC8842574

[273] Chen L, Zhao X, Liu X, Ouyang Y, Xu C, Shi Y. Development of small molecule drugs targeting immune checkpoints. Cancer Biol Med. 2024;21(5):382–99. 38727005 10.20892/j.issn.2095-3941.2024.0034 PMC11131045

[274] Lin Z, Huang K, Guo H, Jia M, Sun Q, Chen X, et al. Targeting ZDHHC9 potentiates anti-programmed death-ligand 1 immunotherapy of pancreatic cancer by modifying the tumor microenvironment. Biomed Pharmacother. 2023;161:114567. 10.1016/j.biopha.2023.114567. 36963362 10.1016/j.biopha.2023.114567

[275] Yan Z, Wang C, Wu J, Wang J, Ma T. TIM-3 teams up with PD-1 in cancer immunotherapy: mechanisms and perspectives. Mol Biomed. 2025;6(1):27. 10.1186/s43556-025-00267-6. 40332725 10.1186/s43556-025-00267-6 PMC12058639

[276] Sauer N, Janicka N, Szlasa W, Skinderowicz B, Kołodzińska K, Dwernicka W, et al. TIM-3 as a promising target for cancer immunotherapy in a wide range of tumors. Cancer Immunol Immunother. 2023;72(11):3405–25. 10.1007/s00262-023-03516-1. 37567938 10.1007/s00262-023-03516-1 PMC10576709

[277] Sym023 (anti-TIM-3) in patients with advanced solid tumor malignancies or lymphomas. ClinicalTrials.gov identifier: NCT03489343.

[278] Brunner AM, Esteve J, Porkka K, Knapper S, Traer E, Scholl S, et al. Phase Ib study of sabatolimab (MBG453), a novel immunotherapy targeting TIM-3 antibody, in combination with decitabine or azacitidine in high- or very high-risk myelodysplastic syndromes. Am J Hematol. 2024;99(2):E32–6. 10.1002/ajh.27161. 37994196 10.1002/ajh.27161

[279] Zeidan AM, Komrokji RS, Brunner AM. TIM-3 pathway dysregulation and targeting in cancer. Expert Rev Anticancer Ther. 2021;21(5):523–34. 10.1080/14737140.2021.1865814. 33334180 10.1080/14737140.2021.1865814

[280] Gutierrez ME, Tang SC, Powderly JD 2nd, Balmanoukian AS, Hoyle PE, Dong Z, et al. First-in-human phase I open-label study of the anti-TIM-3 monoclonal antibody INCAGN02390 in patients with select advanced or metastatic solid tumors. Oncologist. 2025;30(7):oyaf144. 40631773 10.1093/oncolo/oyaf144 PMC12238946

[281] Harding JJ, Moreno V, Bang YJ, Hong MH, Patnaik A, Trigo J, et al. Blocking TIM-3 in treatment-refractory advanced solid tumors: a phase Ia/b study of LY3321367 with or without an anti-PD-L1 antibody. Clin Cancer Res. 2021;27(8):2168–78. 10.1158/1078-0432.CCR-20-4405. 33514524 10.1158/1078-0432.CCR-20-4405

[282] Ma S, Tian Y, Peng J, Chen C, Peng X, Zhao F, et al. Identification of a small-molecule Tim-3 inhibitor to potentiate T cell-mediated antitumor immunotherapy in preclinical mouse models. Sci Transl Med. 2023;15(722):eadg6752. 37967204 10.1126/scitranslmed.adg6752

[283] Curigliano G, Gelderblom H, Mach N, Doi T, Tai D, Forde PM, et al. Phase I/Ib clinical trial of Sabatolimab, an anti-TIM-3 antibody, alone and in combination with Spartalizumab, an anti-PD-1 antibody, in advanced solid tumors. Clin Cancer Res. 2021;27(13):3620–9. 33883177 10.1158/1078-0432.CCR-20-4746

[284] Ghasemi K. Tiragolumab and TIGIT: pioneering the next era of cancer immunotherapy. Front Pharmacol. 2025;16:1568664. 10.3389/fphar.2025.1568664. 40567374 10.3389/fphar.2025.1568664 PMC12187662

[285] Sun J, Zhang X, Xue L, Cheng L, Zhang J, Chen X, et al. Structural insights into the unique pH-responsive characteristics of the anti-TIGIT therapeutic antibody Ociperlimab. Structure. 2024;32(5):550-561.e5. 10.1016/j.str.2024.02.009. 38460520 10.1016/j.str.2024.02.009

[286] Son W, Lee Y, Park Y, Park KS, Kim S, Youn H, et al. Fc-competent TIGITx4-1BB bispecific antibody exerts potent long-lasting antitumor activity by potentiating CD8+ T cell activity and Fcγ receptor-mediated modulation of the tumor microenvironment. J Immunother Cancer. 2025;2:e010728. 10.1136/jitc-2024-010728 PMC12083285 40010766

[287] Guan X, Hu R, Choi Y, Srivats S, Nabet BY, Silva J, et al. Anti-TIGIT antibody improves PD-L1 blockade through myeloid and Treg cells. Nature. 2024;627(8004):646–55. 38418879 10.1038/s41586-024-07121-9 PMC11139643

[288] Srikanth G, Beda DP, Dwivedi AR, Duddukuri NK, Nanduri S, Patel J. Promising new anti-TIGIT agents: stealthy allies in cancer immunotherapy. Clin Transl Sci. 2025;4:e70212. 10.1111/cts.70212 PMC12013639 40261799

[289] Fuchs N, Zhang L, Calvo-Barreiro L, Kuncewicz K, Gabr M. Inhibitors of immune checkpoints: small molecule-and peptide-based approaches. J Pers Med. 2024;14(1):68. 38248769 10.3390/jpm14010068 PMC10817355

[290] Zhou X, Du J, Wang H, Chen C, Jiao L, Cheng X, et al. Repositioning liothyronine for cancer immunotherapy by blocking the interaction of immune checkpoint TIGIT/PVR. Cell Commun Signal. 2020;18(1):142. 32894141 10.1186/s12964-020-00638-2 PMC7487564

[291] Zhou X, Li Y, Zhang X, Li B, Jin S, Wu M, et al. Hemin blocks TIGIT/PVR interaction and induces ferroptosis to elicit synergistic effects of cancer immunotherapy. Sci China Life Sci. 2024;67(5):996–1009. 38324132 10.1007/s11427-023-2472-4

[292] Zhou X, Jiao L, Qian Y, Dong Q, Sun Y, Zheng WV, et al. Repositioning azelnidipine as a dual inhibitor targeting CD47/SIRPα and TIGIT/PVR pathways for cancer immuno-therapy. Biomolecules. 2021;11(5):706. 34068552 10.3390/biom11050706 PMC8150775

[293] Shaw G, Cavalcante L, Giles FJ, Taylor A. Elraglusib (9-ING-41), a selective small-molecule inhibitor of glycogen synthase kinase-3 beta, reduces expression of immune checkpoint molecules PD-1, TIGIT and LAG-3 and enhances CD8+ T cell cytolytic killing of melanoma cells. J Hematol Oncol. 2022;15(1):134. 36104795 10.1186/s13045-022-01352-x PMC9472445

[294] Li Y, Li B, Wang Q, Zhang X, Zhang Q, Zhou X, et al. Dual targeting of TIGIT and PD-1 with a novel small molecule for cancer immunotherapy. Biochem Pharmacol. 2024;223:116162. 38527557 10.1016/j.bcp.2024.116162

[295] Qiu D, Liu X, Wang W, Jiang X, Wu X, Zheng J, et al. TIGIT axis: novel immune checkpoints in anti-leukemia immunity. Clin Exp Med. 2023;23(2):165–74. 35419661 10.1007/s10238-022-00817-0

[296] Xiong F, Yu M, Xu H, Zhong Z, Li Z, Guo Y, et al. Discovery of TIGIT inhibitors based on DEL and machine learning. Front Chem. 2022;10:982539. 35958238 10.3389/fchem.2022.982539 PMC9360614

[297] Zhou X, Zuo C, Li W, Shi W, Zhou X, Wang H, et al. A novel d-peptide identified by mirror-image phage display blocks TIGIT/PVR for cancer immunotherapy. Angew Chem Int Ed Engl. 2020;59(35):15114–8. 32386245 10.1002/anie.202002783

[298] Hsiehchen D, Kainthla R, Kline H, Siglinsky E, Ahn C, Zhu H. Dual TIGIT and PD-1 blockade with domvanalimab plus zimberelimab in hepatocellular carcinoma refractory to anti-PD-1 therapies: the phase 2 LIVERTI trial. Nat Commun. 2025;16(1):5819. 10.1038/s41467-025-60757-7. 40592848 10.1038/s41467-025-60757-7 PMC12219336

[299] Shapira-Frommer R, Niu J, Perets R, Peters S, Shouse G, Lugowska I, et al. The KEYVIBE program: vibostolimab and pembrolizumab for the treatment of advanced malignancies. Future Oncol. 2024;20(27):1983–91. 10.1080/14796694.2024.2343272. 39230120 10.1080/14796694.2024.2343272 PMC11497960

[300] Mohammed A, Tang B, Sadikot S, Barmaimon G. Acute eosinophilic pneumonia induced by immune checkpoint inhibitor and anti-TIGIT therapy. Am J Case Rep. 2024;25:e943740. 10.12659/AJCR.943740. 38970243 10.12659/AJCR.943740 PMC11322792

[301] Hansen K, Kumar S, Logronio K, Whelan S, Qurashi S, Cheng HY, et al. COM902, a novel therapeutic antibody targeting TIGIT augments anti-tumor T cell function in combination with PVRIG or PD-1 pathway blockade. Cancer Immunol Immunother. 2021;70(12):3525–40. 10.1007/s00262-021-02921-8. 33903974 10.1007/s00262-021-02921-8 PMC10992303

[302] Diong SJ, Jashnani A, Drake AW, Bee C, Findeisen F, Dollinger G, et al. Biophysical characterization of PVR family interactions and therapeutic antibody recognition to TIGIT. MAbs. 2023;15(1):2253788. 10.1080/19420862.2023.2253788. 37675979 10.1080/19420862.2023.2253788 PMC10486284

[303] Shirasuna K, Koelsch G, Seidel-Dugan C, Salmeron A, Steiner P, Winston WM, et al. Characterization of ASP8374, a fully-human, antagonistic anti-TIGIT monoclonal antibody. Cancer Treat Res Commun. 2021;28:100433. 10.1016/j.ctarc.2021.100433. 34273876 10.1016/j.ctarc.2021.100433

[304] Wang Y, Zhang H, Liu C, Wang Z, Wu W, Zhang N, et al. Immune checkpoint modulators in cancer immunotherapy: recent advances and emerging concepts. J Hematol Oncol. 2022;15(1):111. 10.1186/s13045-022-01325-0. 35978433 10.1186/s13045-022-01325-0 PMC9386972

[305] Mettu NB, Ulahannan SV, Bendell JC, Garrido-Laguna I, Strickler JH, Moore KN, et al. A phase 1a/b open-label, dose-escalation study of Etigilimab alone or in combination with Nivolumab in patients with locally advanced or metastatic solid tumors. Clin Cancer Res. 2022;28(5):882–92. 10.1158/1078-0432.CCR-21-2780. 34844977 10.1158/1078-0432.CCR-21-2780

[306] Naing A, McKean M, Tolcher A, Victor A, Hu P, Gao W, et al. TIGIT inhibitor M6223 as monotherapy or in combination with bintrafusp alfa in patients with advanced solid tumors: a first-in-human, phase 1, dose-escalation trial. J Immunother Cancer. 2025;13(2):e010584. 10.1136/jitc-2024-010584. 39929671 10.1136/jitc-2024-010584 PMC11815413

[307] Li D, Li J, Chu H, Wang Z. A functional antibody cross-reactive to both human and murine cytotoxic T-lymphocyte-associated protein 4 via binding to an N-glycosylation epitope. MAbs. 2020;12(1):1725365. 32054416 10.1080/19420862.2020.1725365 PMC7039627

[308] Sobhani N, Tardiel-Cyril DR, Chai D, Generali D, Li JR, Vazquez-Perez J, et al. Artificial intelligence-powered discovery of small molecules inhibiting CTLA-4 in cancer. BJC Rep. 2024;2:4. 38312352 10.1038/s44276-023-00035-5 PMC10838660

[309] Al-Batran SE, Mueller DW, Rafiyan MR, Kiselicki D, Atmaca A, Habibzada T, et al. A soluble LAG-3 protein (eftilagimod alpha) and an anti-PD-L1 antibody (avelumab) tested in a phase I trial: a new combination in immuno-oncology. ESMO Open. 2023;8(5):101623. 10.1016/j.esmoop.2023.101623. 37742484 10.1016/j.esmoop.2023.101623 PMC10594027

[310] Krebs MG, Forster M, Majem M, Peguero J, Iams W, Clay T, et al. Eftilagimod alpha (a soluble LAG-3 protein) combined with pembrolizumab in second-line metastatic NSCLC refractory to anti-programmed cell death protein 1/programmed death-ligand 1-based therapy: final results from a phase 2 study. JTO Clin Res Rep. 2024;5(11):100725. 10.1016/j.jtocrr.2024.100725. 39403626 10.1016/j.jtocrr.2024.100725 PMC11472608

[311] Noelle RJ, Lines JL, Lewis LD, Martell RE, Guillaudeux T, Lee SW, et al. Clinical and research updates on the VISTA immune checkpoint: immuno-oncology themes and highlights. Front Oncol. 2023;13:1225081. 37795437 10.3389/fonc.2023.1225081 PMC10547146

[312] Mehta N, Maddineni S, Kelly RL, Lee RB, Hunter SA, Silberstein JL, et al. An engineered antibody binds a distinct epitope and is a potent inhibitor of murine and human VISTA. Sci Rep. 2020;10(1):15171. 32938950 10.1038/s41598-020-71519-4 PMC7494997

[313] Xiao Y, Shi Y, Shao C, Tang W, Liu H, Chen J, et al. Discovery of bifunctional small molecules targeting PD-L1/VISTA with favorable pharmacokinetics for cancer immunotherapy. Bioorg Chem. 2025;157:108323. 10.1016/j.bioorg.2025.108323. 40049048 10.1016/j.bioorg.2025.108323

[314] Burvenich IJG, Wichmann CW, McDonald AF, Guo N, Rigopoulos A, Huynh N, et al. Targeting of immune checkpoint regulator V-domain Ig suppressor of T-cell activation (VISTA) with 89Zr-labelled CI-8993. Eur J Nucl Med Mol Imaging. 2024;51(13):3863–73. 39060374 10.1007/s00259-024-06854-z PMC11527895

[315] Yum JI, Hong YK. Terminating cancer by blocking VISTA as a novel immunotherapy: hasta la vista, baby. Front Oncol. 2021;11:658488. 33937071 10.3389/fonc.2021.658488 PMC8085549

[316] Thakkar D, Paliwal S, Dharmadhikari B, Guan S, Liu L, Kar S, et al. Rationally targeted anti-VISTA antibody that blockades the C-C’ loop region can reverse VISTA immune suppression and remodel the immune microenvironment to potently inhibit tumor growth in an Fc independent manner. J Immunother Cancer. 2022;10(2):e003382. 10.1136/jitc-2021-003382. 35131861 10.1136/jitc-2021-003382 PMC8823246

[317] Iadonato S, Ovechkina Y, Lustig K, Cross J, Eyde N, Frazier E, et al. A highly potent anti-VISTA antibody KVA12123 - a new immune checkpoint inhibitor and a promising therapy against poorly immunogenic tumors. Front Immunol. 2023;14:1311658. 10.3389/fimmu.2023.1311658. 38152397 10.3389/fimmu.2023.1311658 PMC10751915

[318] Chmiel P, Gęca K, Michalski A, Kłosińska M, Kaczyńska A, Polkowski WP, et al. Vista of the future: novel immunotherapy based on the human V-Set immunoregulatory receptor for digestive system tumors. Int J Mol Sci. 2023;24(12):9945. 10.3390/ijms24129945. 37373091 10.3390/ijms24129945 PMC10297928

[319] Sasikumar PG, Sudarshan NS, Adurthi S, Ramachandra RK, Samiulla DS, Lakshminarasimhan A, et al. PD-1 derived CA-170 is an oral immune checkpoint inhibitor that exhibits preclinical anti-tumor efficacy. Commun Biol. 2021;4(1):699. 10.1038/s42003-021-02191-1. 34103659 10.1038/s42003-021-02191-1 PMC8187357

[320] Thisted T, Smith FD, Mukherjee A, Kleschenko Y, Feng F, Jiang ZG, et al. VISTA checkpoint inhibition by pH-selective antibody SNS-101 with optimized safety and pharmacokinetic profiles enhances PD-1 response. Nat Commun. 2024;15(1):2917. 10.1038/s41467-024-47256-x. 38575562 10.1038/s41467-024-47256-x PMC10995192

[321] Andrzejczak A, Karabon L. BTLA biology in cancer: from bench discoveries to clinical potentials. Biomark Res. 2024;12(1):8. 10.1186/s40364-024-00556-2. 38233898 10.1186/s40364-024-00556-2 PMC10795259

[322] Chen YL, Lin HW, Chien CL, Lai YL, Sun WZ, Chen CA, et al. BTLA blockade enhances cancer therapy by inhibiting IL-6/IL-10-induced CD19high B lymphocytes. J Immunother Cancer. 2019;7(1):313. 10.1186/s40425-019-0744-4. 31753019 10.1186/s40425-019-0744-4 PMC6868712

[323] Sordo-Bahamonde C, Lorenzo-Herrero S, Granda-Díaz R, Martínez-Pérez A, Aguilar-García C, Rodrigo JP, et al. Beyond the anti-PD-1/PD-L1 era: promising role of the BTLA/HVEM axis as a future target for cancer immunotherapy. Mol Cancer. 2023;22(1):142. 37649037 10.1186/s12943-023-01845-4 PMC10466776

[324] Sordo-Bahamonde C, Lorenzo-Herrero S, Martínez-Pérez A, Gonzalez-Rodriguez AP, Payer ÁR, González-García E, et al. BTLA dysregulation correlates with poor outcome and diminished T cell-mediated antitumor responses in chronic lymphocytic leukemia. Cancer Immunol Immunother. 2023;72(7):2529–39. 10.1007/s00262-023-03435-1. 37041226 10.1007/s00262-023-03435-1 PMC10264494

[325] Zhang Y, Yang Y, Zeng Y, Qu Q, Shen D, Mu C, et al. B and T lymphocyte attenuator (BTLA) and PD-1 pathway dual blockade promotes antitumor immune responses by reversing CD8+ T-cell exhaustion in non-small cell lung cancer. Front Immunol. 2025;16:1553042. 10.3389/fimmu.2025.1553042. 40463377 10.3389/fimmu.2025.1553042 PMC12129974

[326] Choi J, Medikonda R, Saleh L, Kim T, Pant A, Srivastava S, et al. Combination checkpoint therapy with anti-PD-1 and anti-BTLA results in a synergistic therapeutic effect against murine glioblastoma. Oncoimmunology. 2021;10(1):1956142. 34484870 10.1080/2162402X.2021.1956142 PMC8409779

[327] Wojciechowicz K, Kuncewicz K, Rutkowski J, Jassem J, Rodziewicz-Motowidło S, Wardowska A, et al. Targeting BTLA with the peptide inhibitor HVEM(14-39) - a new way to restore the activity of T cells in melanoma. Biomed Pharmacother. 2024;175:116675. 10.1016/j.biopha.2024.116675. 38733770 10.1016/j.biopha.2024.116675

[328] Guruprasad P, Carturan A, Zhang Y, Cho JH, Kumashie KG, et al. The BTLA-HVEM axis restricts CAR T cell efficacy in cancer. Nat Immunol. 2024;25(6):1020–32. 38831106 10.1038/s41590-024-01847-4

[329] Li Z, Li Y, Gao J, Fu Y, Hua P, Jing Y, et al. The role of CD47-SIRPα immune checkpoint in tumor immune evasion and innate immunotherapy. Life Sci. 2021;273:119150. 10.1016/j.lfs.2021.119150. 33662426 10.1016/j.lfs.2021.119150

[330] Yang Q, Shu Y, Chen Y, Qi Z, Hu S, Zhang Y, et al. Expression of SIRPα-Fc by oncolytic virus enhances antitumor efficacy through tumor microenvironment reprogramming. Front Immunol. 2025;16:1513555. 40070841 10.3389/fimmu.2025.1513555 PMC11893986

[331] Niu X, Wang C, Jiang H, Gao R, Lu Y, Guo X, et al. A pan-allelic human SIRPα-blocking antibody, ES004-B5, promotes tumor killing by enhancing macrophage phagocytosis and subsequently inducing an effective T-cell response. Antib Ther. 2024;7(3):266–80. 10.1093/abt/tbae022. 39257438 10.1093/abt/tbae022 PMC11384143

[332] Liu J, Xavy S, Mihardja S, Chen S, Sompalli K, Feng D, et al. Targeting macrophage checkpoint inhibitor SIRPα for anticancer therapy. JCI Insight. 2020;5(12):e134728. 10.1172/jci.insight.134728. 32427583 10.1172/jci.insight.134728 PMC7406266

[333] Pelcovits A, Ollila TA, Olszewski AJ. Advances in immunotherapy for the treatment of cutaneous T-cell lymphoma. Cancer Manag Res. 2023;15:989–98. 37700809 10.2147/CMAR.S330908 PMC10493109

[334] Jin S, Wang H, Li Y, Yang J, Li B, Shi P, et al. Discovery of a novel small molecule as CD47/SIRPα and PD-1/PD-L1 dual inhibitor for cancer immunotherapy. Cell Commun Signal. 2024;22(1):173. 10.1186/s12964-024-01555-4. 38462636 10.1186/s12964-024-01555-4 PMC10926604

[335] Liu D, Che X, Wu G. Deciphering the role of neddylation in tumor microenvironment modulation: common outcome of multiple signaling pathways. Biomark Res. 2024;12(1):5. 10.1186/s40364-023-00545-x. 38191508 10.1186/s40364-023-00545-x PMC10773064

[336] Liao X, Zhang D. HHLA2 immune checkpoint is a novel prognostic predictor in hepatocellular carcinoma. Am J Clin Pathol. 2022;158(1):62–9. 10.1093/ajcp/aqab221. 35084443 10.1093/ajcp/aqab221

[337] Stanczak MA, Läubli H. Siglec receptors as new immune checkpoints in cancer. Mol Aspects Med. 2023;90:101112. 10.1016/j.mam.2022.101112. 35948467 10.1016/j.mam.2022.101112

[338] Wang J, Sun J, Liu LN, Flies DB, Nie X, Toki M, et al. Siglec-15 as an immune suppressor and potential target for normalization cancer immunotherapy. Nat Med. 2019;25(4):656–66. 10.1038/s41591-019-0374-x. 30833750 10.1038/s41591-019-0374-x PMC7175920

[339] Benthami H, Zohair B, Rezouki I, Naji O, Miyara K, Ennachit S, et al. Elevated Siglec-7 expression correlates with adverse clinicopathological, immunological, and therapeutic response signatures in breast cancer patients. Front Immunol. 2025;16:1573365. 10.3389/fimmu.2025.1573365. 40547037 10.3389/fimmu.2025.1573365 PMC12179189

[340] Bordoloi D, Kulkarni AJ, Adeniji OS, Pampena MB, Bhojnagarwala PS, Zhao S, et al. Siglec-7 glyco-immune binding mAbs or NK cell engager biologics induce potent antitumor immunity against ovarian cancers. Sci Adv. 2023;9(44):eadh4379. 37910620 10.1126/sciadv.adh4379 PMC10619929

[341] Kula A, Koszewska D, Kot A, Dawidowicz M, Mielcarska S, Waniczek D, et al. The importance of HHLA2 in solid tumors-a review of the literature. Cells. 2024;13(10):794. 10.3390/cells13100794. 38786018 10.3390/cells13100794 PMC11119147

[342] Patwekar M, Sehar N, Patwekar F, Medikeri A, Ali S, Aldossri RM, et al. Novel immune checkpoint targets: a promising therapy for cancer treatments. Int Immunopharmacol. 2024;126:111186. 10.1016/j.intimp.2023.111186. 37979454 10.1016/j.intimp.2023.111186

[343] Scafetta G, D’Alessandria C, Bartolazzi A. Galectin-3 and cancer immunotherapy: a glycobiological rationale to overcome tumor immune escape. J Exp Clin Cancer Res. 2024;43(1):41. 38317202 10.1186/s13046-024-02968-2 PMC10845537

